# Supplementary material for: Genome-Wide Identification of DREB Gene Family in Kiwifruit and Functional Characterization of Exogenous 5-ALA-Mediated Cold Tolerance via ROS Scavenging and Hormonal Signaling
Source: Plants (Basel). 2025 Aug 17;14(16):2560. doi: 10.3390/plants14162560 (PMC12389587; doi:10.3390/plants14162560)
Supplement: Supplementary file 1 [file plants-14-02560-s001.zip › Annexed Table S1 Physicochemical property data of AcDREB gene.pdf]

Annexed Table S1 Physicochemical property data of AcDREB gene

Sequence ID	gene name	Number of Amino Acid	Grand Average of Hydropathicity	Subcellular Localization
Actinidia16565.t1	AcDREB1	141	-0.416	Nucleus.
Actinidia23663.t1	AcDREB2	142	-0.32	Nucleus.
Actinidia12165.t1	AcDREB3	234	-0.808	Nucleus.
Actinidia33182.t1	AcDREB4	345	-0.587	Nucleus.
Actinidia20766.t1	AcDREB5	257	-0.92	Cytoplasm. Nucleus.
Actinidia13113.t2	AcDREB6	386	-0.627	Nucleus.
Actinidia10399.t1	AcDREB7	665	-0.196	Chloroplast.
Actinidia07971.t1	AcDREB8	160	-0.589	Cytoplasm. Nucleus.
Actinidia25441.t1	AcDREB9	226	-0.4	Cytoplasm. Nucleus.
Actinidia25446.t1	AcDREB10	126	-1.148	Cytoplasm.
Actinidia25486.t1	AcDREB11	327	-0.884	Cytoplasm. Nucleus.
Actinidia25518.t1	AcDREB12	213	-0.972	Nucleus.
Actinidia26001.t1	AcDREB13	382	-0.816	Nucleus.
Actinidia26026.t2	AcDREB14	549	-0.74	Cytoplasm. Nucleus.
Actinidia08804.t1	AcDREB15	187	-0.575	Nucleus.
Actinidia08932.t1	AcDREB16	396	-0.964	Cytoplasm. Nucleus.
Actinidia08910.t1	AcDREB17	223	-0.925	Nucleus.
Actinidia35067.t1	AcDREB18	291	-0.421	Nucleus.
Actinidia35241.t1	AcDREB19	232	-0.598	Nucleus.
Actinidia35366.t1	AcDREB20	210	-0.616	Nucleus.
Actinidia35367.t1	AcDREB21	250	-0.594	Nucleus.
Actinidia35451.t1	AcDREB22	305	-0.347	Cytoplasm. Nucleus.
Actinidia01945.t1	AcDREB23	343	-0.741	Nucleus.
Actinidia04453.t1	AcDREB24	245	-0.555	Nucleus.
Actinidia26237.t1	AcDREB25	353	-0.682	Cytoplasm. Nucleus.
Actinidia13612.t1	AcDREB26	430	-0.85	Nucleus.
Actinidia13611.t1	AcDREB27	250	-0.616	Nucleus.
Actinidia34076.t1	AcDREB28	288	-0.375	Nucleus.
Actinidia00960.t1	AcDREB29	256	-0.499	Nucleus.
Actinidia00973.t1	AcDREB30	284	-0.8	Nucleus.
Actinidia01043.t1	AcDREB31	234	-0.423	Nucleus.
Actinidia01044.t1	AcDREB32	247	-0.493	Nucleus.
Actinidia03102.t1	AcDREB33	252	-0.489	Cytoplasm. Nucleus.
Actinidia03155.t1	AcDREB34	347	-0.572	Nucleus.
Actinidia36471.t1	AcDREB35	146	-0.651	Cytoplasm. Nucleus.
Actinidia36473.t1	AcDREB36	309	-0.763	Nucleus.
Actinidia16620.t1	AcDREB37	360	-0.539	Nucleus.
Actinidia09869.t1	AcDREB38	438	-0.623	Nucleus.
Actinidia09751.t1	AcDREB39	312	-0.462	Nucleus.
Actinidia05451.t1	AcDREB40	238	-0.414	Nucleus.
Actinidia39635.t1	AcDREB41	177	-0.571	Cytoplasm. Nucleus.
Actinidia34742.t1	AcDREB42	339	-0.388	Cytoplasm.
Actinidia07617.t1	AcDREB43	278	-0.738	Nucleus.
Actinidia07616.t1	AcDREB44	362	-0.911	Nucleus.

Actinidia07597.t1	AcDREB45	213	-0.423	Nucleus.
Actinidia07595.t1	AcDREB46	243	-0.112	Nucleus.
Actinidia08622.t1	AcDREB47	381	-0.433	Nucleus.
Actinidia04147.t1	AcDREB48	479	-0.64	Cytoplasm. Nucleus.
Actinidia04217.t1	AcDREB49	304	-0.619	Cytoplasm. Nucleus.
Actinidia14518.t1	AcDREB50	922	-0.384	Nucleus.
Actinidia14407.t1	AcDREB51	268	-0.473	Nucleus.
Actinidia20193.t1	AcDREB52	375	-0.383	Nucleus.
Actinidia20249.t1	AcDREB53	174	-0.462	Nucleus.
Actinidia37989.t1	AcDREB54	420	-0.303	Cytoplasm. Nucleus.Mitochondrion.
Actinidia38068.t1	AcDREB55	138	-0.957	Cytoplasm. Nucleus.
Actinidia29544.t1	AcDREB56	297	-0.426	Nucleus.
Actinidia29458.t1	AcDREB57	156	-0.208	Cytoplasm.
Actinidia29390.t1	AcDREB58	260	-0.372	Nucleus.
Actinidia28819.t1	AcDREB59	740	-0.706	Cytoplasm. Nucleus.
Actinidia38486.t1	AcDREB60	96	-0.615	Cytoplasm. Nucleus.
Actinidia38538.t1	AcDREB61	260	-0.369	Cytoplasm.
Actinidia27084.t1	AcDREB62	251	-0.254	Cytoplasm. Nucleus.
Actinidia35862.t1	AcDREB63	340	-0.579	Nucleus.
Actinidia32541.t1	AcDREB64	536	-0.907	Cytoplasm. Nucleus.
Actinidia36843.t1	AcDREB65	239	-0.786	Nucleus.
Actinidia10747.t1	AcDREB66	162	-0.37	Nucleus.
Actinidia10748.t1	AcDREB67	211	-0.684	Nucleus.
Actinidia37093.t1	AcDREB68	217	-0.645	Cytoplasm. Nucleus.
Actinidia25785.t2	AcDREB69	514	-0.222	Nucleus.
Actinidia21025.t1	AcDREB70	281	-0.796	Nucleus.
Actinidia01350.t1	AcDREB71	393	-0.749	Nucleus.
Actinidia30547.t1	AcDREB72	357	-0.378	Cytoplasm.
Actinidia13662.t1	AcDREB73	284	-0.743	Cytoplasm. Nucleus.
Actinidia19931.t1	AcDREB74	334	-0.79	Cytoplasm. Nucleus.
Actinidia19832.t1	AcDREB75	391	-0.545	Cytoplasm. Nucleus.
Actinidia17188.t1	AcDREB76	374	-0.782	Nucleus.
Actinidia09838.t1	AcDREB77	173	-0.691	Cytoplasm. Nucleus.
Actinidia09850.t1	AcDREB78	301	-0.843	Nucleus.
Actinidia06078.t1	AcDREB79	343	-0.879	Cytoplasm. Nucleus.
Actinidia06105.t1	AcDREB80	387	-0.709	Nucleus.
Actinidia12034.t1	AcDREB81	422	-0.748	Cytoplasm. Nucleus.
Actinidia00441.t1	AcDREB82	307	-0.862	Nucleus.
Actinidia31185.t1	AcDREB83	515	-0.691	Cytoplasm. Nucleus.
Actinidia15099.t1	AcDREB84	440	-0.686	Nucleus.
Actinidia33993.t1	AcDREB85	340	-0.563	Nucleus.
Actinidia00898.t1	AcDREB86	214	-0.637	Cytoplasm.
Actinidia06886.t1	AcDREB87	318	-0.793	Nucleus.
Actinidia18299.t1	AcDREB88	199	-0.749	Nucleus.
Actinidia18264.t1	AcDREB89	341	-0.862	Nucleus.
Actinidia18234.t1	AcDREB90	157	-0.721	Cytoplasm. Nucleus.
Actinidia18228.t1	AcDREB91	245	-0.567	Nucleus.
Actinidia39827.t1	AcDREB92	415	-0.481	Cytoplasm.
Actinidia14109.t1	AcDREB93	169	-0.543	Nucleus.

Actinidia14673.t1	AcDREB94	227	-0.339	Cytoplasm. Nucleus.
Actinidia10847.t1	AcDREB95	253	-0.464	Nucleus.
Actinidia27342.t1	AcDREB96	258	-0.523	Nucleus.
Actinidia27275.t1	AcDREB97	417	-0.71	Nucleus.
Actinidia30276.t1	AcDREB98	285	-0.394	Cytoplasm. Nucleus.
Actinidia14855.t1	AcDREB99	338	-0.502	Nucleus.
Actinidia14886.t1	AcDREB100	307	-0.842	Cytoplasm. Nucleus.
Actinidia38388.t1	AcDREB101	301	-0.681	Nucleus.
Actinidia38372.t1	AcDREB102	316	-0.728	Cytoplasm. Nucleus.
Actinidia08136.t1	AcDREB103	331	-0.657	Nucleus.
Actinidia22077.t1	AcDREB104	523	-0.246	Cytoplasm.Mitochondrion.
Actinidia09555.t1	AcDREB105	228	-0.615	Nucleus.
Actinidia00814.t3	AcDREB106	384	-0.533	Nucleus.
Actinidia24950.t1	AcDREB107	180	-0.332	Nucleus.
Actinidia25820.t1	AcDREB108	278	-0.819	Nucleus.
Actinidia09595.t1	AcDREB109	279	-0.752	Nucleus.
Actinidia28438.t1	AcDREB110	276	-0.499	Nucleus.
Actinidia06476.t1	AcDREB111	326	-0.738	Nucleus.
Actinidia06475.t1	AcDREB112	117	-0.644	Nucleus.
Actinidia33507.t1	AcDREB113	153	-0.752	Nucleus.
Actinidia33496.t1	AcDREB114	145	-0.531	Cytoplasm. Nucleus.
Actinidia27956.t1	AcDREB115	208	-0.979	Nucleus.
Actinidia27955.t1	AcDREB116	356	-0.741	Nucleus.
Actinidia18633.t1	AcDREB117	216	-0.752	Nucleus.
Actinidia03284.t1	AcDREB118	413	-0.541	Cytoplasm.
Actinidia03260.t1	AcDREB119	666	-0.748	Cytoplasm. Nucleus.
Actinidia03244.t1	AcDREB120	267	-0.776	Cytoplasm. Nucleus.
Actinidia39895.t1	AcDREB121	122	-0.717	Nucleus.
Actinidia39896.t1	AcDREB122	163	-0.995	Nucleus.
Actinidia26137.t1	AcDREB123	150	-0.456	Nucleus.
Actinidia26138.t1	AcDREB124	275	-0.403	Nucleus.
Actinidia38946.t1	AcDREB125	231	-0.399	Nucleus.
Actinidia13205.t1	AcDREB126	128	-0.398	Nucleus.
Actinidia13333.t1	AcDREB127	224	-0.537	Cytoplasm. Nucleus.
Actinidia08430.t1	AcDREB128	216	-0.453	Nucleus.
Actinidia20414.t1	AcDREB129	231	-0.438	Cytoplasm. Nucleus.
Actinidia30245.t1	AcDREB130	527	-0.164	Chloroplast.
Actinidia11012.t1	AcDREB131	81	-0.552	Nucleus.
Actinidia11051.t1	AcDREB132	426	-0.658	Nucleus.
Actinidia21434.t1	AcDREB133	272	-0.846	Nucleus.
Actinidia05650.t1	AcDREB134	140	-0.274	Nucleus.
Actinidia05668.t1	AcDREB135	286	-0.139	Nucleus.
Actinidia31286.t1	AcDREB136	216	-0.517	Nucleus.
Actinidia05276.t1	AcDREB137	454	-0.661	Nucleus.
Actinidia05230.t1	AcDREB138	207	-0.404	Cytoplasm. Nucleus.
Actinidia31506.t1	AcDREB139	304	-0.497	Nucleus.
Actinidia23158.t1	AcDREB140	238	-0.761	Nucleus.
Actinidia22728.t1	AcDREB141	268	-0.316	Cytoplasm.
Actinidia17567.t1	AcDREB142	369	-0.627	Cytoplasm.

Actinidia11342.t1	AcDREB143	133	-0.735	Nucleus.
Actinidia00670.t1	AcDREB144	298	-0.566	Nucleus.
Actinidia00657.t2	AcDREB145	341	-0.747	Nucleus.
Actinidia31771.t1	AcDREB146	281	-0.484	Nucleus.
Actinidia31772.t1	AcDREB147	259	-0.656	Nucleus.
Actinidia31862.t1	AcDREB148	316	-0.609	Nucleus.
Actinidia25891.t1	AcDREB149	272	-0.267	Nucleus.
Actinidia24986.t1	AcDREB150	621	-0.456	Nucleus.
Actinidia22349.t1	AcDREB159	186	-0.608	Nucleus.
Actinidia22309.t1	AcDREB160	162	-0.54	Nucleus.
Actinidia31668.t1	AcDREB151	175	-0.881	Cytoplasm. Nucleus.
Actinidia31667.t1	AcDREB152	335	-0.464	Nucleus.
Actinidia31666.t1	AcDREB153	423	-0.274	Nucleus.
Actinidia03019.t1	AcDREB154	495	-0.542	Nucleus.
Actinidia06788.t1	AcDREB155	668	-0.748	Cytoplasm. Nucleus.
Actinidia40060.t1	AcDREB156	273	-0.466	Nucleus.
Actinidia40245.t1	AcDREB157	288	-0.952	Nucleus.
Actinidia40302.t1	AcDREB158	192	-0.85	Nucleus.
Actinidia22149.t1	AcDREB161	306	-0.516	Cytoplasm. Nucleus.
Actinidia02044.t1	AcDREB162	246	-0.443	Cytoplasm. Nucleus.
Actinidia02046.t1	AcDREB163	216	-0.575	Nucleus.
Actinidia02048.t1	AcDREB164	214	-0.584	Cytoplasm. Nucleus.
Actinidia02153.t1	AcDREB165	238	-0.386	Nucleus.
Actinidia02176.t1	AcDREB166	343	-0.36	Nucleus.
Actinidia02177.t1	AcDREB167	586	-0.618	Nucleus.
Actinidia02178.t1	AcDREB168	338	-0.669	Nucleus.
Actinidia02179.t1	AcDREB169	247	-0.336	Nucleus.
Actinidia12663.t1	AcDREB170	207	-0.742	Cytoplasm. Nucleus.
Actinidia16248.t1	AcDREB171	345	-0.575	Cytoplasm.
Actinidia37282.t1	AcDREB172	497	-0.655	Nucleus.
Actinidia24184.t1	AcDREB173	310	-0.413	Cytoplasm. Nucleus.
Actinidia11629.t1	AcDREB174	293	-0.368	Nucleus.
Actinidia24561.t1	AcDREB175	416	-0.569	Cytoplasm. Nucleus.
Actinidia16494.t1	AcDREB176	191	-0.418	Nucleus.
Actinidia16472.t1	AcDREB177	422	-0.716	Cytoplasm. Nucleus.
Actinidia16407.t1	AcDREB178	583	-0.755	Cytoplasm. Nucleus.
Actinidia33302.t1	AcDREB179	338	-0.703	Cytoplasm. Nucleus.
Actinidia29985.t1	AcDREB180	258	-0.55	Cytoplasm. Nucleus.
Actinidia15373.t1	AcDREB181	253	-0.579	Nucleus.
Actinidia01091.t1	AcDREB182	151	-0.842	Nucleus.
Actinidia10225.t1	AcDREB183	613	-0.724	Cytoplasm. Nucleus.
Actinidia10308.t1	AcDREB184	316	-0.64	Nucleus.
Actinidia17778.t1	AcDREB185	417	-0.767	Nucleus.
Actinidia02611.t1	AcDREB186	181	-0.558	Cytoplasm. Nucleus.
Actinidia04754.t1	AcDREB187	362	-0.514	Nucleus.
Actinidia03523.t1	AcDREB188	186	-0.441	Nucleus.
Actinidia23090.t1	AcDREB189	164	-0.593	Nucleus.
Actinidia15738.t1	AcDREB190	279	-0.491	Cytoplasm. Nucleus.
Actinidia15778.t1	AcDREB191	174	-0.633	Nucleus.

Actinidia22649.t1	AcDREB192	185	-0.996	Cytoplasm. Nucleus.
Actinidia19292.t1	AcDREB193	444	-0.665	Nucleus.
