# Supplementary material for: Genome-Wide Identification of DREB Gene Family in Kiwifruit and Functional Characterization of Exogenous 5-ALA-Mediated Cold Tolerance via ROS Scavenging and Hormonal Signaling
Source: Plants (Basel). 2025 Aug 17;14(16):2560. doi: 10.3390/plants14162560 (PMC12389587; doi:10.3390/plants14162560)
Supplement: Supplementary file 1 [file plants-14-02560-s001.zip › Annexed Table S2 Protein sequences of 193 AcDREBs and 56 AtDREBs.pdf]

Annexed Table S2 Protein sequences of 193 AcDREBs and 56 AtDREBs

>Actinidia13612.t1
MALPDETSALELIRQHLLDDFAFAESFITNFDPSSSSNSFVNQFSQTSSSESTGSCKIELDSFE
FETRPQINITSPKPSKFSERRPSLNISVPAAKLNIQGADFCGGVPPREAAEAADSGERRHYR
GVRRRPWGKFAAEIRDPKRRGARVWLGTFDTAVEAAKAYDRAAFKLRGSKAILNFPHEIG
SSPESFPPVDTRRKRRREDESEEREKREVKREKAEEEGVTAVAPLTPSNWTAVWDWTDMK
GIFDVPPLSPLSPHPSMGLSRLMLVSEPCLYPGLMANTREGSINEDTTGETITRGEFRQFQQ
ETQQILRDLQQAIAALLPREPYHGVAGLHQERQERDHRGYDRGPVHHNRPPVHEDESSED
EAYAHDVFGGGRDQGDRSQRGRGQGNHDQGGHMFGNFESRDYRMKMDLPSFNGILQIE
GFLD
>Actinidia13205.t1
MARPQQRYRGVRQRHWGSWVSEIRHPILKTRIWLGTFETAEDAARAYDEAARLMCGSRA
RTNCLQPQLVTVFVEPSFGKFDGQIAQVLHGFASTDQTIGKSASQSFGPTSRCPQLASCTTG
GELGPTI
>Actinidia01091.t1
MESGECCSSSTSPEKRKQRQNQRDNKPYKGIRMRKWGKWVAEIREPNKRSRIWLGSYSSP
VAAARAYDTAVFNLRGPSARLNFPEFITEEDDLRGGGSGADLSAASIRKKATEVGARIDAL
ETAHHHAPAESNWGRASVKPDLNEVIGEGL
>Actinidia16248.t1
MLQALSAISVLCKLTPIYLDHVGRGKSRQPDEREREKVFGEAASTTLETEKLFSAVSYLDK
RFAEISIHFRQMELHFQKQQKEIFSATKQSTMRKKSASNKSKFVGVRQRTSGKWVAEIKNT
TQKIRMWLGTFDTAEEAARAYDEAAFLLRGSNTRTNFVDPGPANSPLSLKIRNLLNQKRA
SNQNLTFSPLPTTTTNNIATTSKTISTIIEKSQSSNNRLVSSILKKDTHMFDDTYKPDLSPLEM
GYSQFTHPFGTEFDRFLLNRSNSVLEGLEVPKRIGLAPQTNSFEMDGDRIPEFEHMKVERQ
ISASLYAMNGVNEYWENVHDSGDAFWDLPTLCQMFCPN
>Actinidia10747.t1
MAFIDAHNDKWLSPTTQPQSPPLARRSPTLSLVAYICYHTDLQQALMTPQRQSIFIIFGFTC
GSRLFSAMGRARAMAAGEADGSGPAKEVRFRGVRKRPWGKFAAEIRDPWKKARVWLG
TFDSAEDAARAYDAAARNLRGAKAKTNFPLSSILAPSLHTRRS
>Actinidia09850.t1
MNLSTNSSKSKNKQTQQQEEKAGRSNRFLGVRRRPWGRYAAEIRDPSTKERHWLGTFDT
AEEAAVAYDRAARSMKGSRARTNFVYSDMPPGSSVTSIISPDDHHHPPPSHHDHPNPNHYF
SSASLHFHHVPPHRPPPLSQPGDTISSHELFVSHNTTRTTSTEFPCHFYPDGFSGNTWATSSV
IENGGCRQFYNDGSDLLPLPPQVSCWEDIGDLDYAGRTSTRGDGGVWTDTPTLHENQNTT
FITTPTVQFGPDESGSYPGFDSTKYLHSPLFGQMPPVSDSDIAFEHLDLGGSSTTFFY

>Actinidia38538.t1
MDLGPTSSLQKGSRRRGKGPYVGVRSRGGRWVSEIRIPKTKTRIWLGSHHSPEKAARAYD
AALYCLKGEQGSFNFPNNRRPDLANRSVGSLPVDEIQCIAAEFSCFDDSVATSEPFPMSPLI
EAHLSPEPPVTPENVNEMEANKVDDEPYFPAFMQEEEPYVPTFVPEEPYVPAYVPEASPM
YKKSFLLRWFVNHADETIYSASVSHRFPLVFSLHELLQYSYHHLLVLFELLDLLSSFLMDF
GRVLQETYGDLHLKLSR
>Actinidia27342.t1
MERPSLPKFNAVGPRVVRITVNDGDATDSSGDEDGLLRRQRVKKFVNEVEVEFRTKYRGV
RQRKWGKWAAEIRHPLRGVRLWLGTYATAEEAALVYDHAAIQLRGPHAQTNFPKPPAKT
TFTGDISGDESHGHSLSSPKSVLRFQIAPNEEVESSPSQPNDAVSQCRDINDATNASENSGFS
PLESLFNCDAFDFQNQVPDLFDGSGFGDIIFSQDCVGPFLESGYEFGFGQSIWATEDCFKFQ
DIGDIFGSDPPITI
>Actinidia16472.t1
MWDLNDSPDQTRDEESEDENGKRVGSVSKSSSSSSVVEEVSDDDGGERGGRKRSSRIFEG
SVASEIESPAVTQQFFPSEVGTTSGVGGDVGGGGGGVSDFPRAHWVGVSFCHSAALSEGC
GMGKSVELGQPPKKSRRGPPSRSSQYRGVTFYKRTGRWESNIWDCGKQVYLGGFDTAHA
AARAYDRAAIKFRGVEADINFSLEDYEEDLKQRSALTKEEFVHVLRRQSTCFPRGSSKYR
GVTLHKCGRWEARMGQFLGKKSVYLGLFDSEMEAARAYDKAAIKCNGKEAVTNFDPKS
YEDELNSAECSGNPTGQNLELSLGSSGSNRNSQESGRSVQHVVSDQRPTGMPFEVDWHD
QGFRAKIQYPSSSNACNGGDLSLSTINPQWQLSPPQLFATAAASSGFPQQITGPQKFATKM
GFTLS
>Actinidia21434.t1
MCGGAILTDFIPRRVSASPLWPASPFATKFNPKDQQPTFKRPQPNTGDGQVENTVKRQRKN
QYRGIRQRPWGKWAAEIRDPRKGIRVWLGTFNTAEEAARAYDREARKIRGKKAKVNFPN
EIHDYVSPTTMPSPNLPLKQTHIEFGENLNQFGACNAWFCSVPLSESEIAYSSMGCDQNEV
KVEEKKKDVRENCQKKALIEVREREEVQDEETEVKKLSEELMAYESFMKFYQIPYLDGQ
SAAAPPNSAPENVDGAATVELWSFDDVTPNGE
>Actinidia19292.t1
MATMGGGNWVSGHYSDHHGVVVDGSGGGGVSTCGDLQSLSLSMSPGSQSSCVISPSTGT
DHCNNMAIETKKRGSAIVAPKQPVHRKSIDTFGQRTSQYRGVTRHRWTGRYEAHLWDNS
CKKEGQTRKGRQVYLGGYDMEEKAARAYDLAALKYWGPSTHINCPLENYQQELEEMK
NMTRQEYVAHLRRKSSGFSRGASMYRGVTRHHQHGRWQARIGRVAGNKDLYLGTFSTQ
EEAAEAYDIAAIKFRGVNAVTNFDISKYDVERIMASNTLLSSELAKRNKDREPKSEAIEYH
HHSSIQNNEELVQSQSNTDNNWKMVSCQSHSQSPQNPRFDSSLDEKLLCIGNYRNSSFLQ
DLIGIDSMSSGQAITMDEPAKLGGVHFSNPSSLVTSLSSSREASPDKNGSMLFTKPPPTVGS

WIPSAQLRAAAAPISVAHLPVFAAWNDT

>Actinidia31185.t1

MVAAAVGGGGWRWKCAAVMVLEVDGGGGGGGGGWRCWRRGWLGGGGGGGWRCW
WWRWWRRWWRWLDGGGCGRGSVAGGGVWCVGGAGAAVVVGGGGGGWLEVEVGG
GGEMEMITVVKSEVSPVRRRLCEMEGELPVAKCVKRRRREPNVLGCTDQEDHQKPQQLQ
VVDQSNTTTTYHYRQEKLKISRHRWTGRFEAHLWDKGSWNATQRKKGKQGAFDEEESA
ARAYDLAAIKYWGPTTFTNFPVTDYEKEIEMMQNVTKEEYLASLRRRSSGFSRGVSKYR
GVARHHHNGRWEARIGRVFGNKYLYLGTYSTQEEAARAYDIAAIEYRGINAVTNFDLSSYI
RWLRPGSNSLPPQDPRINLEPQPINTLCGPISSPEPEFTFRSSPYTMGDYGNNPRKQEVLET
KMPISPCNRSPSPTAVGLLLRSNMFRDLVEKNSNVVDDDSEQNDTKNKSQINDGDEFGKF
FFNSIGSNPYECSSSSDKLPRLESPNENALPFYNKAGKSLWNGALNLPAN

>Actinidia03523.t1

MAVTGFVGYSSNNYVGGAAGGARSIYRGVRKRKWGKWVSEIREPGKKTRIWLGSFETPE
MAATAYDAAALHLRGHGARLNFPELAHSLPRPRGPSAEDVRLAAQEAALRFQKPTSGFE
ASNSSSNLAPVTVGLSPSQIQAINESPLDSPKMWMDLTGVMLEERAVFSKDVEMDEWDEI
TDDSLWDP

>Actinidia07616.t1

MDNKQVLEREIYESDHMINLQDHERDTFLHDKTEGNTEEPCIGMEFESEEAAMVYHDPY
AKCVGNPHRSSRDGSVACRREESAQKTALWHRTFREFQLVHGVFGVSNAIKIMKDLDFSE
QKEAAEAMIWEAEAWEQDPVRGPLGLCGLRNKPKCLRKKSECSGNKIRLDLERENGELP
LNENDSQDMVLYHLLNESNGQNTPIFPSKTQSNARKVLEPTGTIEKKHYRGVRRRPWGKY
AAEIRDSTRNGARKWLGTFETAEEAALAYDRAAFSMRGAKALLNFPAEVGAESSKQRFTP
NLSSRRLDRHNSCSSTSSSEPSLGMSRSESKSTKESSQNSFSDNTNAENVSKSSVLHVQQEF
GT

>Actinidia08804.t1

MVQSMKFRGVRQRQWGSWVAEIRHPLLKRRVWLGTFKTAEKAARAYDEAAILINGPNA
KTNFPTTTANQSTNEKSPFTSSLSLSSSSSSLSSIISTKLRKCCKSPSPSLTCLRLDPDKSHVG
VWQKRAGKCSDSNWVTTVELDKKIEKPLENDVETALTSGGAGGRDGLGEEERMALQMI
EEFLNQN

>Actinidia01044.t1

MMTTTDEFLALELIRQHLLGDFTSIESFISNLNLTASSNGSVLKPVFSFDESDLSPSGSSDSN
SPFHPQITTRPDAFSGSDLEFFEFDSKPEIVDLMLPGPKIFSGSDLNFSETLNPEIADVILPGP
KTLRGSVAAITGSGQMKRHYRGVRRRPWGKYAAEIRDRNRGGARVWLGTFDTPIEAARA
YDCAAFEMRGRKAVLNFPLDAGKYGPPESAGRKRRREGGVEGRREKRGGLLESGGSVEF
EK

>Actinidia03019.t1

MLDLNVDVVTLDSPCDEVATKIASEKLQDGDDSGTSNSSVVSADDAPNNVADEDSSSNQI
APSNNTSTFDFDVLNKGKSTSVGKMAGIGDETDRRNTTPALVTRQFFPVGDLEKIRSGSSS
ATSLLRPQWLNLSFPDTGGGVELRSIQPQKQQVKKSRRGPRSRSSQYRGVTFYRRTGRWE
SHIWDCGKQVYLGGFDTAHAAARAYDRAAIKFRGVDADINFNISDYEEDMTQMKNLTKE
EFVHILRRQSTGFSRGSSKYRGVTLHKCGRWEARMGQLLGKKYIYLGLFDSEVEAARAY
DKAAIKCNGREAVTNFEPSTYEGEMSGVTAQNLDLSLLISLPTDVPKGNDNFRDFQSQCA
AYELSDTKRLKLESSVSAPVDGQNICGLTMAAKRSTIWSGIYPGFTPNYEERALGKAAEAL
PSQPAFSNWAWQVQSHSVVSPAALFPTAASSGFASSPITTLSPPLPLQTPQNKTAHKLFPPTP
ANTISHFYHHKT

>Actinidia00670.t1

MYAPMASSGHHHPTQDKPGPLPTDTTTKTTTTNAAATTRKCKGKGGPENSKFKYRGVRQ
RSWGKWVAEIREPRKRTRRWLGTFSTAEDAAAAYDRAAVFLYGHRAQLNLQPSPTPTSSG
GGGGSSRGGSSSSSSTQTLRPLLPRPPNFSVTFPQYSSTLPSPYSNIYDTVQFSTAVQSPYQIS
QVVPSRTNHVNNDAITAATMVASSSSLLSSYQIPCYDPHSHHHNCLNEQINSLLGSVDSSLS
LSSQQVVEPAVSDPTVAVAETSAVGSPVIWPVTSDDEYPPASIWDYGDPSFDF

>Actinidia00441.t1

MLRQPKRPRHDGASTSQDHQPPRLTPEQEASVIVATLVNVITGATADNPLLPISDADTCQLC
RINGCLGCNFFAPTQDNKKGKNGIVKRKKKNYRGVRQRPWGKWAAEIRDPRRAQRVWL
GTFDTAEAAARAYDKKAIEFRGARAKLNFPFPEQSTLPNEEQSSEEKRPENMAERESEAA
AETGRREEKEFWEVIGEEEIREWMMTLMDFDDNSSDSAATGNVGSHDFSHTLADPVSHS
FSRSQVSFCGRRELVRRIDQSILGSDGVLMRKNPWSRNGSNFSQVILYSTKHPSRNRKMEG
SRGMDIL

>Actinidia25785.t2

MNSQFTQYPYLDDSPESSLGSPQSFSWDDLLFYHNSLPFNVNDSEEMLLLGVLAEGASKE
SSETVSSGGIKEEEVTSVAKDQKPVKEKSYRGVRRRPWGKFAAEIRDSTRNGIRVWLGTF
DSAEEAALAYDQAAFSVRGSMAVLNFPVERVKDSLREMKYGCEEGCSPVVALKKRHSMR
ERSSYKRNKANKEVLFLRLKARTELWYHWCDLRLMAHNIQNYTKESLALINPTNPLLGD
LLSFFFSWQEKFLQPLDWKQTRTGTWKRKKDYLVSDIEENTFMISFRDRDDWIKILLYGP
WTVKGDTKDYLNFVTAVAKLAMCSEHVSSPQAMPSLVHQCELCPMMLLYLVVTVPGLN
QGCLIGGVQVCTSLVALPPPKAIMKQSMSGIWPRPRMESGSGQNCAAGVTRRYLWQKLII
DIIDSIAMVAGYIFYLLLKLTRIVRNSCYLQGYDARVPLAEGKDGTCRSPHFRSIVSGKVFL
TLQVKSFLSIVTRCIVSNVNMDGIVSLHGSYTMSQN

>Actinidia06475.t1

MSLFYPYSCRESEGKISGSRFFEGKKDPKKSEKRYIRVRNRTWEKYAAEIRDTTRHGARV

WLRTFTTAKAADLACDQAAFVMRRWLAWRNFPTDKSVNRFVISNVGADLLDELLSSS

>Actinidia33302.t1

MVGVLEWVIEAEEDGGGGIGSKGMWRNCGGGRRCNGDNNSSGDVIHGGSSLNIEQLTQE

RNWHRWTGRYEAHLWDKNCWNESQNKKGRQGAYDDEDAAAHAYDLAALKYWGPETI

LNFTVETYKQELIQMEGISREEYIGSLRSRHHHNGRWEARIGRVFGNKYLYLGTYATQEEA

AIAYDMAAIEYRGLNAVTNFDLSRYITWLRPNNNNNNADDSTLTLTPTLNPNVETTITDPS

PNRELGLNQQPLITTFVTPAQPRPTTATSALGLLLQSSKFKEMMEMTSADECTFTPSELGPP

QCSSSDDHMPTYFESQDFGVYDEGDDGIFRDLNSFMFDA

>Actinidia18264.t1

MAKISLKNGSNTRSSSSSNHNTATKTKRTRKSIPRDSPPQRSSIHRGVTRHRWTGRYEAHL

WDKNCWNESQNKKGRQVYLGAYDDEEAAAHAYDLAALKYWGNDTLLNFPLSTYQEEL

KEMEGQSKEEYIGSLRRKSSGFSRGVSKYRGVARHHHNGRWEARIGRVFGNKYLYLGTY

ATQEEAATAYDMAAIEYRGLNAVTNFDLSRYIKWLRPNQNNTNPQPNPTPSNETGLSFPHQ

QSCSAAAAGATTPPPHPATCTTASSALGLLLQSSKFKEMLERTSATDCPLPPPKSDPPRSSFP

DDIQTYFHCQDSSEYADGDDIIFGDLNSLASPIFSCELDA

>Actinidia31666.t1

MDMYGHECTPVIFPASSSVEFPVYHGSSIFSSLIPCLTEKWGDLPLKVDDSEDMVIYWLLR

DAVSAGWTPFNLTATDVKPETRDEFEPPTTTSAAQSVESPVTEVPAASLPKGRHFRGVRQR

PWGKFAAEIRDPAKNGARVWLGTYERAEEAAMAYDKAAYRMRGSRALLNFPHRIGLDEP

EPVRVTPKRRSPEPTSPASESCSPKRRKKGLAAADRAELGVERESSVSQVGCQARQLPVGE

QLLLHPAVARTVLRHYLYPTPLGLPISHRRRKWWWAAGGSKYRQSRVMIRLKMEFFWIPV

PPGGEGRNGGHRSLLVLSGLTLACTVIALTIATPLLVIFSPVLVPATITVSFLMTGFLASSGFG

VAAVTFLSWIYRYVTGKHPPGADQLDQARQKLASKAREMRDRAEQFGQQHIRGSQAS

>Actinidia03260.t1

MAYMNNWLGFSLSPQEHQNPQDHSQNSVSRLGFNTDKISGTDVSSDQCFDLTSDSTVLPS

LNLPAPFGILEAFNRNNTHSHSQDWNNNYKTTTDFSMLMGTNNHSLENQGPKLENFLGS

HSFAHHGHYMYPPLEAAETATATTTTNGANNNNIGLSMIKSWLRNQPTPTPAQPENKNDQ

GCGVEGSNGNLSLSMSTESQTGSPLHLLAVSGGGSGGGESSISLDSKQKGTSLDAQTGAIE

AVPRKSIDTFGQRTSIYRGVTRHRWTGRYEAHLWDNSCRREGQTRKGRQGGYDKEEKAA

RAYDLAALKYWGTTTTTNFPIGNYEKEIEEMKHMTRQEYVASLRRKSSGFSRGASIYRGV

TRHHQHGRWQARIGRVAGNKDLYLGTFSTQEEAAEAYDIAAIKFRGLNAVTNFDMSRYD

VKSILESSTLPIGGAAKRLKDAEQAEMSMDTQQIRTTDEGITTSHLTDYGTTLHNWPTIAF

QQAQPFSMHYPYGQQRMWCKQEQDEIPTHTFQDLNQIQLGNTHNFFQPNNNVLHNLMS

LDSASLDHNSGSNSGTYSNGVGDGGYVMPIATVISQEGNNTQGNSGFGDNEVKAYGYET

VFGSSDAYHGRNLYYLPQQSQGCAGKVASVYDQGSTCNNWVPTAVPTLAPRANNMALC

HGSQTFTVWNDT

>Actinidia37093.t1

MNLEDESSPYSSSSSSSSTFCNVTKNPSSPSTPTNVSLPTFHKRKAGRKKFKETRHPIYRGV

RLKNRNKWVCEVREPKKKSRIWLGTFPCPEMAARAYDVAALALRGEMAKLNFPDSTTLL

PRAKSSSSNDIQMAVLEATRDFQLTIPSPSSCSSQSSRGTSLTNLTRPRSTHIPHAVSEDSSIN

MAEGLLLTPPAMKRGFNWDAMDCDMDMTLWRD

>Actinidia00973.t1

MSPEIERIIRMGFLDQASNMVSVPLDHSRKRKSRSRRDGPNGIAETLAKWKEYNEKIESLD

NGPKPARKTPAKGSKKGCMKGKGGPQNSHCNYRGVRQRTWGKWVAEIREPNRGSRIWL

GTFPTAPEAARAYDEAAKAMYGPGARLNFAEGSSLTESSVQSASLPTTSGSGSSTSSSYSE

VKLHAPKAKHDDEDDELRIDGNSSYAGNSVPMTAVKEEVNDEPVDFSGKGKQMELFEKP

SLDDILQNFSLEMLDAEELLGILDTDYNMGPAHNSGKMGGNLVIG

>Actinidia25820.t1

MCGGSILEELIPRNGNHRVSASQLWPNSPFVTKFKPPQDQNDGDERVEKKAKRQRKNLYR

GIRQRPWGKWAAEIRDPRKGVRVWIGTFNTAEEAARAYDREARKIRGNKAKVNFPNEDD

HSIQFTPQTHHLPTAMSHPNGGFSGNLNQFGAYSSNGFNSVPCSDPVSVLHFEEISGSGLES

AYSSIDCKLEVKEEREKQEERGNRKEAAVMEVEEAAGEESEVEKLSEELMAYESVMKFY

QIPYLDGQSTNAPPAENDVIGCGAVELWSFDDLTPTVA

>Actinidia26137.t1

MSSSREGHYRGVRKRPWGRYAAEIRDPWKKTRVWLGTFDTPEEAAMAYDGAARSLRGA

KAKTNFPQPLPPPELPLDLNLAADHSWASPARRLVIGEFLHTGVLKEVGSNCELAVNSGGV

GVGAGEVPATERSAPAGYFGVLRQAGKKLTS

>Actinidia08622.t1

MDQSMSNLWPIKYTEHKSVTKKLIKPSNLKPRKASGGPKVLDYTSGVAPRVVRISVTDPD

ATDSSGDEEAQFLGRQRVKRYVNEISIESAANKSGVKRKGMKKAAAASGGGFQKYRGVR

RRPWGKFAAEIRDPMRRTRLWLGTYDTAEEAAAVYDNAAIRLRGPDALTNFVTPPPKDAA

DVNMTSVSGYESGYESRSLSSPTSVLRFTSLSTEETKPSKPVQEDPVIPEPIQGSEEFQDETS

LSTQIVDYLPMDLSLVDDFFNFESSEPILLDETPTLPNDLLRDDYLSDVFMDSGNDFGSSLS

IWQADDYFQDLGVDLFTSDPLMALSLPLLDARTCRGCKVLQIKMKQLTSSFVGIGRLIIGH

LVIKKSLQQIIKFQSK

>Actinidia18299.t1

MVSALSQVIGSNTSKNPVHLHENSLPEPNQSHLPSQYQGNQKRRHYRGVRQRPWGKWA

AEIRDPNKAARVWLGTFDTAEGAALAYDEAALRFRGNKAKLNFPERVQARTDFGYLRNH

QEKNYFDLLHYARLLRCGGSGMNNDVSGIYPRGTFVSQPLSAISPASASTSQQQQGEVMR

FGETILHPKPQGDIIKEGNYLM

>Actinidia02044.t1

MLQDRISRRPNVLNFRVLEPTTSSFDPSPPPPPSPPAIMCQQEQSPSQHSISITTTNIECENAL

SPKTGKILPLTAASSTATRKHPMYRGIRSRSGKWVSEIREPRKTTRIWLGTYPSPEMAAAAY

DVAALALKGSDVIINFPEKVKSYPVPETKSPADIRAAAAAAAALMKAGSSENPLGNRDST

AGVAAAAPGDEFIDEEALFDMPSLLVGMAEGMLMSPPRMHSPRPEDTPENSDGESLWSY

D

>Actinidia25446.t1

MARPQQRYRGVRQRHWGSWVSEIRHPTLKTRIWLAHRDGRGTRPSRRRAASSSKLQRCH

MTSQQVSKKTKTKEPHPHDAHRHHVLGNEGQAGSEQQFKALEDDHIEQMIEELLDYGSI

ELCSVVPN

>Actinidia22077.t1

MPKFKGIRGRGNGNNSNKLVGVRQRSSGRWVAEIKDTTKKIRMWLGTYETAEEAARAYD

EAACLLRGSNTRTNFITHVTHDSPLAFRISELLNTKKSGKKLSENIYGDTNSSYTHTPPISTT

ANIPSISTGSCTISTSDNETTSSGKLQDTQLFDDAYRPNWSNCLEEFELGTSQSGMFGHRFD

QFPFNQELLELPRNVVSHEASELEFSEFDRIKVERQISASFYAMNGVQEYMETVHDPSDAF

WDLPPLWKMEKEKAKSEGVWPTVKPFVNGGTAGMLATCVVQPIDMIKVRMQLGEGSAV

QVTKKMLQNEGMGAFYQGLSAGLLRQATYTTARLGSFKVLTKKAVDANGGKPLPIYQK

ALCGLTAGAIGACVGNPADLALIHEGGLALWKGAGPTVVRAMALNMGMLASYDQSVEF

FRDSLGLDETKTVIGASAVSGFFASACSLPFDYVKTQIQKMQHDAEGKYLYTGSLDCAIKT

LKSGGPLRFYTGFPVYFVRIAPHVMLTWIFLNQIEKLEATIGL

>Actinidia10225.t1

MKSSNDNNNGSSSGNTNWLGFSLSPHMKMETTSDPHHQYNHQSQPNSYYISSPSHLNTSS

IYYPPLCVMPLKSDGSLCIMEALTRSQSEGAVPNSSPKLEDFLGGATMAAHQYSSQEREAA

MALTLDSLYYHQNPEVHQQEQEIEAQNNPYYSGLPCNGMLYHSPLEVQTKDTQMATMD

GNWVSGHYSDHHGVVVDGGSGGVGTCGDLQSLSLSMSPGSQSSCVISPSTGTDQCNNM

AIETKKRGCAIVAQKQPVHRKSIDTFGQRTSQYRGVTRHRWTGRYEAHLWDNSCKKEGQ

TRKGRQVYLGGYDMEDKAARAYDLAALKYWGPSTHINFPLENYQQELEEMKNMTRQE

YVAHLRRKSSGFSRGASMYRGVTRHHQHGRWQARIGRVAGNKDLYLGTFSTQEEAAEAY

DIAAIKFRGVNAVTNFDISRYDVERIMASNTLLASELAKRNKDREPKTEAIECHHHSSTQN

NEELVQPQSNTDNNWKMVSCQSQSPQNPRFDSSLDEKLLCIGNYRNSSFLQDWIGIDSMS

AGQAMNDEPAKFGGVHFSNPSSLVTSLSSSREASPDKTGSMLFAKRPPTVGSWIPSAQLRA

AAAPISVAHLPAFAAWNDT

>Actinidia38372.t1

MPEPPPNQRNICKKPKKKTVSAEENTRKMKKKIRLIFSDPDATESSDDERSGFEPKRIVREI

NLPLAILNLPKAPESENSCQDSNNGENPSHRKRVSPKFPSSKIPDFRQRKRDRWAAETRDP

VSGKRLWLGTFNLPEEASEAIERKKIEFAAMAGDKSCYNSFSVAVSENSKTSSPCVLESDSV
ASQSYTKCDETVKDADADTNLGEKKGIDAGADTNLGEKKGIDAGADTNLGEKKGIDSVE
LDLGMELDSLFVGDFDNVLGDFCGLDDLQLCGFEDVEQIDLPDFNFELGSEEFAWIGEAIN
IACTSFAASRN
>Actinidia31862.t1
MDESMKATGVVLRWTQSCTNIGHHPDATDSSSGEDDAFFGRRRVKRYVNEISFETACKSS
LNANQVWKRKVGTGNSLLAKRKPTKSAAARGGGGQKYRGVRQRPWGKWAAEIRDPVK
KVRLWLGTFDTAEEAAMVYDNAAIKLRGPHALTNFITPPLSESPDINVTSVSGYESGDESH
THSLSSPTSVLRFRTHSNEEAKPLKHSKPAQEGSESNENSPVHEPKPCQEPVQEDTCFLPDF
IPMEMPFLDDLFNFEPPGPIHFDDTPVFADNLLGDDLSGMFTDPIHDFDPSSTWQVDDLFE
DIGGDFFTADPLIVI
>Actinidia03284.t1
IRIKSTRKEDNFLNTGDFGSNIRLLLQILHIIIKVYKSRAKGENLAFGTAYRAPKLNRTKGFI
EFSRVISGFDWTRFTMEEEMSTASNAISSSTKETSDSNSNNLPLHRNKCPKEDITMLASKFK
GVVGQQNGHWGAQIYANHQRIWLGTFKSEIEAAMAYDSAAIKLRSGDSYRNFPWTNVTF
QEPNFQSHFSTEEVLIMIKDGSYPSKFADFLRIQFPCGDNVDGLNLVRRPGKGFSCRQLFQ
KELTPSDVGKLNRLVIPKKYAVRYFPRISEDGNENLINGRMEDVNLVFYDRLMRLWKFRY
CYWKSSQSFVFTRGWNRFVKENELKEKDMVVFSVCECREGSNQIQAFCMIEVAFKDRVE
SNGSGVEDNNEVAQVLQEELKLDIQQDNGAGEEDSEECELGLHLWQNIR
>Actinidia31667.t1
MLPTTSIEATPHLLALAQIPPFIILSPSPIFPLIPNFEFQLPPPSFPLTITVLSPMASPDQTSALDL
IRQHLLDDFAFAESFITNLDSSSSSNSSENQFSQTSSSESTGSCKIELDNFEFETKPQINITSPK
PSKFSERRPSLNISVPAANLKIQGKDLCSGAPPRVAEEAEDSGQGRHYRGVRRRPWGKFAA
EIRDPNRRGARVWLGTFDTAVEAAKAYDRAAFKLRGSKAILNFPHEIGSSTESFPPADTLR
KRRREDESEGRERREVKREKAEAEADGVSAVAPLTPSSWTAVWDCTDMKGIFDIPPLSPLS
PHPSMGLSRLMEHLTAKGLR
>Actinidia05230.t1
MNKKVPVPGTIDRGDPHAQRTIAPTDTQSRKPLYKREKTMSGCGAGRQATGKHPLYRGIR
SRSGKWVSEIREPRKTKRVWLGTYPTPEMAAAAYDAAALALKGGDAVLNFPSCVGSYPV
PPSTSPADVRAAAAVAAAMMAEGGAGSGAMEGGPVGGNEFVDEEALFDLPNLLVDMAG
GMMMSPPRIESLPSDDSPGNCDGDRLWSYV
>Actinidia13611.t1
MTTTDELLALELIRKHLLGDFTSTESFITNLNLSPAASSDNSDLSLSPADSNSFFSDPKYSGS
PTSPFDCLNPEHDFPEFESKPEIGNWMVSVPKNRSFTGTGQRESGNKSERRHYRGVRMRP
WGKFAAEIRDPTRKGSRVWLGTFDEAIDAARAYDCAAFKMRGSKAILNFPLEAGKSDPPA

GNGRKRRIEGGAERRPKRVEVASPEWDDGGSSGFPLTPSSWAAVLEGVSGFSEGDVGSPFS
PRILAL
>Actinidia37282.t1
MLDLNLSVVHNENDSMEITEKVPEDSVGQMADSGTSTSSVANAEGSSNADDDSCSTRAS
GGDIVAFNFDILKVGDTSEEGSENRMTQSEFTRQLFPVSGDGGLTTGHSWASSAVSQTNQV
DLSFNRASPNGLREVRVPKQQQQAKKSRRGPKSRSSEYRGVTFYRRTGRWESHIWDCRK
QVYLGGFDTAHAAARAYDRAAIKFRGVDADINFNLIDYEEDLKQTKNLTKEEFVHTLRRQ
STGFSRGSSKYRGVTLHKCGRWEARMGQFLGKKYIYLGLFDSELEAARAYDKAAIQCSG
REAVTNFVPSTYEGEMIPEANAGGRDHNLDLNLGISTPSSGDAQKDHESLGHLPFHPYIVH
DARKSNMKDVSATTVGSPTVKGLPMTSEHLPLWTGVYPNFFPICEERETGKRIEGGSSQGS
PNWVWQTHGQVPSTPLTFSTAASSGFSSASTTAFSAFVHPSKPPNPTPLNLYFSSPAPASSNS
QYYCQMRPLQPPPS
>Actinidia39635.t1
MVKPETSKQAERSTSDESKYKGVRKRKWGKWVSEIRLPNSRERIWLGSYKSAEKAARAF
DAALFCLRGPSAKFNFPENPPDIAGGRSLTPAEIQVESPSPSVSDGVAPMGTETPLDGSFLN
LFATMGSENTGPGSFDIFPGFDDFSNEFFVPPLPNVDYGEENCDGNFSQGSFLWNF
>Actinidia10399.t1
MSCYKGKYHDELIANAAYIGTPGKGILAADESTGTMGKRLSSINVENVESNRRAFRELLF
TTPGALEYLSGVILFEETLYQKTSDGKPFVELLKENGVLPGIKVDKGVVVLPGTNGETTTQ
GLDDLGKRCQQYYAAGCRFAKWRAVLNIGITEPSQLAINENANGLARYAIICQENGLVPIV
EPEILVDGNHSIDKCADVTERVLAAVYKALNDHHVLLEGTLLKPNMVTPGSEAPKVAPEV
VAEYTVRALQRTMPAAVPAVVFLSGGQSEEEATLNLNAMNKLKTKKPWTLTFSFGRALQA
STLKAWQGKVENVEKAMAVFLQRCKANSEATLGTYKGSGSLSEGASESLHVKDYNVRG
WGQEYLHPQNSCCYGFGFLLMCSIMNEHQLAVFVFLALVIVRRALRASRLQLTGRQDEER
FHGFISPTGFERQIAGLWAIHILGWGSLDLGWVPLGYSPGEPLCSLRHWNNPSSGGNGGG
GSGRFDMGSTRVSGRHPVYRGVRRRKSGGKWVSEIRELRSPNRIWLGTFPTPEMAAVAYD
VAALALRGSEAELNFPGSAFSLPVPASTAARDIQMAAASAAAAVGAVVDSFSESQNDHLD
KYATSQESENPIGFHEFFDEDLIFDMPNVLVNLAQGMLLSPPRYDFAGEDNGDENTGDQNL
WKFS
>Actinidia34742.t1
MELFADVTPKTAENFRALCTGEKGIGISGKPLTYKGSAFHRIIPNFMCQGGDFTRGNGTGG
ESIYGQKFADENFKMKHTGPGILSMANAGPNTNGSQFFICTEKTSWLDGKHVVFGKVVD
GYSVVKEMEKVGSNGGTTSATVVIEDCGIENQTLPFNENDSEEMLLFGVIAEGDSSTTDSS
MGTSSEEVCSEASGDVSKEVAYRGVRRRPWGKYAAEIRDSTRKGVRVWLGTFDTAEAAA
LAYDQAAFAMRGSMAVLNFPAEVVYKSLRGMECGFEDGGSPVLALKKKHSMKRKGVRG

KKKEEKQNQLRMENVVVLEDLGPDYLEELLGLSESTSVGTW

>Actinidia10748.t1

MGRARAVAAGEADGSGPANEVRFRGVRKRPWGRFAAEIRDPWKKARVWLGTFDSAEDA

ARAYDAAARSLRGAKARTNFPPSSSILAPLSPHSPFDQNSHDPFIERRLYPPHHQIIPQRPTS

SSLSSTVESFSGPRPPRPAVAPPSSSRRRPRTPPVSPDDCRSDCDSSSSVVDDCDGDIASSLFR

KHLDFDLNLPPPLDVADDLYCTDLRL

>Actinidia25441.t1

MNHPPLQQIPFTPRPDPFLFKPTQTFPRLTEFPLVCYEQPLFPSSLSFSKNNPPDHIPEAPTVL

EGIAAVVGQHVLFGKGDKPGGDSVTHRGSEEKAVGRWSAEIRDRVGRCRHWLGTFDTAE

DAARAYDAAARRLRGVKARTNFTIPPVLPLLSPSSSSSSVAKKKKTRGKAASQNRLKCAV

VTSVGQLFRNPITHTSRPPVELDLKLGASFSHSSSMALLSTAN

>Actinidia36843.t1

MTAMVSALTQVITSTHHHKTTPLDDDLLPPNPLALPRSGAPETNPSQPGQDKGNQQRRHY

RGVRQRPWGKWAAEIRDPNKAARVWLGTFDTAEGAALAYDEAALRFKGNKAKLNFPER

VQGRIEFGYLMIRPDSRVVANRVLDSLPPPPPPRHQIRSQEPHPNLHHYAQLLHSGGNEVN

YTVSGANPRGTLAPSSSTTTSQQLQGQVVRSFPILQLGSSSSGSDFPSNWGDFDSNNSRR

>Actinidia31506.t1

MATTDEISALELIKLHLLGEVSPVGKFVAELSEVSSSGSDSLSSQTSSCDSQITISSCVNSNE

VNDTDLFDFDPYSVDFFEFESKPQTIDLATPRPVDLSPKSSSQSRLVERRLKIDLAPVKPPVT

KLEWLEFSEQAAEVSEQKPSEAEERQHYRGVRRRPWGKYAAEIRDPKRRGSRVWLGTFD

TAIEAAKAYDRAAFEMRRSKAILNFPLEVGKSNDTAAAAPVDGGRKRTREVEETTEEMPA

KKEKSTESDASVLSNLTTCPLTPSSWMTVWDPNGSGIFNVPMLSPLSPHPMMGFTPLTI

>Actinidia33182.t1

MENKFPTMEKEFPSYLQVMATGSRVCLDAANVLGIANETLCRDGGGGSSSSVKLSSSSSD

RIFSSLESNSPTETLSTSPHTNAHGGYIPLNFLKSFEPSSPPSSSSPLKSPNLGQSQLQQGIEW

IKINQNTTNYSSKGFSDYWLSTTKTQPMKYIGRRMQPPNYQKATLPSTSPCERKLFRGVR

QRHWGKWVAEIRLPRNRTRVWLGTFDTAEEAAFAYDTAAYILRGDYAHLNFPDLKHQLA

SSANCATKALLEAKLQAMSGNKKAIDPQPTSPKKDLFKGLSHIDLTRREWEFDLESKAGS

EMVMIENKKKSTQELLSDVEAVQLSRIPSLDMDMIWDALPDS

>Actinidia27955.t1

MVAALSAVISGSTSLPEAGPHLLPIILEPDTCQFCKIDGCVGCNFFPPSPSDNNNNNNNISSS

NHASNSSPSHHNKKGGKRRKKNKYRGVRQRPWGKWGVEIRDPRRAARVWLGTFETEEE

GARGLTTRQRSSFHTGPGAKLNFRSRGEGGELAPPRGQGSELSGREKILKMSKPSFLEFQY

NLSRRKYLRKPTRMFSFGDGQNTSLLPAYQPNSAEMKKVFNKFDSNKDGKISPEEYKAIL

RAIGKEHMIKDVGKIFKVADLDGDGFIDFKEFVEVHNKEGGVKTMDIQQAFQAFDSDKD

GKISTEEVFELLKRLGEKCSMQDCRKMVRAVDTNGDGVIDMDEFTTMMTRTMKLC

>Actinidia07617.t1

MIAPRGEMAVEEQHDHKSSEAILENVWASYIAQNEPNTLELSKEWEEMPSLDGRDGFME

VLQRLPSLGRWISMGAEAWEELLGGINCPPSNTEELCNPEIITSDDHDNSNSKSCEKRVEK

ARPRHYRGVRMRPWGKYAAEIRDSSRKGSRVWLGTFETAEEAALAYDKAALRIRGPKAH

LNFPLEAIASKDMEIPCLENDTNPSSTIFRGKDSSSYEPFYRCGERISDSRKRVSGESWENE

DLGVSGESVLKRMATIEQKIGDGYDVLEFQGLGSDYL

>Actinidia02611.t1

MVKSEHKIQTQPNKAMPSSSSSSSSMIKKKYKGVRMRSWGSWVSEIRAPNQKTRIWLGS

YSTPDAAARAYDAALLCLKGKSVNLNFPTSSSFFHHHHHDTVLSPKSIQRVAAAAAAANS

FNDTTNPPPPPLPSTSSSSSTTTTSSSSCSASSPSMSSSPSNHIEDDFSLASSLDDYTLWMK

>Actinidia11012.t1

METHYRGVRKSKKRLVGKYGAEIRDPKTSKRVWLGAFDTAEAAAKAYDAKAIEFHGPK

AKTNFIYPLRDMYFKIALDLCDA

>Actinidia12034.t1

MWDLNDSPDPTTRNDESEVCSYGDDDKGKRVGSVSNSSSSAVDDEDAGGQKRSGSKIFG

FSVTQPDDTYSSEPDPPVTHQFFPSSDDGGAANFPRAHWVGVRFSQSEGPAAAAAGKFTN

ITQPLKKSRRGPRSRSSQYRGVTFYRRTGRWESLFGSWVFSCMSISIGGFDTAHAAARAYD

RAAIKFRGVEADINFTLEEYEEDLKQMSNLTKEEFVHVLRRQSTGFPRGSSKYRGVTLHK

CGRWEARMGQFLGKKYVYLGLFDTEIEAARAYDKAAIKCNGKEAVTNFDPSIYEDEFNST

ECSGNPSDHNLDLSLGCSASKQKSRDVAADQHHTSMPFEVSDLRNHGFQPKVQCPSSSTN

DNGGYLSLSTSDCQNWQLNIINNPPQVFATAAASSGFPQQKITTTPQNWLQQNGFHFSMR

PS

>Actinidia13113.t2

MLDLNLTAMPTDSISDGQMDDSGASNSSLLNVETSSVAVDEESSSTHENTFAYSFGILKNY

ESEENNGNREARLGFVSKQLFPASGGGGGIEWLDLSCRAASSGGTSEQRQQMKKSRRGP

RSRSSQYRGVTFYRRTGRWESHIWDCGKQMYLGGFDTAHAAARAYDRAAIKFRGLDADI

NFNISDYDADLKQMKNLTKEEFVHILRRHSTGFSRGSSKYRGVTLHKCGRWEARMGQFL

GKKYIYLGLFDNEVEAARAYDKAAIKCNGKEAVTNFEPSTYEEELSSEAGNGESSHNLDL

NLGIATNYFADGQRGNNDLNSGPHFHCGPTDVHVSRRAENSGPTMATQSPHGQIMVSDH

PPLWRGPLATVALYLLPISGFAFGVNKAA

>Actinidia31771.t1

MYRQESTAVIFPGNIPSNICRSSSFGSLMPYLSETWGDLPLNYDDSEDMVIYGLLHDAVNV

GWTPSNSTVTDVAPEQRYITEPATTTAHSGFYSPLKFDDSEDMILHGLLREAFNLTVTDVK

VEPRHEIEPAKTTAQLVAPSVMVSAPPLGRHYRGVRQRPWGTYAAEIRDPARRGARVWLG

TYETAEEAAVAYDKAAFRMRGSKALLNFPHRIGSNEPEPVRVTAKRRSTEPASESGSPKRR
RKGTAAERAELEVESPSNGLQVGCEMRRLPVGEPLLML

>Actinidia00814.t3

MLDLNLSFARNEDSVTIAEKFPEGSGGPMDESGTSNSSIVNAEDDDSCSTRTSSGENFALN
FDILKVGGEFNNPTSDENGMTQSEFVTQQLFPVTESSAAASLFWPNWVDLSFDQHCPSGV
QEVRVVQQQQGKKSRRGPRSRSSQYRGVTFYRRTGRWESHIWDCGKQVYLGGFDTAHA
AARAYDRAAIKFRGVDADINFNLRDYEEDLKQTKNLTKEEFVHILRRHSTGFLRGSSKYR
GVTLHKCGRWEARMGQFLGKKYIYLGLFDSEVEAARAYDKAAIKCNGREAVTNFEPSAY
EGEMISEVNDGGSDHNLDLNLGISIPSSVDGPKDNESSGRLQFHPYEVHDVRRSNVIVQTI
LSIFPFQANVILSHADIVVVLAFHY

>Actinidia13333.t1

MSTENQNSETESSYNSLSSTPSSPGSTKSPPTRPGDQQDPETSRPKRLRENSKHPVFRGVR
MRNWGKWVSEIREPKKKSRIWLGTFPTPEMAARAHDVAALSIKGSAAVLNFPALAASLPR
PASLSPRDVQAAAAKAAAMAEFDSVTADEFDSSVEASSSSSSSLVSAMELASSAEEELGEI
VELPSLGASYDLAELGSEFVYGDSVDGWLYPPSWLHGGDEQG

>Actinidia07971.t1

MEEPPPPCTEDDLTTASDTASAGTRHPVYRGVRKRRWGKWVSEIREPRKKSRIWLGSFPAP
EMAARAYDVAAYCLKGRKAQLNFPDEVELLPRPSTCAARDIQAAAAMAAKAGVAEKKE
SDGGVDDFWGEIELPELNTRSPPRVFTNFLYKSFMHLPIET

>Actinidia01043.t1

MPCLSETWGDLPLNYDDSEDMVIYGLLNDSVHVGWTSSNLTVTDFAPEPRHETEPSTTTV
HSRFYSPLTFTDSEDGLLRETFRVGLTPCDLTVTDVKVEPMNEIEPATTTSQLVAPPVMVVA
PPKGRHYRGVRRRPWGKYAAEIRDPARSGARVWLGTYETAEEAAVAYDKTAFRMRGSKA
LLNFPHRIGWNEPEPVRVTPKRRSAEPASESVCAQAAEESFLIQTFSVIKIAE

>Actinidia36471.t1

MEGGGKRKSERLYKGIRMRKWGKWVAEIREPNKRSRIWLGSYSTPVAAARAYDTAVYCL
RGANARLNFPDLVAGGGGGDMSAAAIRRKAIEVGSRVDALETSGSVAVGHGHAPSSGFKP
CWFEEKPDLNKKPEPEDPDANDDWFLY

>Actinidia26026.t2

MSNWLGFSLTPHLRVNNEGLEREEEEEEERGGCFSSNVSSVIPLRSDGSLCVMDPFPRSDH
DPQGWRSYDNTTGENTPKLEDFLGCCYPNSPPNDPKNYCQQEDTFINLNYPNQDSINHNP
NISTNPSPFIQACPYNSFQTSDLNPNMNHSMYPYHVPFDGASSVSGFKSWLRQTPYLGHE
QCHGTHTFQSLSLAMNGHAENCGGPVVAPPLEVADGGRKAVGGKATTGRESVPRKSIDTF
GQRTSQYRGVTSDDGIHVFLRHRWTGRYEAHLWDNSCRKEGQSRKGRQVYLGGYDKEE
KAARAYDLAALKYWGPTTHINFPLSTYEKELEEMKNMTRQEFVAMLRRKSSGFSRGASV

YRGVTRHHQHGRWQARIGRVAGNKDLYLGTFSTQEEAAEAYDIAAIKFRGTSAVTNFDIN
RYDVKRICSSSTLIGGDLAKRSSKDSAPTSIEDYNSCTSSALPHPPILSITNDDPSDELVTDTT
ANSEIPSPRSPKGSVLLGDYSQAYFGSQGPQCGDGIDEIRTTRVGNLGLVHHQVPMFALWN
ESRVEL
>Actinidia22649.t1
MESVGCCSSSTSPEKRKQRQNQRDNKPYRGIRMRKWGKWVAEIREPNKRSRIWLGSYSS
PVAAARAYDTAVFCLRGPSARLNFPEYITEEDDLRDGGGADMSSASIRKKATEVGARVDA
LETAHHHGPAESNSARVSVKPDLNEYPDPENSDEEVIGEEFEWVVKLGNNEWNSEGVQG
KNQLSNK
>Actinidia20766.t1
MNGSDGRKRRRRKDCESTEATLLRWKNYRDQFDLTNNGVKSRRKGPGKGSRKGCMEGK
GGPENSGCRFRGVRQRTWGKWVAEIREPVVGRSPQSKGKRLWLGTFPTAKEAALSYDIA
ARIMYGSDAILNFPDKYSKTIDASDKSCSVSTPGSKPISGDSEASVADVEKTCSEVCVDNES
MVKIEDTVRECTGEESMKIEEVTPRQSEVTKAQIAEASCASSVYDCNGEQEEPETADFEVL
GTSGSSECDLRRDKQNN
>Actinidia27956.t1
MHRHSQPTRPAQGGASTSRHPLQPSSLTREEEDSVMVAALSAVISGSTSLPEARQHFLPIILE
PNTCQFCKIDGCVGCNLFPPNPGENYNNNNNNNNNNNSISNHASSSSPSHHNKTVGKRRK
KNKYRGVRQRPWGKWGVEIRDPRRAARVWLGTFDTEEEGARAYDKAAIEFHGPRAKLN
FPFPESLAPPRGARARNWQWREKIRIG
>Actinidia00898.t1
MELHFQMQQKEIFSATKQGTTRKKRASNKSKFVGVRQRPSGKWVAEIKNTTQKIRMWLG
TFDTAEEAARAYDEAAFLLRGSNTRANFVDPGPANSPLSLKIRNLLHQKRTSKQKLTFSPT
TNIATTSKTIRTITEQNRMLDGFEVPKLIGFAPQTNSFETGVDRIPEFEHMKVERQISASLYA
MNGVNEYWENVRDSRDAFWDLPMLCQMFCPS
>Actinidia30276.t1
MGIKVVFVREKARDREDGVRELDGGGQQVQGLGRGFEKVLGGATEGSEPESSSNSLSSTP
SSPSSPGSTQSALIRPGSEQDPEPRKPKRVRENSKHPVFRGVRMRNWGKWVSEIREPKKKS
RIWLGTFPTPEMAARAHDVAELSIKGSAAVLNFPSLAASLPRPASLSPRDVQAAAAKAAAL
PDFESAAVDELDSVAEPGSSSSSSSLVLAMELASSAEELGEIVELPSLGASYDSAELGSEFV
YGDSVDGWLYPPPWFHCGDEQVWGTSDCVIAGCFDSFLWNY
>Actinidia09751.t1
MGRGRAPAKAEAEAIGSSRNGVVEPRYRGVRKRPWGRFAAEIRDPWKKTRVWLGTFDSA
AAAARAYDAAARSLRGPKAKTNFPLLPIPIPDISNFNHNLVEENQFQGFNSGPTTSSLSSTV
ESFSGPRASIPPPAVRIRPRMKPAAPAEDCRSDCDSSSSVIDDCDADLTSSFRKTLLTFDLNLP

PPPEDVGVADADDLHATLGGVTVQGDHASLLYLKLYETTTDRDAILTAFNETTVVARRHP
KLTPESRETISKATVPTTVLLFAVEFDVGRNPKFSDPDNNHVGIKLTNIESVETATAVYYESS
KRRS

>Actinidia14673.t1

MRTENFSNSDPSSPSGSTQFPVDPNPKKIKRIRPDAAAKHPVYRGVRMRNWGKWVSEIRE
PRKKSRIWLGTFPSPEMAARAHDVAALSIKGDAAVLNFPELAGSLPRPASSSPRDVQAAAA
KAAAMDDQGGSSTHAQAEEELCEIVELPSLGVSYDSDELTRDFVYADSVEGWVYPPPWL
QREEDCCGCFSDLTISQSSFGSSGFFFEGLKTLSLSLFLSLTPLCVV

>Actinidia08932.t1

METLKKGYSRLGDLPSATRRRSFQRRLSLAEKPSGHRSHALPTFGTCFKPNTSSAKLLMA
KISQKSHKNSSNTTTTTTATKVKRTRKSVPRDSPPQRSSIYRGVTRHRWTGRYEAHLWDK
NCWNEAQNKKGRQVYLGAYNDEEAAAHHMTWRIEVSTYEEELNEMKGQSREEYIGSLR
RKSNGFSRGVSKYRGVARHHHNGRWEARIGRVFGNKYLYLGTYATQEEAATAYDMAAIE
YRGLNAVTNFDLSRYIQWLRPNQTNPNHSPPNPNPNLDLTPTPTHTDESPPTFLHHQHQTP
TSITAATESTFPSPRPSGRALGLLLQSSKFKEMLERTSAEDYPSPPPPESEPPRSSFPDDIQTFF
DCQDSGGFADGDDITFGDLSSFASHMFQCEFDA

>Actinidia16407.t1

MKSDGNNWLGFSLSPQLNNTNTMEVPQESQHQAHHTPSSSVSISNPIPTSFFHSQPHFSYN
EVFNGDEAENGGFYSPLTVMPLKSDGPLCIMEALNRPQPQGMVTNSTPKLEDFFGGAHH
YEASSDREAMALSIDNSIFYNQNPNHQSFQTVDYSGFRAHEMYQTMGEEETTAMKNWVS
RNYPSNHGLDQKLICSVGENGLGYENLQSLSLSMSPGSQSSCVTGSHQVSATVSECVAKK
RGSEKLEQKQIVHRKSIDTFGQRTSQYRGVTRHRWTGRYEAHLWDNSCKKEGQSRKGRQ
VYLGGYDMEEKAARSYDLAALKYWGPATHINFPLENYQQELEEMKNMTRQEYVAHLRR
KSSGFSRGASIYRGVTRHHQHGRWQARIGRVAGNKDLYLGTFSTQEEAAEAYDIAAIKFR
GVNAVTNFDISRYDVERIMASNTLLAGELARRNKEIEANKEVPNIGHPKQDNVSSETNWK
MTLYQATESDPRPNPSFPMTVGSVQQEGNESGKMANHVSNASSLVTSLGNSREGSPDKQN
SLPMPFAMPSKIFTDSTSNVGSWISSAQIRPPMPMPNVPVFATWTDV

>Actinidia06476.t1

MAEVQLDFLPESSFSWSKLIFLDDSVDPSKEIISSDENFLPHFLAEKVKENPLETDSSSEKK
DLKKEKSYIGVRKRPWGTYASEIRDSTRHGARVWLGTFTTAEEAALAYDQAAFVMRGRL
ARLNFPTDKVRESLREMKCRCENGSSSASALKEINKKRLKILGGKRVSKKERVKQENAQK
VKEKSLETDSSSGKKDLKKEKSYIGVRKRPWGKYASEIRDSTRHGARVWLGTFTTAEEAA
LAYDQAAFVMRGRLARLNFPMDKVCESLREMKCRCENGSSSASASLKEINKKRLKILRG
KNKEENNMLVFEDLGSDLLDELLSSS

>Actinidia14518.t1

MDASNPFFDLFPNRSSFYSTSSIAETSVSPSNNFQSTAEASASTLSESDCTHSEESDIILASSR
QKKRAGRKKFKETRHPVYRGVRRRNTDKWVCEVREPNKKTRIWLGTYPTAEMAARAH
DVAALALKGRSACLNFADSVWRLPVAASTDANDIQRAAASAAAAFRPALEGTVEMAEDE
NRAAENVCFMDEEALFDMGGLALAEKPVAEASDAEGLPVGRSELVFIQRLSIVSAAAVLP
PREISQNQRFESPKKASQEQIDEHFVCEVGVSARVRGGDQRRQVLPFAVLRSANASKQSKP
KSSMLPRSLSRKILQRSFWKRTDKEIERWTSFKYLLEERETPSLFQPDFSPSAMTTTTATAS
NSSINRKSESNSWSDGDFTSDYLPCSSGNSERSVENDVVEGERRLPEKAVSKRIGEIVGDD
SIEATAANSKNKWRIGDEKEQCSPVSVLDVPFEEEEVSSPFSRRLSRMEGTKQKLMQKIRR
FENLSQLKPVGLEDKFSLTEFTDESPESPSPSQLCSDPIQDKIDTKHTAVDLLELFKASFQSS
SFDFRAENLLLDFFRERICKVNIESDYNRLLNEAKDWVDGRAHDISWGGKWKRTGKRILG
IWKREGGGISSALSLPPSDFLLISTRFFIFFWRKLLIGFLSCNHIHVFQNGCAISGIGLNADGV
LYDFFGVDIPGSGCSYAAIFCDPFFLGCLIKISNGCLQVRLGWDVGSISLSYIVLLITVSTLLC
LMIWLAATPLKSASSRLDAQVWNWYINDVPDSSAERHERHYSKTRYGREESTQKQEPSV
ALGNSLESHPNVLLPNSDLNLPETLLDAATIPYMTTTGDNDYNITFPSPTSYLEESSTPVET
GEVSTVCSEGSVSESVETIVLKAESIDMVERSVRIEGIHISRRTKMETLGILKHHLKGYLGV
AHL
>Actinidia17567.t1
MARKRKAGEEAEEKNNPDVEANLAWDEIVKEAAATAAFGGPRRARKRFVGVRQRPSGR
WVAEIKDTIQKIRVWLGTFDTAEEAARAYDEAACLLRGANTRTNFWPCSPSSCLAPALPSK
ITNLLLQRIQARNSSLAASSAPNTYQHKNKKEEFVINELGDFSNTQFTDFLVDPEYYTTPD
NYNMINNSFEEESCEVIEQDFDYNLSDAADSTSGNENVGGAIEEDGEDEEEGIDFGAVDFK
FVDEVGSCYCSPFEIAEEISEPMEQELCGDEPSMLREAMKRMKYERKFSASLYAFNGIPEC
LRLKLGSRGVKARKEEKKTESDSGELKGDKQENELSQTVQVSIPIVNIFSAIIASALNLKIY
KRTSE
>Actinidia38388.t1
MDGRSTDESTTSTDSLSIPAPLEGGVEAESSRKLTSSRFKGVVPQPNGRWGAQIYEKHQRV
WLGTFNEEAEAARAYDVAAHRFRGRDSITNFTSSSERDSDSEKGDDLEAVFLASRTKAEIV
DMLRKHTYNDELDQSRNEHESRGPTNDRAMEATEQLFEKAVTPSDVGKLNRLVIPKQHA
ERHFPLSVSKGLLLNLKDAIGKVWRFRYSYWNSSQSYVLTKGWSRFVKEKNLKAGDVVR
FSRSINHDKQLFIDWKTRGGSGVLNPVRPVQMVRLFGVDIFKVPVSGGVDSSGVNGMEA
GR
>Actinidia27275.t1
MLQKQESRRYIWLLRGSDANVRRLVGAGGGIDKMVRAPPPPSGSSTSGNGHEGVRSRRK
TSSRGYHRFVGVRQRPSGRWVAEIKDSLQKVRLWLGTFDTAEDAARAYDDAARALRGA
NTRTNFELPPQSESNSCRGNYNTSVPENAEPFSFEEVCGTEEADGLLGALKAKLLDGKSNL

RPPLVQVNHLPVQSNFVVACLHNKDNNSTSSTTKGESSSSKPYVNPLMTVDPTRVINVNP
NNNLGNNMTCFRLDHNHTNEQDYMGVVSTGDHQIGMQWQNYSHSTTVSTSIWPREPAA
KEFSPWNSQMNHIHARDLQEDRGLFGRWPLLRHTETTSTDQATVDLSYSRNCCTEDQVS
NSNNRKVGDMTICMPMSVSQNDVMGGVWASDQQIVHCDNNTNWGSGANASWDPLLY
VSSVLG
>Actinidia04217.t1
MEPSMLCPVKHSELRKQTNMVWSSPPVDFSGRTKLPEMSSGGPRVVRITVTDADATESSS
DEEDVGFRHRRVKKFVNEVTIESPSRKNGEITGYRSKTAAKARNRRKRSAGKLKIPAGTFD
TAEEAAMVYDHAAIQLRGPDALTNFTCPQKTAQNSLENPSSTISGYNSGEESHNHDFPSPT
SVLRFDLAPETESSQSQPPNDAVSCCPDFKDDVSVSETFSDFSPVDTIFPDGDFYFEDPVPD
LSEIFDWPGLQENFFDEDCENLFLGSGDVFGYGQTTLPTDDYLRFPDIGDLFGSDPLVAL
>Actinidia38486.t1
MRKWGKWVAEVRQPNSRRRIWLGSYDTAEEAARAYDAAALCLRGPSVPLNFPDYPPEIP
SASDLSPLQIQEAASRHAHRKPQPAESRVTSRVTLVA
>Actinidia23090.t1
MARPQQRYRGVRQRHWGSWVSEIRHPLLKTRIWLGTFETAEDGARAYDEAARLMCGPR
ARTNFPYNPNAPPSTAAKLLSANLTAKLHKCYMASLQISKQSAAETQRVMNSRGGEMGG
GWFPEKKAAAPPQKLEALEEDYIEQMIEELLDYGSIELCSVVPSQDM
>Actinidia24561.t1
MEMLSSSSQAQMLQPTMKIMPFDGASGDSQQCYFLDNFYANGWENKAQEDTAQNIADS
TIFTTFLDSQIQQPPPPKLEDFLGNDSSLVRYSDSQTETQDSSLTHLYDQGGSAYFHDHNDL
KTVPTAAGFQAFSGNSGSEVDDSVSVARTQLGSTESGTELGFSQCPNGALSLGVRARGGV
TRHRWTGRYEAHLWDNSCRREGQARKGRQVYLGGYDKEDKAARAYDLAALKYWGPTA
TTNFPVSSYTKEIEEMKHVTKQEFIASLRRKSSGFSRGASIYRGVTRHHQQGRWQARIGRV
AGNKDLYLGTFATEEEAAEAYDIAAIKFRGVNAVTNFEMNRYDVEAIMKSSLPIGGAAKR
LKLSLEAEQKPSLNHDQPPPQCTTSSSINFSTNIHPLAAVPCGVPLIPPLVTTTISST
>Actinidia15738.t1
MLEIVETINTNAYRLKLPRHILTSNVFNVKHLVPFLGDSSSDEDSNSMTNSFQDGVKDTELI
GYAYMERFDRSAAKRCSNSDPASPSGSTQSPVDPNLTKIKRIQPDAAAKQPVYRGVRMRN
WGKWVSEIREPRKKSRIWLGTFPSPEMAARAHDVAALSIKGDAAVLNFPELAGLLPRPAS
LSPRDVQAAAAKAAAMDGLGGDSRPPQAEEELCEIVELPSLGASYESNESTRDFVYADSV
EAWVYPPPWLQREDDCTGYFTDQTSSQSSFGAVLWDY
>Actinidia12663.t1
MKSPCYNATWAQKGLKRVLQEAKNGKEPRKKHKIGGDCKHPIYHGVRMRNWGKWVSE
IREPRKKSRIWLGTYPTAEMAARAHDVAARAIKGETAHLNFPELAHELPRPATVSPKDIQA

AAAQAAVVTFSSQVDQAEPSRAKLPSSHSSTTLSLENAQDSSNSPSVNDDDTFFDLPDLKS
TDYHASWWQLAGADSSFQLDEPFLWEYY

>Actinidia02153.t1

MALMVCGFEGVKSSCYISSPIMGQVVGLECEEQTTSSTSSTIEAAATPSVSITLKKVQKSD
GEKHPIYRGVRKRSWGKWVSEIREPRKKSRIWLGSFVTPEMAARAHDVAAIAIKGQSAHL
NFPDLVHELPRPASKSPKDIQAAAAKAAVLDASRSHGAEAEPSQAEWTSSQSLDSSTGGSS
TSPIRQTDDPFFDLPDLFLNLGHCTDDLHYMLTWQLTGDEPFGGEFWPDQDPFLWG

>Actinidia35862.t1

MENQFLTMEKELLSYLQVMSMGSRVCLDAANFSGIADETLCHGGSSFKSSLSSPDSFFSSL
ESNSPTETCTSPYTNALGGYIPLNFLKSFDQSSPPSSSSPSKSPNLGQSHLQEGIEWIKINQST
MNCASKGFSDYWLSTTKTQPMKYTGRRMQPPNHQKASFPSTSSCERKLFRGVRQRHWG
KWVAEIRLPRNRTRVWLGTFDTAKEAAFAYDTAAYILRGDYAHLNFPDLKHQLKASSTNC
ATKALLEAKLQAMSGNKKDKDPQATSPKKNHFKGLNRIEPTRREWDFDLESKVGSEMV
MIENKKSQEVLPDVEVVQLSRIPSLDMDIIWDALLVPDS

>Actinidia38068.t1

MGEGKNKHREHKEKSPYRGIRMRKWGKWVAEIREPNKRSRIWLGSYSSPVAAARAYDTA
VFHLRGPSAPLNFPEYIAGEEEVRDISAATIRKKAAAVGARVDAIETAQYHVPSEGNSGRVS
EKPDLNEYPNPEKSDGN

>Actinidia02048.t1

MAAASAVETFRPLPEGGVEVAQDAAENVCFMDEEVSFDMRGFALDMAELPLHSPPQRSS
FYSTGSTPQSENFQLTDSAAEAPSSTLSESDGTHSDESLVVLASSCPKKRAGRKKFRETRHP
VYRGVRKRNTDKWVCEVREPNKKTRIWLGTYPTAEMAACARRGGAGPQGPVGVPELCR
LGVAASGASFHGCQRHPDGGSRSGRGVSAGAGRWR

>Actinidia31286.t1

MLYDSGMAVGMGEGGRLDGFECRHWQHRSDTVLCQSDLGSARVSGKHPVYRGVRRRK
SGGKWVSEIREPRSPNRIWLGTFPTPEMAAVAYDVAALALRGSEAELNFPNSASSLPVPAST
AARDIQMAAASAAAAAGAAVDAFSGSRNDDQDKYGTSQQSEDPIRYNEFVDEDLIFDMP
NVLVNMAQGMLLSPPRYDFADEDTGDENTEDQNLWKFS

>Actinidia29390.t1

METDMSLKQTYRGVRKRKWGKWVSEIREPGKKTRIWLGSYETPEMAAAAYDVAALHLK
GICGARLNFPELAGSLPKPASSNAEDVQVAAQEAAMRFRKGPLTEVGGGGGGSGDMVVP
VTVQLSPSQIQAINESPLDSPNMWMEMAGVLGYRYTRFDGCSNTSEEVSSSAWEIVNSFR
MANARYEAAWFRRVDYSIAHRTGNRVVHQLARNALCFDDPVIYIKHSIQNEMRLMMCN
HAMDENKMGKDLSTNVLIAKQVISQ

>Actinidia24986.t1

MKHKPTTSSSSATAAAVTATTTTTATSRSYRGVRLRSWGKWVSEIRLPRQKSRIWLGSYPT
AEMAARAHDVAALALRGQSATLNFPQLAHKLPCPATAAPKDIQAAAALAAAGEFDGFEIE
AQAKITSATSSCEAQELGRSLASESSDDDDDALFDLPDLLVDSKMDSSNGFCFASWFNGR
QSRLQCPTWSWESGEPSKRKSYLPSEKQFLITRNVPCLRRAASVEEEYEAAGGEVLLDND
DNDGWLATHGKPKGNKCDEEENLPSMETLEISKNNTIQSISSYFGGEEEEDIPDMAEYEEP
DNLIETDPATLQSTYLVAHEPDDDNILRTRTYDVSITYDKYYQTPRVWLTGYDESRMLLQR
ELVLEDVSQDHARKTVTIEDHPHLPGKHASVHPCRHGAVMKKIIDVLMSRGVEPEVDKIT
HLVNLKTVGWVRTCKSSVITQNGCPRSTTDDEVGKKDCVYTLYIQTGWGITAGTNSKISA
TLGDSMGRSVWAPDLEDWANGLGTTTTSSGPSDIFGRRGPCFRPLCRLTSLRRVRAHPTG
WFCGVRRSPPLGPIHVAQTVVFVQSKWLVSNAPPYEMTAVIDGCKGKGGPPRHGNTGPLV
VGKPIGSASGIIWVLGAP
>Actinidia02176.t1
MATPDEVSALELIRQHLFGEVSPVKSFISDVTNTGSTLTAHDYTIDLKTPDSLNSITESSPVS
RSSNRKRSLKIELAQVNKFEWLNFEEPTKSAVPEEKSAVAEERRHYRGVRHRPWGKFGAE
IRDPKRRGSRVWLGTFDTAIEAAKAYDRAAFKMRGSKAILNFHLEARKLNHTAVAAANGS
AKSVDAGGGTPHAHSSSSPHLDFSLMPFSNPLPVIYSKSSDVFHTFSYSSLATLLGEIGDAK
WLLVDESETHFLILISPIIHCHIGTEAGQLQWKWTRQQFPLKPSAKKLLIWRTFLLKKFLKS
WLVQVRVSVQMRFKSVWMCSDTTNLKRKRKVRYLSF
>Actinidia17188.t1
MSDRSSPQYLRFNFLPKSSLSLEENNSSDDTCLPHFQSEKVKEKCLETDSSSGTKDLKKEK
RYIGVRKRPWGKYAAEIRDSTRHGARVWLGTFTTAEEAALAYDQAAFVMRGRLARLNFS
TDKVCESLREMKCKVCESLRKMKCRCENGSSSSKALKEINKKGLKILRDPSLALPPAVGD
WDASSGRSRATNSSMAEENNTSDESFLPHFLAEKVKEKSLETDSSVGKKDPEKEKRYIGV
RKRPWGKYAAEIRDSTRHGARVWLGTFTTAEEAALAYDQAAFVMRGRLARLNFPTDKV
RESLREIKCRCETGSWTANSLKEINKKRLKILRGKRDSKKEGVKQENDNMLVLEDLGADL
LDELLTSSLSTSRVGT
>Actinidia20414.t1
MSSRETESSSIHSDSSPSSSSGIQPKSPVLVQNRPKRSRQESNNTKHPIYRGVRMRAWGKW
VSEIREPKKKSRIWLGTFSTPEMAARAHDAAAITVKGGSAVLNFPHLADLLPRPATCSASD
VQAAAAKAASMDHLSPSAATTSSSSSSSETAEDELSTAATAELGEIVELPSLGESYDSAVRS
DSVVDSRALFPAISVPPRDYSFGLQSRRLKSRGPVFTTVLLISGQPN
>Actinidia35366.t1
MACGLRARAMATGEADGSGLAKEIRFRGVRKRPWGRFAAEIRDPWKKARVWLGTFDSA
EDAARAYDAAARNLRGAKARTNFPLSSILAPHSPFDQNPNDPFIERRLYPPHHQIIPQRPTS
SSLSSTVESFSGPKPPRPAVAPPSSSRRRPRTPPVSPDDCRSDCDSSSSVVDDGDCDIASSSLC

KALDFDLNLPPPLDEADDLFCTDLRL

>Actinidia14855.t1

MESCCIDESTTSDLLSFSPARTPPATVKSPPESLCRMGSGTSVVLDAEGGHRAEEIYEKHQR

VWLGTFNEEEEAARAYDVAAQRFRGRDAVTNFKPLSENETGDEAEFAFLSAHSKAEIVDM

LRKHTYGDELEQSRRSFGGKKQCKEKISNPSGSDKTIGKATQQLFEKTVTPSDVGKLNRL

VIPKQHAERHFPLQSGSTSKGVLLNFKDVVGKVWRFRYSYWNSSQSYVLTKGWSRFVKE

KNLKAGDVVSFHRSTTVHDKQLFIDWKVRGDGPGVAAFPVQVQPVHMVRLFGVNIFKVP

VSGGVVESSGGCGGNKRIREKEMLGFGCSKKQMVIGAL

>Actinidia09838.t1

MIPAGEPAYTYIPTRETSETYEGEPMRKYRGVRKRPWGKWAAEIRDPHKASRVWLGTFDT

AEAAARALNFRGNKAKLNFPESVTLRPPPPPPSSPATHSPFSHPPDTLFPISSSSYPIVHSQYL

QSYMNMNYLPQPSNFPLFFPVQSPVGGGSDGDFLGTTSSDSSHQPSSSG

>Actinidia28438.t1

MMSGYRREAEMSAMVSALTHVICGEKRAREEEVVNGGGLSESVSRVSIACGDFSFGGGG

SSSSCPKEGSTMIPAAEPVYTYIPTYETSESCEGEPMRKYRGVRKRPWGKWAAEIRDPHKA

SRVWLGTFDTAEAAARAYDEAALNFRGNKAKLNFPENVTLRPPPLPSLVTHLPFSHPPNTL

FAVSSSSDPIVHSQHLQRYMNVNYFNPHVFGNSSNFQSSNSSIGYTVSSLSSSSSSSQPSNFP

LFFPVQPPVDGGSDGDFVRTTWSDSNHQPSSG

>Actinidia40245.t1

MSTSQTPQKPSQPYDPARPPASPFLHMTPPRPSGERRGRRKQAEPGRFLGVRRRPWGRYA

AEIRDPTTKERHWLGTFDTAHEAALAYDRAALSMKGTQARTNFVYSHQHNNNSNNNTLT

PFDLQTLIQPSQYSHEQFFITTNQKRPCQPETTCQGGTHIVNHSDNDSGSQSSYGSSPNDSN

NFFSSGQNNSGYLGCIVPDSCLNPPNTTKSSKDGNLSSYTSSQTHFDDMATTDNNNPLGV

ENGCMEMENLYPNSGNPMPTASSSSSATCPYFVPPFGDGVEFGYSLF

>Actinidia20249.t1

MMKLVSFVMEEPPPPCTEDDPATSTDNRRKSASVAGTRHPVYRGVRKRRWGKWVSEIRE

PRKKSRIWLGSFPAPEMAARAYDVAAYCLKGCKAQLNFPEEVELLPKPSTCAARDIQTAA

AMAAKAVVAEKKGSASESDGGHDDFWGEIELPELNAFSYECLVWDDAKIISYGLT

>Actinidia08910.t1

MHGKRPLPSDEPEKKEEEENIFSLYSARSKHDMTAMVSALTQVITNTTNHDKATTPLDDLL

PHNPLALPRSSAPEPSLSQPGQDQANQKRRHYRGVRQRPWGKWAAEIRDPNKAARVWL

GTFDTAEGAAIAYDEAALRFRGNKAKLNFPERVQGRTEFFHLTNRADSRVVTNRVPDSFPG

PPPPHHQIRSQEPYPNLHHYAQPLYSGGNDGITAFRVQTLEEI

>Actinidia18633.t1

MCGGAIISDFVPNYCSRKLTTRALWSELDPFSDFSGLHHHGIENRSNKSNNKTASPNPKQI

NKGTSERARKNKYRGIRQRPWGKWAAEIRDPQKGVRVWLGTYNSAEEAAHAYDAAATR
IRGGKAKLNFPPQPQPAKKRCVVPESTRVTQLPTESTQFAYYPDPVADDGAEFEERISDLES
FLGLDSEPTQFRGLGVPDSVDLWMFDDLPVTHLLN
>Actinidia15778.t1
MVQSKKFRGVRQRHWGSWVSEIRHPLLKRRVWLGTFETAEDAARAYDEAAVLMCGRNA
KTNFPIATEASDNAETSSTPTSSSVALSAKLRKCCKSPSPSLTCLRLDPDNSHIGVWQKRAG
TRPDSKWVMTVQLGKKNEESPEKEVPAATAEVGDGLGEEERMALQMIEELLNKN
>Actinidia22149.t1
MGKDFPVKFTNKIARMQENNNPFAFTGIAGNRSSLSDLILAGGTNTLDSIFSHCSPSIPVPN
PVVLEPLGSSVYLQQRDLLQKFSKETPSNTAISQISLTSPLQNSLYTNSYLTQHKKKLYRGV
RQRHWGKWVAEIRLPQNRMRVWLGTYETAEAAAYAYDRAAYKLRGEYARLNFPNLRDP
TKLGFGDCTRLNALKSAVDAKIQAICQKIKREKVNKSGKKIESSKEDSGKEIKVDSSSLGG
SESWSDQVQSSAISDDGLRKGENSPASVSGEWLMTAESSELDCCSLAHMPSFDPELIWEV
LAN
>Actinidia25518.t1
MNQSEKKEEERNNIFPAYSARSKHDFKAMVSALSQVIGSNSKNPVHLPEPNQSHLPSQDQ
GNQKRRHYRGVRHRPWGKWAAEIRDPKKAARVWLGTFDTAEGAALAYDEAALRFRGH
KAKLNFPERVQARTDFGYLRNHQKKNYPNFLHHAQLLHGKNNGVWGIDPRETFVSQPLS
SISSASSSTTSQQQEGEVMRFGETILVPKTQEGDRFA
>Actinidia39896.t1
MLDPDDIIEFHLDDKSMDEHHKGKDKHEKGREEVRYRGVRRRPWGKYAAEIRDTSRNG
ARLWLGTFDTAVDAARAYDRAAFNMRGHFAILNFPNEYYPQLSNSPCPLPSASWGSTLAP
SEGFDHKVGPSTWQEREVIEFEYLDDKVLEELLESEEEKQKRMKG
>Actinidia34076.t1
MDVFSHFSDPIHGGSAYWDGDLLESSSPASDGGSGRPADWSDEERVMLASSHPKKRAGR
KKFRETRHPVYRGVRRRNSGKWVCEVREPNKKSRIWLGTFPTAEMAARAHDVAAIALRG
RTACLNFADSAWRLPVPASPDPKDIQKAAAEAAEAFRPSESEVEVISGSGGAGPEASEELPE
RMIFMDDEAVFGMPGLLTNMAEGLMLPPPHSVGGEAEMISGGGGAGLEAAEELPERVIF
MDEEGVFGMPGLLANMAEGLMLPPPHSVGGGWDDDVEFGTDVSLWSYSI
>Actinidia22309.t1
MARPQQRYRGVRQRHWGSWVSEIRHPILKTRIWLGTFETAEDAARAYDEAARLMCGLRA
QTNFAYNPNWSQSSSSKLLSANLTAKLHKCYVASLQLTKQMEKEPHKVLGPQVVAHNSPP
AVQKANWVPQFRPLEEDHVEEMIEELLDCGSIEICPNVPPHGV
>Actinidia02178.t1
MKAYSSGSHQPKRSSTFEWRDGSSACERFENFCGCFIMVTPDEVSALEVIRQHLFGEFSPV

AELRHNTSMFTTHDGTVSVTTDNSSSQSHSFCSQTASSDGHNFNERNGAEIFDCVSDTINF
DCNQNDLFEFESKSQIIDLTTPKARNDTENFGCVSDSIHFERNRNDMFELEAKPVIVDLMT
SKSMNSSTQSSSVSSFRNRKRPLKIDLAPVKKIEWLNFSEPKVSEEKPSNTEEKRHYRGVR
QRPWGKFAAEIRDPKRRGSRVWLGTFDTSIEAAKAYDRAAFKMRGSKAILNFPLEAGTLS
KTAVASAVNGGAKRCRDVEVKIRAEWQVKQGMVR
>Actinidia02046.t1
MAWDLPPAEMARVRTTWQAFLALKGSSACLNFADSVWLLFAVPASTDSNDIRRGDYSGP
GWFSPPPEGSIEAETSVCDPAAEASSSPLFESDSTNSDESDIILASSRPKKKAGRKKFKETRH
PVYRGVRRRNTDKWVCEVREPNKKTRIWLGTYPTAEMAARAHDVAALALKGRSACLNF
ADSVWRLPVPASRVQRHPEGSDCGGRGVSSGTGRQH
>Actinidia04147.t1
MDSSQNWLGFSLSNHHMPSDSSHLCLFQAFNANHPSGGVDRVSEDHQDPSSGPITDLSIFT
GGPKLENFLGGGGAEDVCQFTGTTPATNVSQTEIYDSELKTIAASFLRAFSTEQADGQKM
LAVPSKKPVDTFGQRTSIYRGVTRHRWTGRFEAHLWDNSCRREGQSRKGRQGGYDKEEK
AGRAYDLAALKYWGPNTTTNFQVSNYEQELEEMKNMTRQEFVASLRRKSSGFSRGASIY
RGVTRHHQHGRWQARIGRVAGNKDLYLGTFSTQEEAAEAYDIAAIKFRGQNAVTNFDMS
RYDVKSIASSNLPIGGITENSKTSSNSASDKNSIDGVKLDHRYLSSASSSVTFALQPPTSSLS
LGLPIKQDTTDNYWSILGYHNNPSLNSTPENQASSAAPLFQAPTSGGTSSFQLGTAPYSMD
LSTNNNINNNNGLLNGGTVYLQQQSIGNSSGIAFATPIALSSMLSCGGKSRGEGRKG
>Actinidia15373.t1
MLKSVMGMGEKNKSAKKTLRATSRKGCMRGKGGPENAACTYKGVRQRTWGKWVAEIR
EPNGGDRLWLGTFDTSREAAIAYDAAARKLYGPEAKLNLPELRAAVHTQQSPSVSLLCDS
TSGAHTSESVGENDIGIDAIRENLNANLPDFDDSSMWAEATASMDFQAMTDPGIFFEDGM
GPWCYCSTLPESHAFCRIQAKESSRAKTETKSPAKQEGKQSDLHVSPLEQGHVALHTLEK
LLGACDGGFTKCSTRS
>Actinidia35241.t1
MAPRDKPISINGSNGAAVNAKETRYRGVRKRPWGRYAAEIRDPGKKSRVWLGTFDTAEE
AARAYDAAAREFRGAKAKTNFPTASELIATANHVQSVTRSPSQSSTIESSSYVPPITAVSLPQ
RSLDLNLAYIGGGHFSGFIPTARPIFFEALSRSDALNNHLNLYRFDRKVADLRPTGAGGVQS
DSDSSSVVDNIHCKRLDLDLNLPPPPEEKSALVSESRERTQPRDESMC
>Actinidia03102.t1
MANPSNVRQSDPKLTSESDPSRPSLSIVRPDDYLPPNRPNMSPLGLSSDPSSVPNIFPFTISCE
ESSPKHPQSPTTAASPSPRHRIFRGVRSRSGKWVSEIREPGKATRIWLGTHPTPEAAAAAYD
VAALALKGPDAALNFPDSILSYPVPASSAACDVRAAAAAAAAARHSKPVSAGESSEHVRV
ENEEKGEGETTSGEEFIDEEAIFDMPNMLVDMAEGMLLSPPRMKSPPCDDDSAESSASLSL

ECSRI

>Actinidia40060.t1

MVVEVEGSDNGGKGYGSNGNGGELGEVVVVVVEVDVEMVATMGILVEVDEAERTHVSF

TKKKKKFKMDLNSSVFHSQYSDFSPESSLGSPLESFSWNGLNFINSLPFNENDSEEMLLLG

VLAEAALETSENVSSDPSKEYEVSSKPEGKPTKEKSYRGVRRRPWGKFAAEIRDSTRNGIR

VWLGTFDSAEAAALAYDQAAFAMRGSSAILNFPAERVRESLCEMKYNFAEGGSPVVALK

MRHSMRRRLGRKSRERDVGRDVCAEDLVDTWKSF

>Actinidia07597.t1

MAPKAAATAVKGIVKEMHYRGVRKRPWGRYAAEIRDPGRKSRVWLGTFDTAEEAARAY

DAAAREFRGAKAKLNFPLLDENSPSPSSTVESSSPVAGSPPPLDLSLSFGGGARFPLQQYPF

ASPVTGVMPLANHVFYFDSILRSARMNQDYLRDQRLRMDHVSHVKAVTAVDFRGGGGA

QSDSDSSSVIDLNLSDVKTRRAIDLDLNLAPPPEY

>Actinidia31772.t1

MTTDESFALELIRQHLLGDFTSTGSFISNLSFPASSNGSDPKPVFSFEESDLSPSGSSDSNSPIS

RPNQYPSPNSDLDYPNPDFNFFEFESIPPEIFDVKATRPTAFSSSDLEFFEFDSKPEIADLILPG

TKTFSGSDLYISETLNPEIADVISPGPNIFNGSGAVGTGSGQTKRHYRGVRRRPWGKYAAEI

RDRNRGGSRVWLGTYDTPIEAARAYDCAAFEMRGRKAVLNFPLDAGKYGPPESAGRKRR

KDSGAKRN

>Actinidia26237.t1

MARKRKAGGEAEEKNNPDVEANLAWDEMVKEASTTVALGGPRRARNRFVGVRQRPSG

RWVAEIKDTIQKIRVWLGTFDTAEEAAMAYDEAACLLRGANTRTNFWPCSPSSCSAPALPS

KITNLLLQRLQARNSSLVASSARNIYQHKNNKKAEFIIDELADFSNTQLTDFLVDPEYYPIP

DNYNTITNSFMKESCEVREQDFDNNLSDAADSTSGDGNFGGVIEEDGEVEEEGTDLGVID

FKFVDEVGSCYCSPFEIAEEISEPMEQEPCGDEPSMLREAMKRMKYERKFSASLYAFNGIP

ECLRLKLRGVKARKEEKNMESDSGELKGDKQEENELGGSSEMGSIDDVHEIT

>Actinidia11342.t1

MLSKIVHVQPMEKEMAGEVKYRGVRRRPWGKFAAEIRDSARHGARVWLGTFNTAEEAA

RAYDRAAYAMRGHVAILNFPEEYQLPSSTCGGSSSSSSSSMCTSREKKQVFEFEYLDDKLL

EELLDSEDKKTRRQ

>Actinidia14109.t1

MARPQQRYRGVRQRHWGSWVSEIRHPLLKTRIWLGTFETAEDAARAYDEAARLMCGPR

ARTNFPYNPNAPPSAASKLLSANLTAKLHKCYMASLQISKQSAAETQRVMNSRGGEMGG

GWSPEKKVAAGAGAAPPPKLEALEDDHIEQMIEELLDYGSIELCSVVPSQDM

>Actinidia09595.t1

MDLINSSNKVNSPSSSSSTSSSSKSKRKNQQQSQKDLNDSGGLRFIGVRRRPWGRYAAEIR

DPLTKERHWLGTFDTAEEAALAYDRAARSMHMRSKLAKPTPPPPPTGHRPVGFFHFSGDE
WIQRQYTAEIITITASSTNNSFVMSSRLLQDCFDYAWRCWWGGVDQDQPHYYCNVDEVE
AELPPLPPDISNSYDSRSMCGANVHGSVWNEEDPMSNLLIDNGRFSDQQTTTGYCYHEAE
TGKSTVGIESYNNVNVGFLMESASSSELNDPLSDDTVSF
>Actinidia35367.t1
MGRARAMAAGEADGSGPAKEVRFRGVRKRPWRRFAAEIRDPWKKARVWLGTFDSAED
AARAYDAAARSLRGAKAKTNFPLSSSILAPLDPHSPFDQNPNDSFIERRLYPPHHQIIPQRPT
SSSLSSTVESFSGPRPPRPVVAPPSSSRRPPRTPPVSPDDCRSDCDSSSSVVDDCDGEIASSSF
RKPLDFDLNLPPPLDHTLIGFIVRTIDETYLAGKGEKTRKLIYLWEEQCRLVFDKVIMGWG
LEEVS
>Actinidia39827.t1
MTFVFGVSLSLPLSKFCLDLLIGASKQPSLALFPITLGLNPPRQPKPEHSYAKELLFWSGLL
WGCSYLLFNLFGLRFVLAYWSLVDENMTNKRVKKENTGEEEDHVTSYRKVRIIYNDPYA
TDSSGNDDDESIYDYNNGFTRMKNGFTRVKQCVTEIVVPRLPCAGNYLRNCSNGGTVGT
KKNCDRKRTQRSLSMYKGVRMRKWGSYAAEIRDPIQKRRLWLGTYKNAEEASKVYESK
RLEFEKALLSVKNKNSNFNVEHPCALEDNSLYSPSSVLEVEDNSITSGKEDDGLVSIVEEEQ
LIARFLSDQFVLPPVFEDMNLLRINDLAQQQTLEFLGDSKHRRDFNLRFENNLSYDKNMG
QFSDGLDDVTIDFSTCGENYDLPNFDFELDTEELAWVNEALNIGCPSTNLFQVFK
>Actinidia25891.t1
MKMEEIGARVLPVRKSLAPPLLLLLDRDVVAQADFDADLALLESITRHLLTESELPAARCL
GTDYWGDLPLKENDSEDMLVYNILNGGWLMPPPPENTACNLAGTGSFPAAAPPKTEAPE
VMPAVVPSKGKHYRGVRRRPWGKFAAEIRDPAKGGARVWLGTFETAEDAALAYDRAAY
RMRGSRALLNFPLRINSGEPEPVRVRSKRASPEQSLSSSSSASSEDGSPKRMRKAVVAAEP
VVVQPKLEAVEAEFDFAHILDDSMWLEIIGSGC
>Actinidia17778.t1
MNSPPLLLSPLSTLYLPTPTSFLSAAACTSDTSNTSITSQNIGVNTKQSRRNKSRFWRAKSK
SELSILLYSPRPTISPKGSSKHAGNSPGLPASDITDGADPPLIYARPRETSAMVRALTRVVSG
RRLDETAAFGSGSGVNYSPDSADFSAYDHSISGLKREREEESGTQYSEDRIQRVYGGFSDV
RDIHGESSSVRTAAEEPTNIMIPNTTATATTQQPPPPDTTTPQEDQPGERRRRYRGVRQRPW
GKWAAEIRDPHKAVRVWLGTFDTAEAAARAYDEAALRFRGNRAKLNFPENARLLPTVVP
PPPAPHFRAAHPPPTAATPLPPQRAIFQSQQFYSPEIVRDYWNYSQLLQNPATSFSSSSSSSS
SSSYPLFFSDPQSGFFQPPESQNQGGGSDFPENSWTGPGQYPPSSR
>Actinidia33496.t1
MEEQINIESLAQDYLPSSPTTTTSTSTSFSSISDGLKSCKAQKGLKRVLQEAKNGNETRKRH
KIAGDSEMAARAHDVAARAIKGESAHLNFPELAHKLPRPATASPKDVQAAAAQAAAATFS

SQADKVKPSRAKLLSSAPLPLCH

>Actinidia13662.t1

MSEPPPNQRNIFKKPKKKPVCAEENTRKMKKIRLSFSDPYATESSDDERSGFEPKRIVREIN

LPVGVLNLPKPPESESSCQDSNNGETPPHRKRVSPKFPSSTIPGVRQRKRDRWAAETRDPV

SGKRLWLGTFNSPEEASKAIERKKLEFAAMAGDKSCYNSFSMAVSEYSETSSHSSPPSVLE

SDSVASQSDAKYSETVKDVGVDTNLGEKKVIDSVELDFGMELDSLFLGDFDDVLGDFFGL

DDLQFCGFEDGEQSDLPDFDFELGSEELAWIGEAINIAYP

>Actinidia00960.t1

MASSGHHHPTQDKPIPLPTAKTTTTPNTRRCKGKGGPENSMFKYRGVRQRSWGKWVAEI

REPRKRRRRWLGTFSTAKDAAAAYDRAAVILYGQRAQLNLQPSPAPISSGGGGGSSSARG

GGSSSSSTQTLRPLLPRPPNFSVTFPPYSSTLPSPIVIFTTLFNSLPPYKVRASFRRSYLPSHHP

NYLYEQINSLVGSVDSSLCLSSHHVVEPAVSDPTVAAVETSAVGSPVIWPVTSDDEYPPASI

WDYGDPSFDF

>Actinidia05668.t1

MVKSFIWEASLWRQDPDHGPLGFYNSYHTAVTTVFGPYLTITREEKELVLEDIMDNLKLLI

LLRCKDKRVQFRTTILIQDLCPAQFGQEGFIWELKLNDWSEIEKGAVVEDLGTAVTPCDVE

FALENATSGFYDEMLSFALQIVTLGFLDEMLLFPLGIDATLGFHVALACTPTLPLESQTVTQ

NVLKPIPTPGKKNYRGVRRRPWGKFAAEIRDSTRRGARVWLGTFSTEEEAALAYDRAAFK

MRGAKALLNFPTEVVAASTVKGYHWNLSSEKLDQHDSNSSAS

>Actinidia22349.t1

MVVQSKKFRGVRQRQWGSWVSEIRHPLLKRRIWLGTFDTAEAAARAYDEAAIVMNGQN

AKTNFSVPTSTDTDKDIFTKDTPTMADILTAKLQKCCKDPSPSLTCLRLDTENSRLGIWQK

RAGPRSGSSWVTRLQLGKREKMNYRPSESSSATCPVEMGEEIEEENRVAMQMIEELLNGN

SGELVPL

>Actinidia11051.t1

MASPRKSGKSKKGIEESQKMTQMEWEREHEMGLEARQWSPVFDGASMSTRPLKKVRSP

DRQRPFPSSSSSSQPPPLSLSLPSSVPQTTTTTPTALNPFQSTSRHVFPFALDGSEQQTMEIPQ

QFRTNPPLLIHPQPQNHQQMISFSPNHHGIAYPPNFSGDLASLQQQQLLQYWSDALNLSPR

GRMMMMNRLGQGSGGGLFRPPMVVPPISTTKLYRGVRQRHWGKWVAEIRLPRNRTRLW

LGTFDTAEDAAMAYDREAFKLRGENARLNFPERFLNKNKTDPTIDDGSASQGLNLQDSGT

EILPPPVPLSLPQQESPDEDSGKGSSEATKSGEIPGFAGEGGSESSELVWGDMSEAWLNAIP

AGWGPGSPVWDDLDTSNNLLLPSNLPFANRIDLLKHLRSLAQPVTVTSGSLKVCLTVNFR

TPY

>Actinidia06105.t1

MARKRKAGEGAAAEENPNEENLGWDEMMGEDAAAAALGGPLKTRKKFVGVRQRPSGR

WVAEIKDTIQKIRVWLGTFDTAEEAARAYDEAACLLRGANTRTNFWPCSPLSTLTPALPSK
VTKLLLHRLNARNNSLAASSTSSYVSINQQQQQEEEYIDEVSDFSDSQFTDFLNDHEDYVT
TDNSMIIDNNASFGESMTRSFESCLIEQTSCGVREQDFDYNCSGLGQSYSDDANPGGEIEE
DGEEEGTDLGVVDFQFVDEVGSCYYSPFEIAEEISEPMEQEFCGDEPSMLTEAMKRMKYE
RKYSASLYAFNGIPECLRLKLGSGSTVKQRERSDLLTHLWNACNENKEEKKIESNALGDK
EEKIEQSSSDMGFSMSNEGSRFRTTAL
>Actinidia11629.t1
MTLGEIMNVLRSKIVICLLNIIMAEPFILYRALFVFLRVAEHDRDLLIEYNYGRAGLVRKDG
LTVHNRRSMTSGDSSMAFRRRVVRIILTDADATDSSGDDDKQAVRRVKRLVKEININQSPN
KLEQSKKRKFRSLGSAGSDAVRRKKFRGVRRRPWGRWAAEIRDPTRRKRVWLGTYDTPE
EAATVYDRAAVKLKGPDAVTNFPNVSVTEDAVDVKTDGEGSANVAALSPTSVLCYDDLT
PFDGFSYGDVDAFGFDIDKPLSLADFAVPENFYGADDLGEEFDLDDFLVDVR
>Actinidia37989.t1
MGVLTISVSANGLDAPTISLIEMQLSIEGKDKALTTNVMRDENDKVLVFGQQGEEVSLELI
LRVVADVGLIGLPNAGKSTLLAATTLAKPDIADYPFTTLMPNLGRLDGDPSLGAGKYFSEA
TLADLPGLIEGAHLGKGLGRNFLRHLRRTRLLVHVVDAAAENPVGDYRTVRELISRIGRD
ETPSQSDTSSGYAIEALPGEDDHANVFSSGISDADGNIKEIEDYPRPFAVVGASVLHRLLAT
TECWIENIVPHHRPQEREAASSSSRQRTHHCNLKKRLYERERWPRECLLHLQGCPVGEIRK
PNSGARLWLGTFDTAHEAAVAYDTAACKLYAAWMPNQLAGLGRGQTQHLRVGENDVGI
DGFWGNLNANLPELNDSPMWREATATMNFQAMADQGIFAGNFKDGKGWEGIQYPWCY
>Actinidia39895.t1
MEKEVAGEVKYRGVRRRPWGKFAAEIRDSSRHGVRVWLGTFNTGEEAARAYDQAAYAM
RGHLAILNFPEEYHLPSSTCGGSSSSSICTSREKKQVFEFEYLDDKVLFHMRHTLKEQSFSR
KS
>Actinidia19931.t1
MVRKRKVEGEEVKENNFGNMGWDQMMNEAAANAAALGEVQRTRKRYVGVRQRPSGR
WVAEIKDTIQKIRVWLGTFDTAEEAARAYDEAACLLRGANTRRNFWPSFPSPNSPPALPSRI
TSLLLHRLKARNNASPTMPNSSDANSQQSQAAGENEDQIGGEVEVTMEGEEENDVGIMD
FDFVDNVESSSCYYSPFDIVEEMAEPVEKENYGEEELPSMVTEAMKRMKYERTFSASLYT
FNGISECLKLKLGSRNLSKLRHNSSEDQEKTNKEEKHFVQEQGRSQQNSTETGSSTSFTTT
SSSHSSSSSMEGGEWSLWSSLDLPPIAMLFNGLNGS
>Actinidia19832.t1
MGRSSWRRQSRRPRSPSIPSVNAPLFTEESPGIGGLVDMKLICGITVAEGKGKVGKEDKVY
MGGYDKEEKAARAYDLAALKYWGPTTTTNFPVSNYENELEEMKNMTRQEFVASLRRKS
SGFSRGASIYRGVTRHHQHGRWQARIGRVAGNKDLYLGTFSTQEEAAEAYDIAAIKFRGL

NAVTNFDMSRYDVKSIASCNLPVGGITGKSKTSSDSALASDKNSKDGGQSDDRDLSSAAA
SVTFAPQPPSSSLSFGLPIKQDTTDNYWSILGYHNNPSLNSTAKNPATASSATPLFQASTSGG
GTTSFQGTTPYSMDLSTTNNINNNGLLNGWVYLQQQSIGTSDGIPFATPIALSSNGNSYKG
GSFGNWVHSFQSAKPNLSVFQTPIFGME
>Actinidia29985.t1
MKSVVGMVVSNKMQKSVVVAVDRLFHHKLYDRYVKRTSKFMAHDEHNLCNIGDRVRPT
LTLQKGRQRRGKGPYAGVRSRGGRWVSEIRIPKTKTRIWLGSHHSPEKAARAYDAALYCL
KGEHGSFNFPNNRRPYLANRSVGSLPVDEIQFIAAEFSCFDDSAATSEPFPMSPLMETHLSS
EPPVTPDPQNVNEMEANNVDAEPYFPAFMQEEEPHVPTFVPEEPYIPAYVREAAPILHGDL
LLDEWLTLDGDWMRNFH
>Actinidia02177.t1
MATPDEVSALELIRQHLFGEFSPVTDLSHTTSIFTTHDGTVSVTTKNSSSTSDSFCSQTASSD
GHNFNERNDAQIFDCVSNSINFDCNQNDLFEFESKYRIMDFTTPKELNDTEIFDFVSDSIHF
ERNQNDMFELEAKPVIVDLTTSKSVNSSTPSSFSNRKRSLKIDLAPVKKIEWLNFSEAKVSE
EKPSNTEERRHYRGVRHRPWGKFAAEIRDPKRRGSRIWLGTFDTSIEAAKAYDRAAFKMR
GSKAILNFPLEAGTLSKTAVASAVICGAKRCRDVELKSEQQAVKKVWLVIMSTPDEFSALA
LIRQHLFGEFSPVTDLSHTPSFSTTHYCTIRDSENSSSQSDSFCSQTASSDCLNFNELNNTKIF
DHVSDSMNYEPNHDLFEFQSNPQIIELTTPKAFHHTKIFDSASDSILFEGNHHDIIEFELKPQ
TINLATPKALNSNNQSSSASRSKRSLKIDLAPVKKFEWLNFREPTKIAVSVEKPSIADERKH
YRGVRRRQWGKFVAEIRDPKRRGSRVWLGTFDTEIEAARAYDQAAFKMRGTKAILNFPL
QAGKLCPSGASVNGGMKRCREVEERPEKRR
>Actinidia14407.t1
MATAAAATVSITLKKVQKSDSEKHPIYQGIRKRSWGKWVSEIREPRKKSRIWLGTFATPEM
AARAHDVAAITIKGQSAHLNFPDLAHELPRPASKSPKDIQAAAALAATLATSRSHEAEAES
SQAESTSSQSLDSSTRDSSTSHIIKADDPFFDLPDLFLNLEHRTNDLHYMLTSHLARDEPFG
GEFWPEDPFLVGNDENRPYALAHIALPPKTTATMEASINATTADRGGCPGKGIITLTFSTTG
ALNYLHTRIDSSKKYYSITSLN
>Actinidia05451.t1
MSKHFYKQPPLEDKIHGTEVFAAKFCNIYNNTRLAPTQYKKPLHKSQLINNCTGGGRGNV
AAGGARGSGRHPVYRGVRRRRSSGKWVSEIREPRSPNRIWLGTFPTPEMAAVAYDVAALA
MRGQDAELNFPNSASSLPVPASMAPRDIQEAAASAAAAAGAATDALAGSTQAHLNETAN
PSAGGGGSEFVDEDLIFDMPNVLVNMAEGMLLSPPRLDVAADHDTAAENAGDQNLWNFP
>Actinidia28819.t1
MAPESSNWLSFSLSPMEMLNSSSQAQMLQSTMKNVPFDGASGDSQQYYFLDNFYGNGW
ENKAQEVTAQSIADSTIFTTFLDSQIQHPPPKLEDFLGNDSSLVRYSDSRTETQDSSLTHLYD

HSGSVYFADHHDLKTVPATAGFQAFSGNSGSEVDDSASVARTQLVSTESGTELGFSQCPNG
ALSLGVSARGSEKALVSVDSESCKKISDTFGQRTSIYRGVTRHRWTGRYEAHLWDNSCRR
EGQARKGRQVYLGGYDKEDKAARSYDLAALKYWGPTATTNFPVSSYTTEIEEMKHVTK
QEFIASLRRKSSGFSRGASIYRGVTRHHQQGRWQARIGRVAGNKDLYLGTFATEEEAAEAY
DIAAIKFRGVNAVTNFEMNRYDVEAIMKSSLPIGGAAKRLKLSLEAEQKPSLNHDQQPPQ
CTTSSSINFSTIIHPLAAVPCGIPFDTTARYHHNLFHLNTDGASDPPGSAAATLHGDDVYFTF
EDCSQEREGVVATKTALLKPQSKAAIGVGSRYGEKDMDNMSQKFRNEELPETWCSQFFE
KYKATKPYRGVNQRGGKNGSEGNRKHFGQEQASEMSRKVIARMLTNMGFEASSEVPME
VLSQLLSCHISKLELVKDSTRNSGQQTPQQLQVMQSQIQSQQQAAIRQPQQLRISEYFTTAT
AWDRMRRRQPATPRPGMNMNMDNAIGFPSNSPNSDTEHRNMSTVRAQPVKVEDFQELG
GDASLKHDSEENKLTSPPK
>Actinidia04754.t1
MCCSKVADRGETGDFVRFPATDADDDSGALPPDAAEQAAVFSGYSRSGEMTAMVSALTR
VVSGDSGTALFGGATTSIGVSGSSARVKREREEESVSTQFTEQAQRVYRGHSSHFRGAHGE
SSSSAVPEEYTRIVPPPTMTIAATATTPRPEASPQEETGERRRYRGVRQRPWGKWAAEIRDP
HKAARVWLGTFDTAEAAARAYDEAALRFRGNRAKLNFPENVRSLPPPPIAASPVTNLPAP
VIFPTQQAQSSDIVRDYWEYSQLLQNTGDFQSSRLLQQMFFASSVAGLDSHSLGSSSSTLF
ASDSSVSSSSSLYPLLFSGQQTGNLLPPGGQSQSGGGGGSDFPAPPWTGSGHYPPSSSS
>Actinidia29544.t1
MCFPKVADRGEPRDFVRFPATDAADDSGAPPPDAAEQATAFSGYIRSGEMTAMVSALTRVI
SGGTGTASLGGGTTSFGVSGSLPGVKREREEESVSTQFTEQAQRVYRGYSSNFRGAHGES
SSSAAPEEFTRIVPPSITITTAAPLLPPPEVSPQEVTGERKRRYRGVRQRPWGKWAAEIRDP
HKAARVWLGTFETAEAAARAYDEAALRFRGNKAKLNFPENVRSLPPLPIPASPATHFPAPAI
FPTQQAQTPDIVRDYWEYSQLLQNRRFSAQQVVTANVFRFFSGWFGLAFIGL
>Actinidia22728.t1
MQENNTQFTFSNSSNNRPSLSELILSGGNNTLNSIISLYLVSNLMNCPVLETLCSSVYLQQR
DLLQKFYEENRSNSTISQISTKKQFKLCFTIKYIIQQEKAIQGVEQRHWGRVWLRLCSLRTE
MKVFVGEYFRAAMAAYAYDRAAYKLRGEFTRLNFPNLRDPTNLGFADCGRMNALKSAV
DAKIQAICQKKEMEMDSSSVVGNDCWSSEVVSPTVSEDGLWKGENSRSSVSGGCPVGVV
NELDVGDWSLKRMPSFDPELIWEVLAN
>Actinidia10847.t1
MATLDEVSALELIKLHLLGEFSPVGKFGAELSEPQIIDLATPKPVDLSPKSSSQSRLVERRLK
IDLPPVKPPVTKLEWLEFSEPAAEVSEQKPSEAEERQHYRGVRRRPWGKYAAEIRDPKRR
GSRVWLGTFDTAIEAAKAYDRAAFEMRGSKAILNFPLEAGKWHNTAAVTSVDSSRKRTRE
VEEMVEEKPVKKEKVPESDASVLTQLTSCPLTPSSWTTIWDQNASGIFNVPMLSPLSPHPM

MGFTQLVVT

>Actinidia08430.t1

MSKHFHNHVPWMIRSMEQKPHNIFMDYSSGGGGPSGRGGNVAAGDARGSGRHPVYRGV
RRRNSGKWVSEIREPRSPNRIWLGTFPTPEMAAVAYDVAALALRGRDTELNFPNSASSLPV
PMSTAPRDIQEAAASAAAAAGAATDALAGWTQVHMNENANRSGGGSEFVDEDLIFDMP
NVLVNMAEGMLLSPPRLNVAGDHDTAAEYAGDHNLWKFP

>Actinidia16565.t1

MASSTEGHFRGVRKRPWGRYAAEIRDPWKKTRVWLGTFDTPIEAALAYDEAARSLRGHK
AKTNFPSPPRQPELSLNLHSPTPACRPIGHFLRTGVLTEDMGSVGNASVNDGPFAGFSGEG
TVPEISSPAGAMFPHGFVLVQ

>Actinidia01945.t1

MVALPQGLYSLPIELRESYNAMSTSSKNHQKPSQPYDPTPVHTGFSILQRNTSPPQPRERRG
RGKQVEPGRFLGVRRRPWGRYAAEIRDPTTKERHWLGTFDTAHEAALAYDRAALSMKG
KQTRTNFVYSHQHNNALTPFDLQTLIQPSHQSHEQFFITINQKRPCQPDTTCQAGTHIVNHS
DNDSSSQSSYGSSPNDSNNFFSGENNSGYLGCIVPDSCLNPNTPKSSKDGNFSSYASGQTH
FDDLGMAGMGYDSNPSDDLPCFDGLSYGFWGCDQTWELNSCEFPAMSDNINPLGVDNG
CMENLYPNSGNPMPTVTTSSSATCSSLIPPFGDGVEFGYTLF

>Actinidia06886.t1

MNAHGGEVPKVADFLGVSSKSESNQSDLVPYNEIQGNETDYLFSSNNALVPLPLHNTLGIG
TPSNYELQENPTTLQSLTLSMGSGKGSTSETGVENSNVSNSTIEATPRRTLDTFGQRTSIYR
GVTRHRWTGRYEAHLWDNSCRREGQSRKGRQAGGYDKEEKAARAYDLAALKYWGTST
TTNFPVSNYEKEVEEMKNMTRQEFVASIRRKSSGFSRGASMYRGVTRHHQHGRWQARIG
RVAGNKDLYLGTFSTEEEAAEAYDIAAIKFRGLNAVTNFDMNRYDVKAILESNTLPIGGGA
AKRLKEAQAIECSQNERK

>Actinidia06078.t1

MRRDPTVPSISCNNQPAEQKQPQVDQTSSLTSMKRSSRFRGVSKHRWTGRFEAHLWDKLS
WNIKQKKKGKQGAYDEEESAARAYDLAALKYWGTSTVTNFPVSDYEKEIDIMQTVTKEE
YLASLRRKSSGFSRGLSKYRGVARHHHNGRWEARIGRVFGNKYLYLGTYGTQEEAARAY
DIAAIEYRGIHAVTNFDLSTYIRWLKPGASTQIAAPEVSSSSFNPQEFHAHDFSVDDLNSPQ
KQELSERKHPVSSDKKSPPTALDLLLRSSIFRELVEKNSKVLEDENGEDMDSQPQMGTDD
EYPTLFYDVPFELPSNSDNVELQGEFHFRYTNPQCNLQLGTTE

>Actinidia25486.t1

MAKISQINGSNTSSSSNNNNNTATKTKRKRKSVPRDSPPQRSSIYRGVTRHRWTGRYEAHL
WDKNCWNESQNKKGRQVYLGAYDDEVAAAHAYDLAALKYWGQDTILNFPFSTYQDEL
KEMEGQSKEDTLGLAKEHHHNGRWEARIGRVFGNKYLYLGTYATQEEAATAYDKAAIEY

RGLNAVTNFDLSRYIKWLRPNQTNPNTPNNPQPNPNPTNETALNFLHQQSCSAAVRATTPP
PRPAAGTTTSSALGLLLQSSKFKEMLERTSAADCPSPPQESDPPRSSFPDYIQTYFDCQDSS
GYADGDDIILGDLNSFASPIFPCELDA
>Actinidia23158.t1
MRVRRDGLTVHNRRSMTSGDSSTAFRRRVVRVIFTDADATDSSGDDEEQAVRRVKRLVKE
ININPSPNKREQSKKRRIGSLGSAGSDASRRKKFRGVRRRPWGRWAAEIRDPTRRKRVWL
GTYDTPEEAATVYDRAAVKLKGPDAVTNFPNVTVTEKAVHVKTDGEGSDNVAALSPTSV
LLYDELTPFDEFSYGDVDAFGFDIDMPLRLADFTVPEDLYGDDDLGEEFNLDDFLVDDR
>Actinidia00657.t2
MSSEIERIIRMGFLDHASNMASVPLDYSRKRKSRNRRDGPNGIVEKLAKWKEYNEKLESL
DNSPKPVRRAPAKGSKKGCMKGKGGPQNSNCNYRGVRQRTWGKWVAEIREPNRGSRLW
LGTFPTALEAARAYDEAAKAMYGPSARLNFANGSSFIESSVESASLPTTSGSGSSTSSSYSE
VKLHAPKAKHEDGDDESRIGGNSPLASNNMPMTAVKEEVNDEPVEFNGKGEQMELIGKP
SLDYILEDFSLDETFDVEELLGILDEMGQSSDRINFQGEKPSDLSYQLQNPDAKLLGSLNH
MEQAPPVVDYGLDFLKPGREEDFNFALDDLGFLQMDSDLGL
>Actinidia12165.t1
MGEEQSNIESEAKEYYYLPSSSSTTTTTSSSSSSIATATTTASFLSKFDCVNSLSQSTRSTPKE
GKAIKGQRRVEETKNGNEHRKRHKSGGDEKHPTYRGVRIRNWGKWVSEIREPRKKSRIW
LGTYPTAEMAARAHDVAARAIKGKSAYLNFPELAHELPRPATTSPKDIQAAAAMAAASLF
SGEATRAEPSRAGLPCSHSSTTLSSSENTTQESLAASELSFRLDDPFSWEY
>Actinidia03244.t1
MENCERAPLKPWKKGPPRGKGGPQNAACEYRGVRQRTWGKWVAEIREPKKRTRLWLGS
FATAEEAAMAYDEAARRLYGPDAYLNLPHLQANFNPLNKSHKFKWLPSKSFMSMFHPNG
WLNLNAQPSVHFIHQRLQELEKNRGNSSSSALKTKTKNPIISDKAPVETFVPIEKEVGISSG
DGKEKPQIDLHEFLQQLGILKEESSSRGSNVSESFVAVPESSLTEDDDVTALAEKSFNWDTL
IEMRDIEDRQGVETSNFQHVSVRTGK
>Actinidia01350.t1
MEAALKRLNDSLTRQPESGPLQPLKRVTTAANKSSLRDGGCGTQMRYRGVRRRPWGRYA
AEIRDPQSKERRWLGTFDTAEAAACAYDCAALSMRGVKARTNFAYPTATSPTPPPSLTDNF
LPPFHYKKRSQPSVRDIPTPTRGFISPVSIPSGDFPVQAPQRSTSLNTVVFPHVFKPSLAYEPF
PNVSLSTSVCVHTSENFPGSNQIRPKYEDLKSGQSDDGLDFFGSKPSDSGLLQEVLNGFYP
KPASKCDESSTQNQNCTHEAFDEMTELKAIILSWYTPRPFLIPLEERERERERERTMEMNE
EITINKYMKVEIPPETQEVVSPPGQSGLARQGSITKTNCLCSPTTHPGSFRCRLHRTGPALQ
RTKSVDSKPNPSTRMVEPQWRPSNF
>Actinidia23663.t1

MVLSSQIELPLNENDSQDMVIYEFLDQANALSTPSLSLESQTVTPNVLKPNTALGKKHYR

GVRRRPWGKFAAEIRDSARHSARVWLGTFGTAEEAALAYDTAAFRMRGAKALLNFPTEV

VAASAAQGLESKCSIYDVEWNHK

>Actinidia15099.t1

MEEAMRRLNGLTHTPDSEPLHPTSNHLKRPTTTTTAATNKRPLNDTAAANPTRYRGVRRR

PWGRYAAEIRDPQSKERRWLGTFDTAEEAAFAYDCAARAMRGVKARTNFVYPTLPLPHD

PLSHNLIPPFTYKDASQPLDLSNPNISSVNALVFPQLISGSSSLTPFDHNSLYGSNSLVNPLNT

VQKPSTEYDASVIGDNQVDGMDFFRSEPSDSGLLQQVLRGFFPKPSTTKNCTTEPSKTLNC

TAESSSKGLINQTLDETKKGIESDNLGLYRVPPDMVWGDVFQWDVYGPYYHTRDRQRDK

SLKPSALLIQDRSPCIFCNLLSLASPDRRKKTEGSSMESEEIRNKDAKLGILSESEIGSLTSAA

GLARQGSVTKNNCLCAPTTHAGSFRCRLHRNTPTGLQRTQSIESVSVKDSNSKANTSTATE

PTNSTGSTVEAP

>Actinidia02179.t1

MYTEAEFDADLALLESITRHLLTESELAPAISGYAPAAHCLGTDYWGDLPLKENDSEDML

VYNVLNGGLLQQETAAGALAGIGSFPAAAPKMEAPEVMPVVVPEKGKHYRGVRRRPWG

KFAAEIRDPAKGGARVWLGTFETAEDAALAYDRAAYRMRGSRALLNFPLRINSGEPEPVR

VRSKRASPERSFSSASSEDGSSKRRKKAVAAAAAEPVVVQPKLEVVGDELDFAQIWDYST

WQESIGSGC

>Actinidia24184.t1

MNSEDESSTASSPSSPLPIVLHKRKAGRKKFKVTRHPVYRGIRRRYGNKWVCEVRKPNKN

SRIWLGTLPSPEMAAQPHDVAALALCGRNADLNFPIPPRSFPRTKSASTWDIRLASVHAAR

ALPFPPLSCSSSTNFSSHESTCDAKAFVPLSRCTLTHTIVPRKSYIEVKPNSFSLNFSQDPLVQ

RISSPVLASQDSCIEIPTKSYTQCIETERHDFFRTENPCSRNTSIRNAVIQRLPSVKCQKNSVV

EPIMVDSSNFESHKIGIERFSSDSTCVEGSNSVFSDPLVPVFVDEEELFNMPALLDSMAEA

>Actinidia26138.t1

MAAIACNGLIWRCRAIAVAIDMIHDDNSQGWRGKGRVALNVVVMAPKAATVVKGIGKE

MHYRGVRKRPWGRYAAEIRDPSRKSRVWLGTFDTAEEAARAYDAAARDFRGAKAKLNF

PLLEENVSNDFDFKNKVEIKKSNCNQSPSPSSTVESSSPVAGSPPPLDLSLSFGGGARFPFQ

QYQFVSPVTGVPPSANHILYFDSIFRSARMNQDYLRDQRVGRDHVSHVKAATAVDFRGGG

GAQSDSDSSSVIDLNLNDVKLRRAIDLDLNLAPPPEY

>Actinidia20193.t1

MALKRILKELKDLQKDPPTSCSAGPVAEDMFHWQATIMGPPDSPYAGGVFLVTIHFPPDYP

FKPPKVAFRTKVFHPNINSNGSICLDILKEQWSPALTISKVLLSICSMLTDPNPDDPLVPEIAH

MYKTDKGKYEATARGWTQKAGVTSTSFVSPTPPSEEESKARDRDQNRKRLQQCESDCEQ

EEEAEEEGHFGEYGGGGFGEQVPAAETSYLLCNVLAAEIRDTSGARLWLGTYDTAEAAA

MVYDKAAIRIRGPNAFTNIIEPPARPPPTPPAIDLTGKDRRNLTSPTSVLRFDHEIQSLQDDC
LPLDPCFLNDFFDFRSPSPIIFEEVCLMDNVLDSNLSDLCDVSPVNLDEDFRSCAWDVDDF
FQDPLVVV
>Actinidia03155.t1
MDESMSVLRPIKYTEHRNVTKKLTKPSIPRSKKISDDHLQSPELCSGGSRVVRISVTDPDAT
DSSSGEEDALFGRRRVKRYVNEIRCETACKSSLNANKVWKRKVGGGNSLLAKRKPTSAA
SGGQKYRGVRQRPWGKWAAEIRDPVKKARLWLGTFETAEEAATVYDNAAIKLRGPHALT
NFITPPLCESPENNVKSVSGDESGDESHSHSLSSPTSVLRFRTHSSEEAEPPKLSKPVQQVN
ESNDSSPVQEDPCFLPDFMPMEMPFLDDIFTFEPPGPIHFDDPPDFADHLLSDDLSGMVTDP
IHDFGLWQVDGFLEDLGGDFFTADPLVVQMFPFVLTEMEWSFL
>Actinidia30245.t1
MVKPETSNQAERSTTDESKYKGVRKRKWGKWVSEIRLPNSRERIWLGSYNSAKKAARAF
DAALFCLRGPSAKFNFPENPPEIAGGRALTPAEIQVAAARFANSEPGTSRSRRSSSSSSSSSS
TMHVVESPSPSVSDGVAPMGTETPLDGSLLDLFATMGSENTGPGSFDIFPEFDDFSNEFFVP
PLPNVDYGEENWFLSSTSVSSISNVVLSLHHSYLLSRNYFNPLNLVPRGMATAQLTASSISA
RNLPSFKGFTPSTARVLSVAPLRQGGLGLGPFRGLVVKASATIAPKYTTLKPLGDRVLVKIN
AAEEKTVGGILLPTSAQTKPQGGEVVAVGEGKTLGKNKVDISVKTGTQVVYSKYAGTEV
EFNGSKHLILKEDDIVGILETDDVKDIKPLNDRVLIKVVEAETTTAGGLLLTESSKEKPSIGT
VVAVGPGPLDGEGKRKPISITPGNTVMYSKSLRPEFVAVITPGVFGQIVTPLNLVTTVACGD
FWSNRKPIQARIWNLTVNFNLLALAVAGNVSVKS
>Actinidia16494.t1
MQLWKLRTRGLMAQVLHCPEKDELEPERSLTLPSRLTRERELSIMVSALKHVIYGNGNDV
SPANIVALQDATSSSLAPPSGTKIQPMLVIPEGDTCQLCKINGCLGCNLFEPTSTTTNGVMK
RVKKYRGVRQRPWGKWAAEIRDPRRAMGVWLGTFETAEDAARAYDRAAVNFRGAKAK
TNFPLSDYWIKD
>Actinidia35451.t1
MMNLEDDSSPYSSSSSSLFSNITKNPSSSSKPTNVPHPTSHKRKVGRKKFRETRHPVYNGV
RLKSCDKWVCEVREPKKKSRIWLGTFPNPEMAARAYDVAALALYSEMAKLNFPDSATLL
PRAKSSSARDIQMAVLEATRAVQLTIPSPSSSPSSSLLSSRRTSLTNHTSPRLTSIPRVKYQKN
LLKPAVVPEDYSVVSVRDSKNVLDSSETGFVDEEALFNMPGLLDSMAEGTRISFDVPDHV
PNICNECEKPHGNCGIGLRCICHQKECKDKVISNSVTIDSSGNIIFSLLFSFIFVVVFVKG
>Actinidia33507.t1
MYEQTVSNTDFALLESIRRHLLEDSDISDFFSATDAPNTLPIYDPSRSFESIVEEPVGERSDH
SPPVEWRKYRGVRRRPWGKFAAEIRDPKKRGARIWLGTYETSEDAAVAYDRAAFKIRGSR
AKLNFPHLIGSNNLSRSGKFDQEVVEVRSH

>Actinidia35067.t1

MSLETQNLNENGEKRSLKLPKRFLGVRQRPSGRWVAEIKDSSQKLRLWLGTYDRAEEAAV

AYDSAARLLRGRNAKTNFTYNGIMTTHQENNNLFGKNPRLYRLLQHAIMKNLAKSSSLN

SANLEADSNFGGVVEETIVCSIDKDHNQLCSLSLGSSRVYSSVIVAPSFSASPSCREGQQLD

ESMKIGTAPASAPSMATMPPSTISSALYESPSTPPNVASLNRVAVAGSAAVVFFAATLLSRSTS

PAASGGNFSIPKTPTPSSSGLLLPKYSTKSSKELDEGDGGRSRHSD

>Actinidia29458.t1

MVRSENKIQSEAKKYKGVRMRSWGSWVSEIRAPNQKTRIWLGSYSTPEAAARAYDAALL

CLRGNLHHHSTTVPAAAAALFLRLVTVVFVFPSNHIDDDVDISLVSPLYEPQMSLVTPWYN

FDSPKYNDMMHNGAIFDPPVMDDAFEEGDIQLWSFC

>Actinidia08136.t1

MEEALRMLNDSLIHQPEPDPFLQPLKRVTTAANKRSLRDGGEGGCGTQLRYRGVRRRPW

GRYAAEIRDPQSKERRWLGTFDTAEEAACAYDCAARAMRGVKARTNFAYPTVTTLPPPPS

LTDNFLSRFHCKKPSQTSVRDIPAPTRSFVSSLSSPHGNFPMPTPQRSTSLNTLLLSNLFKPS

LTCEQLPNVSPSTSVCGNAYENYPGSNQIRPETEYHNPGESDDGLDFFRSEPSGSGLLQEVL

NGFYPKPASKGDELSVTQNQNCTNEAFNEMMGIESDHFGLHFDGFGSPAVAHSSISVPEES

MLCEHVFQFHELASPDPLGGERERAS

>Actinidia26001.t1

MCGGAIISDFIAPTRNRRLIENLLWPSDVKKNHSNYHSKPLRSEIVDLDDDFEADFQDFKD

DTDAEDNDFKSFVFSASKSGVSDGSRSTKSVELNGQTEKTAKRKRKNQYRGIRQRPWGK

WAAEIRDPRKGVRVWLGTFNTAEEAAIAYDAEARKIRGRKAKVNFPDEAPPNAAKRAIK

ANTQKVLAKESINSVQPNLNQNFNFPNNPENDCYSMNFEEEKPLTGQFGYTEAYPVPGEV

GHKSSTPSAGATPLYFNSDQGSNSFDCSDFEWGEYNTKTPEISSVLSATIECDEAQFVDNA

NPTEKLKTKHEDVGPVQENTVEKLSEELSAFESQMKFFQIPYLEANWDASVDTFLNGDAT

QDGGSSMNLWTFDDFPSMMGGV

>Actinidia18234.t1

MARPQQRYRGVRQRHWGSWVSEIRHPTLKTRIWLGTFETAEVAARAYDEAARLMCGPRT

RTNFPYNASEAQSSSSKILSATLIAKLQRCHVTSQQVSNKPKTKEPHPYEAQSHHVVSNEG

QVGSEQQFKALEDDHIEQMIEELLDYGSIELCSVLPN

>Actinidia16620.t1

MESSCIDESTTSDSLSFPPARTSPAMVKSPQSLCRMGSGASVVLDAEGGVEAESRKLPSSRF

KGVVPQPNGRWGAQIYEKHQRVWLGTFNEEAEAARAYDIAAQRFRGRDAVTNFKPLSES

EADDEAEAAFLTEHSKAEIVDMLRKHTYGDELEQSRRSFGGKKQCKEKISNASGSDKTIG

KATQQLFEKAVTPSDVGKLNRLVIPKQHAERNFPLQSENTSKGVLLNFKDVAGKVWRFRY

SYWNSSQSYVLTKGWSRFVKEKNLKAGDVVSFHRSTAVHDKQLFIDWKVRGDGSSVAGF

PVQVQPVHMVRLFGVNIFKVPFSGGVVDSSGGCGGNKRIRELEMLGFGCSKKPRVFESRL

>Actinidia36473.t1

MWDLNDSPDPTTRDDESEVCSYGDDDKGKRVGSVSNSSSSAVDDEDGGGRKRSGSKIFG

FSVTRPDDSFSSESDPPVTHQFFPASDGGGGAANFPRAHWVGGRFCQSEAPVAAAGKFTE

TTQPLKKSRRGPRSRSSQYRGVTFYRRTGRWESHIWDCGKQVYLGGFDTAHSAARAYDR

AAIKFRGVEADINFTLEDYEEDLKQMSNLTKEEFVHVLRRQSTGFPRGSSKYRGVTLHKC

GRWEARMGQFLGKKYVYLGLFDTEIEAARAYDKAAIKCNGKEAVTNFDPSIYEDESIPLN

VQASHQTIIWI

>Actinidia27084.t1

MPQGYGPGPGPSYGYMPPPEYGGVGGRDVDGGMMSEVGEKTAEAVEMAAVAAGEMAS

YAGTMKHKSTPSSSSTSATTSAPTVTSRSYQGVRLRSWGKWVSEIRLPRQKSRIWLGSYPT

AEMAAQAHDVAALAIRGQSATLNFPQLVHKLPCPATVAPKDIQAAAALAAAGKFDGFEIE

DQAKTTNTTTSSCEGQYLGRSLASESSDDVDALFDLPDLLVDSKMDSSNGFCFASLFNCID

IGFQIEEPFLWD

>Actinidia05650.t1

MASSTEGHFRGVRKRPWGRYAAEIRDPWKKTRVWLGTFDTPIEAALAYDEAARSLRGHK

AKTNFPSLPPPPPPELSLNLHSPPGRLPIGHFLGTGVLTADVADSSVTDGRFVGVAGEGPVP

VPESSAPAGCLGVDGFFIE

>Actinidia38946.t1

MLLFEVITEGDCSTIDSLMGTSSEEVCSEISGDPTKEVAYRGVRRRPWGKYAAEIRDSTRN

GVRVWLGTFDTAEEAALAYDQAAFAMRGSAAVLNFPAEVVYESLGGMACIFEEGCSPVL

ALKKKHSMKRKGVRGKKKEKENQLRMENVVVLEDLGADYLEELLGLSESTSVGRAQEF

GAINRPDERNMGLTPSHGSAKSIGRLRSPSFMSRPVKGGGGGGAAGGNGRRGG

>Actinidia04453.t1

MDLNSSVFHSQYSDFSPESSFGSPPESFSCNQLTFINSLPFNENDSEEMLLFGVLAEAAWES

SENISSDPNKEYEVSSKPEGKPVKEKSYRGVRRRPWGKFAAEIRDSTQKGIRVWLGTFDSA

EAAALAYDQAAFAMRGSSAILNFPPERVRESLCEMKYNFEEGGSPVVALKKRHSMRRRIV

GSKNREREVKTETVMELEDLGAEYLEELLSSTESAGGPNNLGVRSNLCHAVAKPLNNLKI

AV

>Actinidia32541.t1

MTSNNVDRKRRRCNDCESIEETLLRWKNYHNQFGSDQFDPTNNGVKRRRKGPGKGSRK

GCMTGKGGPENLGCRFRGVRQRTWGKWVAEIREPVVGRSQRNKGKRLWLGTFPTANEA

ARSYDIAARIMYGSEAILNFPDKYSKATDSSDESRSITTTLPVSTLESKPISGDCEVGADVE

QRACSEVYIHNESKVKLEGTLRECTGEESMKIEVATPKQSEVMKEKIAEVSYSSGVYDHD

DEQDCSQSLKGETKTTGSGECDLRRDSISLSSQEGSLKHETPSDDSCQLHNPEDQNRFQNS

RTDETLEEIKPFDVISIDGSAFRQDGNCYEPFQYQDQLDCMESMLMEDGFGFQHLLWAIG
MDDYVGSVNIEGHLQNERPCDPQYPLENEKVCEYKQNLLTGETRDTEPSWVDRTRGSEL
RPEGDSDFDMFGFSSDRFPNLETQNYMFNCNSLIEETCDEKKNSSGASITEKQENLECVRP
LEIYELHNEGTDLLDNMEETPVREDSSFGLLDSSFFEDLDIWIPNACNLNGDTRAVL
>Actinidia33993.t1
MDLINSSNKANSPLSSSTSSSSSKSKKKNKQQSQKDLNNSGGLRFIGVRRRPWGRYAAEIR
DPLTKERHWLGTFDTAEEAALAYDRAARSMACACKRRNTIPIPCARLAPTSSTLTCLLQNQ
PPPPLPPSGFFPVPGDEWIQRTTLTDYHNQQQLQQQQLCDEQKIITDGFITHGDVGGGGGG
GSTKTNQHYYCNVDEAEAELPPLPPDISNSYDSRSVCGVHVHGSVWNEEDPMSNLLTDN
GRFSEQQTTTGHYYHDPWTGSSSTVGIESCNNVHVGFLMESASSSELINPLFRMTEPVSAA
TAGILSPSINIPVPAEAFDFGGCCSSSSSYSSSAYFF
>Actinidia05276.t1
MLDLNLAVMPTDSNSDGQMDDSGASNSSLFNVETSSVAGDEELSSTHDNTFAYSFGILKN
YESEENNANREARLGFVAKQLFPASDGGGGREWLDLSCKAASSGGVAEQRGLVHQQKQ
QVKKSRRGPRSRSSQYRGVTFYRRTGRWESHIWDCGKQVYLGGFDTAHAAARAYDRAAI
KFRGPDADINFNISDYDEDLTQMKNLTKEEFVHILRRHSTGFSRGSSKYRGVTLHKCGRW
EARMGQFLGKKYIYLGLFDNEVEAARAYDKAAIKCNGKEAVTNFEPSTYEKELSSEAENG
ENNHNLDLNLGITTNYFADGQRGNNDLNSGLHYHCGPTDMHASIRAENSAPTMATQPPH
GQIMVSDHPPLWRGVNSNVFPIYEGRAIEEGMEFYSSPSWAWQGPFSRTASLPLFSTAASS
GFATVAASSAAVHQPQFLNTTFPSHYPPPITNSNNIS
>Actinidia24950.t1
MAIETLPFRKTAAVKDHPTATATATAKELRYRGVRKRPWGRFAAEIRDPWKKTRRWLGTF
DTAEEAARAYDTAARSLRGHKAKTNFSHAVAPPKPIQLEANSSTVPSVKSDAIPANLTHGC
SPAFLSADNLGRIFFAPGLPLPARSEYSGYKLETVVTITSSCTVDKGSNEWFVSVVLLS
>Actinidia07595.t1
MSSSREDHYRGVRKRPWGRYAAEIRDPWKKTRVWLGTFDTPEEAAMAYDGAARSLRGA
KAKTNFPQPHPLPELSLDLNLAADQHWASPAGRLVIGEFLHTGVLKEVRSNGELTVNDGG
GGLSSVELPAAEKSAPAGYFGVFRRCSMKWDLHGLIKNVASSQPMIGVGGGVKVGPNAL
TSEDVGSKGVTAVLRVVRMGGERDDSATLSVTAAASTLTVVLLGCNFLARAPPIIVIVLTAP
LVRE
>Actinidia09555.t1
MDSANGRYVGLKILYRPRPAGRLDHSPESSLGSPQSFSWDDLLFDHNSLPFDVNDSEEML
LLGVLAEGASKESSETVSSGWIKEEEVTSVAKDQKPVKEKSYRGVRRRPWGKFAAEIRDS
TRNGIRVWLGTFDSAEEAAMAYDQAAFSVRGSMAVLNFPVERVKESLREMRYGCEEGCS
PVMALKRRHSMRERSSSKRNKVNKEVKVENVMVLEDLGAEYLEELLNSS

>Actinidia21025.t1

MCGGSILAELIPRNGNHRLSASQLWPNSPFVTKLKPPQDQNNGDERVEKKAKRQHKNLY

RGIRQRPWGKWVSEIRDPRKGGRVWIGTFNTAEEAARAYDREARKIRGNKAKVNFPNED

DHSIPFPPQANPQPAAMTHPNGGFRDNLIQFGAYNSDGFHSVPCSDPVSVLHSEEVFGSGL

ECAYPSMDCKLKVKEDRENKEEREYSKEAAVIEVEETAGEEIELEKLSEELMAYESIMKFY

QIPYLDGQSPDSLPNTAAENDVIGCGAVELWSFDDFPPPFA

>Actinidia06788.t1

MASTNNWLDFSLSPQEHQNPQDHSQNSVSRFGFNSDEISGTDVTSDQCFDLTSDSTVLPSL

NLPAPFGILEAFNRNNNHSHSQDWNNNYKTTTDFSMLMGTNNHSLENQGPKLENFLDDH

SFAHHGHYMYPLPEAADTANNNNSGATTTIIGLSTIKSWLRNQPTPTPAQPENKNDQGCG

VEGSNGNLSLSMSSESQTGSPLPLLAVSGGNGSGGGESSTSLDSKQKGTILDAQTGAIEVV

PRKSIDTFGQRTSIYRGVTRHRWTGRYEAHLWDNSCRREGQTRKGRQGGYDKEEKAARA

YDLAALKYWGTTTTTNFLVGNYEKEIEEMKHMTRQEYVASLRRKSSGFSRGASIYRGVT

RHHQHGRWQARIGRVAGNKDLYLGTFSTQEEAAEAYDIAAIKFRGLNAVTNFDMSRYDV

KSILESSTLPIGGAAKRLKDAEQAEMTMDTRKRTTDEIITTSHLTEGTSNVYGTTHYNWPT

VAFQQAQPFSMHYPYGQQRVWCKQEQDEIPPHTFQDLNQIQLGNTHNYFQPNNVLHNLM

SLDSASLDHSSGSNSTVYSNGVGDGGYVMPITTVIAQEGNNTQGNSGFGDSEVKAYGYET

VFGSSDAYHGRHLYYLPQQSQGGIGKAASLYDQGSMCNNWVPTAVPTLAPRANNMALC

HGAQTFTMWNDT

>Actinidia30547.t1

MKQLLCYVWICLDLGKDVARNLVSGVGSQSCIIRISSRASSNLSLLFRGLGALSIETTSLVL

HSFAAVTYEIAKIRSIELTSRAGEGSEAGHQVSGPFSKMPKFKGIRGRGNGNNKFVGVRQR

PSGRWVAEIKDTTQKIRMWLGTFETAEDAARAYDEAACLLRGSNTRTNFITHVSHDSPLA

SRIRDLLNTKKAGKKPSENIYGGINSSNTYNSHTPPINTTTNIPSTSTNSCTISNSDNETTSSG

KLQDTQLFDDAYKPDLSNCIEEFELGTSQSGMFGQAFDRFPFNQELLELPRNVVSHEASEL

EFSEFDRMKVERQISASLYAMNGVQEYMETVHDPSEAFWDLPPLCSLFC

>Actinidia14886.t1

MPEPQVQSPTNQNLSKRPKNKSVSAGENFKPRKIRVICSDPYATDSSDDELTEKPYGPKRIV

QEITLRDLGRTKTPETYSSCDDSNINGEKAPTRKRKRVLSRTQNPYPGIRQRKWGKWAAEI

RDPFQCKRLWLGTYNSAEDALKAYNNKKLEFKARATVSDKSLNRSSMTESNPEDNHPAV

SEDSVSLLPHKSPSSVLELDSPATTSLINAEKYSETVKDFGVETNFDENEVPIVEEPLGLDLG

VELDLDSMFMDDFGLLLDDFCSLDDFQIHGFDDNQPTDLPDFDFDLGNEELSCWIDEPPNI

A

>Actinidia18228.t1

MSYPSLQQIPFMPRPDPFPFKPTQTFPILTEFPIFYYDFPLFGSPDLSFSRGHTQIPPDPPTQKP

ETPPVLEGIAAVVGQHVLFGNGDTPCGDSGTHRAGQSPPETAREKKVVVPVQKSYRGVR
KRPWGRWSAEIRDRIGRCRHWLGTFDTAEDAARAYDSAARRLRGAKARTNFTIPPVLPLL
SPSSSSSSSSDAKKTRGKAAAHNSRKCAVVTSVGQLFSSSSTHTNRTHVELDLKLGASFNR
>Actinidia40302.t1
MCGGAIISDFVPDYHRRKLTTRDVLSELDPFSDFSGLDHHDIENRPNESNSKTTSPKPKQIN
KGTSVRTRKNKYRGIRQRPWGKWAAEIRDPQKGVRVWLGTYNSAEEAARAYDAAATRIR
GGKAKLNFPIPPPVKKRCVVPESTRVTQQSTQFTYSGFEFNERISDLESFLGLDSEPTQFGD
SVSPTRWIW
>Actinidia09869.t1
MASSRKSGKSKKGMEESQKMTQMEWEREHEIGLEAGQWRPVFDGASASTRPLKKVRSP
DRRHPFQSSSSSSSSSQPTPLSLYIPSSVPQTTTATTPTALNPFQSNSRLVFPFAFDGSQQQSIE
IPQQFRTNPPLLIPPHPQNQQQMISFSPKHHGIAYPPYFSGDLASLQQQHLLQYWSDALNLS
PRGRMMMMNRLGQGGVRGLFRPPVAVPAISTTKLYRGVRQRHWGKWVAEIRLPRNRTRL
WLGTFDTAEDAAMAYDREAFKLRGENARLNFPELFLNKNKTDPTIDDSSAAPQGLNMQD
SGSEILPPPVPPPPSLPQQENPEEDSGKGSSEGTESSETQGGAGVGGPELSELVWGDMAEA
WLNAIPAGWGPGSPVWDDLDSSNNLLLPSNLPFANRIDLFKHLQSLPRPPEVLPVQVPLRA
LKVCLAVNFRTPD
>Actinidia31668.t1
MMTTTDELLALELIRQHLLGDFTSTEAFITNLNFSPTASSDNSDLYLSPAHSNSFFSDQIYSD
SPTSTFDYLNPEPSFSEFESKPEIGSLMVSVPKNRGYTRSGQRESGNKSEKRRHYRGVRMR
PWGKFAARFGNPTRKGSRMRGSKAILNFPLEAGKSDQPAGNGRKRRREDGA
>Actinidia10308.t1
MLSRWCIYGVVPTHARDHSAQKANLGQLQEWQLQWTYGVAYHFNQIQSPSGVPTSYPD
GCSTSTTQFFSQGFSTHDPLGLEQPGSVGLNHLTQAQIHFQQPSWPKQHQSLIFLSPKPVP
MKQAGYPTKPTKLYRGVRQRHWEMVAEIRLPKNRTRLWLGTFDTAEEAALAYDKAAYK
LRGDYARLNFPNLRHSGSHIGGDFGEYKPLHSAVDAKLHAICQSLAQGKSLDAKKRSPAS
KKRPSVAAPPPPPPPPAESEVDHSTSNSGSPVTSESYGSGGSSPMSHLAFPDFTEEDPAWDA
SLMLQKYPSYEIDWASL
**>AT5G05410.1**
MAVYDQSGDRNRTQIDTSRKRKSRSRGDGTTVAERLKRWKEYNETVEEVSTKKRKVPAK
GSKKGCMKGKGGPENSRCSFRGVRQRIWGKWVAEIREPNRGSRLWLGTFPTAQEAASAY
DEAAKAMYGPLARLNFPRSDASEVTSTSSQSEVCTVETPGCVHVKTEDPDCESKPFSGGV
EPMYCLENGAEEMKRGVKADKHWLSEFEHNYWSDILKEKEKQKEQGIVETCQQQQQDS
LSVADYGWPNDVDQSHLDSSDMFDVDELLRDLNGDDVFAGLNQDRYPGNSVANGSYRP
ESQQSGFDPLQSLNYGIPPFQLEGKDGNGFFDDLSYLDLEN*

>AT3G11020.1

MAVYEQTGTEQPKKRKSRARAGGLTVADRLKKWKEYNEIVEASAVKEGEKPKRKVPAK

GSKKGCMKGKGGPDNSHCSFRGVRQRIWGKWVAEIREPKIGTRLWLGTFPTAEKAASAY

DEAATAMYGSLARLNFPQSVGSEFTSTSSQSEVCTVENKAVVCGDVCVKHEDTDCESNPF

SQILDVREESCGTRPDSCTVGHQDMNSSLNYDLLLEFEQQYWGQVLQEKEKPKQEEEEIQ

QQQQEQQQQQLQPDLLTVADYGWPWSNDIVNDQTSWDPNECFDINELLGDLNEPGPHQS

QDQNHVNSGSYDLHPLHLEPHDGHEFNGLSSLDI*

>AT5G18450.1

MEEEQPPAKKRNMGRSRKGCMKGKGGPENATCTFRGVRQRTWGKWVAEIREPNRGTRL

WLGTFNTSVEAAMAYDEAAKKLYGHEAKLNLVHPQQQQQVVVNRNLSFSGHGSGSWAY

NKKLDMVHGLDLGLGQASCSRGSCSERSSFLQEDDDHSHNRCSSSSGSNLCWLLPKQSD

SQDQETVNATTSYGGEGGGGSTLTFSTNLKPKNLMSQNYGLYNGAWSRFLVGQEKKTEH

DVSSSCGSSDNKESMLVPSCGGERMHRPELEERTGYLEMDDLLEIDDLGLLIGKNGDFKN

WCCEEFQHPWNWF*

>AT2G40340.6

MKTSSCSYFGVITFRKRKSRGTRDVAEILRQWREYNEQIEAESCIDGGGPKSIRKPPPKGSR

KGCMKGKGGPENGICDYRGVRQRRWGKWVAEIREPDGGARLWLGTFSSSYEAALAYDE

AAKAIYGQSARLNLPEITNRSSSTAATATVSGSVTAFSDESEVCAREDTNASSGFGQVKLE

DCSDEYVLLDSSQCIKEELKGKEEVREEHNLAVGFGIGQDSKRETLDAWLMGNGNEQEPL

EFGVDETFDINELLGILNDNNVSGQETMQYQVDRHPNFSYQTQFPNSNLLGSLNPMEIAQ

PGVDYGCPYVQPSDMENYGIDLDHRRFNDLDIQDLDFGGDKDVHGST*

>AT2G38340.1

MEKEDNGSKQSSSASVVSSRRRRRVVEPVEATLQRWEEEGLARARRVQAKGSKKGCMR

GKGGPENPVCRFRGVRQRVWGKWVAEIREPVSHRGANSSRSKRLWLGTFATAAEAALAY

DRAASVMYGPYARLNFPEDLGGGRKKDEEAESSGGYWLETNKAGNGVIETEGGKDYVV

YNEDAIELGHDKTQNPMTDNEIVNPAVKSEEGYSYDRFKLDNGLLYNEPQSSSYHQGGGF

DSYFEYFRF*

>AT5G67190.1

MEGGGVADVAVPGTRKRDRPYKGIRMRKWGKWVAEIREPNKRSRLWLGSYSTPEAAAR

AYDTAVFYLRGPTARLNFPELLPGEKFSDEDMSAATIRKKATEVGAQVDALGTAVQNNRH

RVFGQNRDSDVDNKNFHRNYQNGEREEEEEDEDDKRLRSGGRLLDRVDLNKLPDPESSD

EEWESKH*

>AT2G23340.1

METEAAVTATVTAATMGIGTRKRDLKPYKGIRMRKWGKWVAEIREPNKRSRIWLGSYATP

EAAARAYDTAVFYLRGPSARLNFPELLAGLTVSNGGGRGGDLSAAYIRRKAAEVGAQVD

ALGATVVVNTGGENRGDYEKIENCRKSGNGSLERVDLNKLPDPENSDGDDDECVKRR*

>AT4G06746.1

MVIQYKRKQEFPMVKEGMVMTEKPKRNLISSNEKRYKGIRMRKWGKWVAEIREPNKRS

RIWLGSYKTAVAAARAYDTAVFYLRGPSARLNFPEEVFKDGNGGEGLGGDMSPTLIRKKA

AEVGARVDAELRLENRMVENLDMNKLPEAYGL*

>AT4G36900.1

METATEVATVVSTPAVTVAAVATRKRDKPYKGIRMRKWGKWVAEIREPNKRSRIWLGSYS

TPEAAARAYDTAVFYLRGPSARLNFPELLAGVTVTGGGGGGVNGGGDMSAAYIRRKAAE

VGAQVDALEAAGAGGNRHHHHHQHQRGNHDYVDNHSDYRINDDLMECSSKEGFKRCN

GSLERVDLNKLPDPETSDDD*

>AT1G21910.1

MVKQERKIQTSSTKKEMPLSSSPSSSSSSSSSSSSSSCKNKNKKSKIKKYKGVRMRSWGSW

VSEIRAPNQKTRIWLGSYSTAEAAARAYDVALLCLKGPQANLNFPTSSSSHHLLDNLLDEN

TLLSPKSIQRVAAQAANSFNHFAPTSSAVSSPSDHDHHHDDGMQSLMGSFVDNHVSLMDS

TSSWYDDHNGMFLFDNGAPFNYSPQLNSTTMLDEYFYEDADIPLWSFN*

>AT3G50260.1

MDAGVAVKADVAVKMKRERPFKGIRMRKWGKWVAEIREPNKRSRLWLGSYSTPEAAAR

AYDTAVFYLRGPTATLNFPELLPCTSAEDMSAATIRKKATEVGAQVDAIGATVVQNNKRRR

VFSQKRDFGGGLLELVDLNKLPDPENLDDDLVGK*

>AT4G25490.1

MNSFSAFSEMFGSDYEPQGGDYCPTLATSCPKKPAGRKKFRETRHPIYRGVRQRNSGKW

VSEVREPNKKTRIWLGTFQTAEMAARAHDVAALALRGRSACLNFADSAWRLRIPESTCAK

DIQKAAAEAALAFQDETCDTTTTNHGLDMEETMVEAIYTPEQSEGAFYMDEETMFGMPT

LLDNMAEGMLLPPPSVQWNHNYDGEGDGDVSLWSY*

>AT4G25480.1

MNSFSAFSEMFGSDYESSVSSGGDYIPTLASSCPKKPAGRKKFRETRHPIYRGVRRRNSGK

WVCEVREPNKKTRIWLGTFQTAEMAARAHDVAALALRGRSACLNFADSAWRLRIPESTC

AKDIQKAAAEAALAFQDEMCDATTDHGFDMEETLVEAIYTAEQSENAFYMHDEAMFEM

PSLLANMAEGMLLPLPSVQWNHNHEVDGDDDDVSLWSY*

>AT5G51990.1

MNPFYSTFPDSFLSISDHRSPVSDSSECSPKLASSCPKKRAGRKKFRETRHPIYRGVRQRNS

GKWVCEVREPNKKSRIWLGTFPTVEMAARAHDVAALALRGRSACLNFADSAWRLRIPET

TCPKEIQKAASEAAMAFQNETTTEGSKTAAEAEEAAGEGVREGERRAEEQNGGVFYMDD

EALLGMPNFFENMAEGMLLPPPEVGWNHNDFDGVGDVSLWSFDE*

>AT4G25470.1

MNSFSAFSEMFGSDYESPVSSGGDYSPKLATSCPKKPAGRKKFRETRHPIYRGVRQRNSG
KWVCELREPNKKTRIWLGTFQTAEMAARAHDVAAIALRGRSACLNFADSAWRLRIPESTC
AKEIQKAAAEAALNFQDEMCHMTTDAHGLDMEETLVEAIYTPEQSQDAFYMDEEAMLG
MSSLLDNMAEGMLLPSPSVQWNYNFDVEGDDDVSLWSY*
>AT1G75490.1
MSSIEPKVMMVGANKKQRTVQASSRKGCMRGKGGPDNASCTYKGVRQRTWGKWVAEI
REPNRGARLWLGTFDTSREAALAYDSAARKLYGPEAHLNLPESLRSYPKTASSPASQTTPS
SNTGGKSSSDSESPCSSNEMSSCGRVTEEISWEHINVDLPVMDDSSIWEEATMSLGFPWVH
EGDNDISRFDTCISGGYSNWDSFHSPL*
>AT2G40350.2
MPSASEIVDRKRKSRGTRDVAEILRKWREYNEQTEADSCIDGGGSKPIRKAPPKRSRKGC
MKGKGGPENGICDYTGVRQRTWGKWVAEIREPGRGAKLWLGTFSSSYEAALAYDEASK
AIYGQSARLNLPLLPLCQARLLHFLMNLKFVHVRIQMQDLVLVRSD*
>AT3G57600.1
MEKSSSMKQWKKGPARGKGGPQNALCQYRGVRQRTWGKWVAEIREPKKRARLWLGSF
ATAEEAAMAYDEAALKLYGHDAYLNLPHLQRNTRPSLSNSQRFKWVPSRKFISMFPSCGM
LNVNAQPSVHIIQQRLEELKKTGLLSQSYSSSSSSTESKTNTSFLDEKTSKGETDNMFEGG
DQKKPEIDLTEFLQQLGILKDENEAEPSEVAECHSPPPWNEQEETGSPFRTENFSWDTLIEM
PRSETTTMQFDSSNFGSYDFEDDVSFPSIWDYYGSLD*
>AT1G22190.1
MTTSMDFYSNKTFQQSDPFGGELMEALLPFIKSPSNDSSAFAFSLPAPISYGSDLHSFSHHL
SPKPVSMKQTGTSAAKPTKLYRGVRQRHWGKWVAEIRLPRNRTRLWLGTFDTAEEAALA
YDKAAYKLRGDFARLNFPDLRHNDEYQPLQSSVDAKLEAICQNLAETTQKQVRSTKKSSS
RKRSSTVAVKLPEEDYSSAGSSPLLTESYGSGGSSSPLSELTFGDTEEEIQPPWNENALEKYP
SYEIDWDSILQCSSLVN*
>AT4G16750.1
MQDSSSHESQRNLRSPVPEKTGKSSKTKNEQKGVSKQPNFRGVRMRQWGKWVSEIREPR
KKSRIWLGTFSTPEMAARAHDVAALAIKGGSAHLNFPELAYHLPRPASADPKDIQEAAAA
AAAVDWKAPESPSSTVTSSPVADDAFSDLPDLLLDVNDHNKNDGFWDSFPYEDPFFLENY
>AT4G32800.1
MADSSSDKEKKENNKQPVYRGVRMRSWGKWVSEIREPRKKSRIWLGTFPTAEMAMRAH
DVAAMSIKGTSAILNFPELSKLLPRPVSLSPRDVRAAATKAALMDFDTTAFRSDTETSETTT
SNKMSESSESNETVSFSSSSWSSVTSIEESTVSDDLDEIVKLPSLGTSLNESNEFVIFDSLEDL
VYMPRWLSGTEEEVFTYNNNDSSLNYSSVFESWKHFP*
>AT5G11590.1

MAEEYYSLRSERVTQLLVPNSESDSVSDKSKAEQSEKKTKRGRDSGKHPVYRGVRMRN
WGKWVSEIREPRKKSRIWLGTFPTPEMAARAHDVAALSIKGTAAILNFPELADSFPRPVSL
SPRDIQTAALKAAHMEPTTSFSSSTSSSSSLSSTSSLESLVLVMDLSRTESEELGEIVELPSLG
ASYDVDSANLGNEFVFYDSVDYCLYPPPWGQSSEDNYGHGISPNFGHGLSWDL*
>AT5G52020.1
MSNNNNSPTTVNQETTTSREVSITLPTDQSPQTSPGSSSSPSPRPSGGSPARRTATGLSGKHS
IFRGIRLRNGKWVSEIREPRKTTRIWLGTYPVPEMAAAAYDVAALALKGPDAVLNFPGLA
LTYVAPVSNSAADIRAAASRAAEMKQPDQGGDEKVLEPVQPGKEEELEEVSCNSCSLEFM
DEEAMLNMPTLLTEMAEGMLMSPPRMMIHPTMEDDSPENHEGDNLWSYK*
>AT1G12630.1
MNSSMASAGLGSRRKDPVYRGIRCRSGKWVSEIREPRKTTRIWLGTYPMAEMAAAAYDV
AAMALKGREAVLNFPGSVGSYPVPESTSAADIRAAAAAAAAMKGCEEGEEEKKAKEKKS
SSSKSRARECHVDNDVGSSSWCGTEFMDEEEVLNMPNLLANMAEGMMVAPPSWMGSRP
SDDSPENSNDEDLWGY*
>AT1G77200.1
MTESSIISVKQSSPVPEEEDHHHHQQDSHRTNTKKRVRSDPGYRGVRMRTWGKWVSEIRE
PRKKSRIWLGTFSTPEMAARAHDAAALTIKGTSAVLNFPELATYLPRPASSSPRDVQAAAA
VAAAMDFSPSSSSLVVSDPTTVIAPAETQLSSSSYSTCTSSSLSPSSEEAASTAEELSEIVELP
SLETSYDESLSEFVYVDSAYPPSSPWYINNCYSFYYHSDENGISMAEPFDSSNFGPLFP*
>AT1G01250.1
MSPQRMKLSSPPVTNNEPTATASAVKSCGGGGKETSSSTTRHPVYHGVRKRRWGKWVSEI
REPRKKSRIWLGSFPVPEMAAKAYDVAAFCLKGRKAQLNFPEEIEDLPRPSTCTPRDIQVA
AAKAANAVKIIKMGDDDVAGIDDGDDFWEGIELPELMMSGGGWSPEPFVAGDDATWLV
DGDLYQYQFMACL*
>AT2G25820.1
MVDSHGSDTECSSKKKKEKTKEKGVYRGARMRSWGKWVSEIREPRKKSRIWLGTFPTAE
MAARAHDVAALSIKGSSAILNFPELADFLPRPVSLSQQDIQAAAAEAALMDFKTVPFHLQ
DDSTPLQTRCDTEKIEKWSSSSSSASSSSSSSSSSSSSMLSGELGDIVELPSLENNVKYDCAL
YDSLEGLVSMPPWLDATENDFRYGDDSVLLDPCLKESFLWNYE*
>AT2G44940.1
MARQINIESSVSQVTFISSAIPAVSSSSSITASASLSSSPTTSSSSSSSTNSNFIEEDNSKRKASR
RSLSSLVSVEDDDDQNGGGGKRRKTNGGDKHPTYRGVRMRSWGKWVSEIREPRKKSRI
WLGTYPTAEMAARAHDVAALAIKGTTAYLNFPKLAGELPRPVTNSPKDIQAAASLAAVN
WQDSVNDVSNSEVAEIVEAEPSRAVVAQLFSSDTSTTTTTQSQEYSEASCASTSACTDKDS
EEEKLFDLPDLFTDENEMMIRNDAFCYYSSTWQLCGADAGFRLEEPFFLSE*

>AT3G16280.2
MSHIHTKRRSKYQTRVQKMTSLNSSASPTSSSSDQSDATTTTSTHLSEEEAPPRNNNTRKR
RRDSSSASSSSSMQHPVYRGVRMRSWGKWVSEIRQPRKKTRIWLGTFVTADMAARAHD
VAALTIKGSSAVLNFPELASLFPRPASSSPHDIQTAAAEAAAMVVEEKLLEKDEAPEAPPSS
ESSYVAAESEDEERLEKIVELPNIEEGSYDESVTSRADLAYSEPFDCWVYPPVMDFYEEISE
FNFVELWSFNH*
>AT3G60490.1
MGKQINIESSATHHQDNIVSVITATISSSSVVTSSSDSWSTSKRSLVQDNDSGGKRRKSNVS
DDNKNPTSYRGVRMRSWGKWVSEIREPRKKSRIWLGTYPTAEMAARAHDVAALAIKGN
SGFLNFPELSGLLPRPVSCSPKDIQAAATKAAEATTWHKPVIDKKLADELSHSELLSTAQSS
TSSSFVFSSDTSETSSTDKESNEETVFDLPDLFTDGLMNPNDAFCLCNGTFTWQLYGEEDV
GFRFEEPFNWQND*
>AT4G39780.1
MAAIDMFNSNTDPFQEELMKALQPYTTNTDSSSPTYSNTVFGFNQTTSLGLNQLTPYQIH
QIQNQLNQRRNIISPNLAPKPVPMKNMTAQKLYRGVRQRHWGKWVAEIRLPKNRTRLWL
GTFDTAEEAAMAYDLAAYKLRGEFARLNFPQFRHEDGYYGGGSCFNPLHSSVDAKLQEIC
QSLRKTEDIDLPCSETELFPPKTEYQESEYGFLRSDENSFSDESHVESSSPESGITTFLDFSDS
GFDEIGSFGLEKFPSVEIDWDAISKLSES*
>AT5G65130.2
MAMALNMNAYVDEFMEALEPFMKVTSSSSTSNSSNPKPLTPNFIPNNDQVLPVSNQTGPI
GLNQLTPTQILQIQTELHLRQNQSRRRAGSHLLTAKPTSMKKIDVATKPVKLYRGVRQRQ
WGKWVAEIRLPKNRTRLWLGTFETAQEAALAYDQAAHKIRGDNARLNFPDIVRQGHYKQ
ILSPSINAKIESICNSSDLPLPQIEKQNKTEEVLSGFSKPEKEPEFGEIYGCGYSGSSPESDITL
LDFSSDCVKEDESFLMGLHKYPSLEIDWDAIEKLF*
>AT1G64380.1
MEESNDIFQNNFSPKISEIRASLSQIILAGGPNTLDSIFSLLTPSSVESATTSFNTHNPPPPPQL
GSSVYLRQRDIIEKFHLQNRAISTPHPPLFSSTYDHHQTSELMLQAAAGSPAAAFAAALAA
GRVTKKKKLYRGVRQRHWGKWVAEIRLPQNRMRVWLGTYDTAEAAAYAYDRAAYKLR
GEYARLNFPNLKDPSELLGLGDSSKLIALKNAVDGKIQSICQRVRKERAKKSVKVSKNSSA
TADSSCLSSPEILSSSPVTTTTTAVTSEDSYWVSPMGLCNSENSSPVSVSVPSEVPATAEEEA
MMGVDTDGFLLARMPSFDPELIWEVLAN*
>AT2G22200.1
METASLSFPVPNTSFGVNKSMPLGLNQLTPYQIHQIQNQLNHRRSTISNLSPNRIRMKNLTP
STSKTKNLYRGVRQRHWGKWVAEIRLPKNRTRLWLGTFETAEKAALAYDQAAFQLRGDI
AKLNFPNLIHEDMNPLPSSVDTKLQAICKSLRKTEEICSVSDQTKEYSVYSVSDKTELFLPK

AELFLPKREHLETNELSNESPRSDETSLLDESQAEYSSSDKTFLDFSDTEFEEIGSFGLRKFP
SVEIDWDAISKLANS*
>AT4G28140.1
MDFDEELNLCITKGKNVDHSFGGEASSTSPRSMKKMKSPSRPKPYFQSSSSPYSLEAFPFS
LDPTLQNQQQQLGSYVPVLEQRQDPTMQGQKQMISFSPQQQQQQQYMAQYWSDTLN
LSPRGRMMMMMSQEAVQPYIATKLYRGVRQRQWGKWVAEIRKPRSRARLWLGTFDTAE
EAAMAYDRQAFKLRGHSATLNFPEHFVNKESELHDSNSSDQKEPETPQPSEVNLESKELP
VIDVGREEGMAEAWYNAITSGWGPESPLWDDLDSSHQFSSESSSSSPLSCPMRPFF*
>AT4G13620.1
MITPIHTQHSLILVYINIYSPPILSKLRTGFILWTNTQKTNKKRNMEDQFPKIETSFMHDKLL
SSGIYGFLSSSTPPQLLGVPIFLEGMKSPLLPASSTPSYFVSPHDHELTSSIHPSPVASVPWNF
LESFPQSQHPDHHPSKPPNLTLFLKEPKLLELSQSESNMSPYHKYIPNSFYQSDQNRNEWV
EINKTLTNYPSKGFGNYWLSTTKTQPMKSKTRKVVQTTTPTKLYRGVRQRHWGKWVAEI
RLPRNRTRVWLGTFETAEQAAMAYDTAAYILRGEFAHLNFPDLKHQLKSGSLRCMIASLL
ESKIQQISSSQVSNSPSPPPPKVGTPEQKNHHMKMESGEDVMMKKQKSHKEVMEGDGVQ
LSRMPSLDMDLIWDALSFPHSS*
>AT1G19210.1
MEGSSSSMQSKYKGVRKRKWGKWVSEIRLPNSRERIWLGSYDTPEKAARAFDAALYCLR
GNNAKFNFPDNPPVISGGRNLSRSEIREAAARFANSAEDDSSGGAGYEIRQESASTSMDVD
SEFLSMLPTVGSGNFASEFGLFPGFDDFSDEYSGDRFREQLSPTQDYYQLGEETYADGSMF
LWNF*
>AT1G77640.1
MVKQELKIQVTTSSSSLSHSSSSSSSSTSALRHQSCKNKIKKYKGVRMRSWGSWVTEIRAP
NQKTRIWLGSYSTAEAAARAYDAALLCLKGPKANLNFPNITTTSPFLMNIDEKTLLSPKSI
QKVAAQAANSSSDHFTPPSDENDHDHDDGLDHHPSASSSAASSPPDDDHHNDDDGDLVS
LMESFVDYNEHVSLMDPSLYEFGHNEIFFTNGDPFDYSPQLHSSEATMDDFYDDVDIPLW
SFS*
>AT1G71520.1
MDSRDTGETDQSKYKGIRRRKWGKWVSEIRVPGTRQRLWLGSFSTAEGAAVAHDVAFYC
LHRPSSLDDESFNFPHLLTTSLASNISPKSIQKAASDAGMAVDAGFHGAVSGSGGCEERSS
MANMEEEDKLSISVYDYLEDDLV*
>AT1G46768.1
MEREQEESTMRKRRQPPQEEVPNHVATRKPYRGIRRRKWGKWVAEIREPNKRSRLWLGS
YTTDIAAARAYDVAVFYLRGPSARLNFPDLLLQEEDHLSAATTADMPAALIREKAAEVGA
RVDALLASAAPSMAHSTPPVIKPDLNQIPESGDI*

>AT1G33760.1

MENTYVGQRDYRFNVNQLSYRGIRRRKWGKWVSEIREPGKKTRIWLGSYETAEMAAAA

YDAAALHLRGRGTNLNFPELVDSFPRPESSSSEHIQAAAQDAALMFKPGRLSEPALESGQG

LSRVGLSPDQIQAINESPLDSPRMGWMQDLEVADYEELYGQFFGQHDRDEFFEMQQFQSI

WNSNN*

>AT1G78080.1

MAAAMNLYTCSRSFQDSGGELMDALVPFIKSVSDSPSSSSAASASAFLHPSAFSLPPLPGY

YPDSTFLTQPFSYGSDLQQTGSLIGLNNLSSSQIHQIQSQIHHPLPPTHHNNNNSFSNLLSPK

PLLMKQSGVAGSCFAYGSGVPSKPTKLYRGVRQRHWGKWVAEIRLPRNRTRLWLGTFDT

AEEAALAYDKAAYKLRGDFARLNFPNLRHNGSHIGGDFGEYKPLHSSVDAKLEAICKSM

AETQKQDKSTKSSKKREKKVSSPDLSEKVKAEENSVSIGGSPPVTEFEESTAGSSPLSDLTF

ADPEEPPQWNETFSLEKYPSYEIDWDSILA*

>AT2G35700.1

MERDDCRRFQDSPAQTTERRVKYKPKKKRAKDDDDEKVVSKHPNFRGVRMRQWGKWV

SEIREPKKKSRIWLGTFSTAEMAARAHDVAALAIKGGSAHLNFPELAYHLPRPASADPKDI

QAAAAAAAAAVAIDMDVETSSPSPSPTVTETSSPAMIALSDDAFSDLPDLLLNVNHNIDGF

WDSFPYEEPFLSQSY*

>AT5G21960.1

MDASPKYTGVRKRKWGKWVAEIRLPNSRDRIWLGSFDSAEKAARAFDAALYCLRGPGA

RFNFPDNPPEIPGGRSLTPQQIQVVASRFACEEELLPPEQHHPSPPRGDHNTEEEVIISARGEI

NSGSGGPTLGQVGEDNNNEGNSNDTSSYWPLIWEEENFVGPPNSDHEFGFFTDDSTNLYF

PTQQQQQHQLSSDFYYDGACEDDFSHYNINLWNF*

>AT4G31060.1

MPPSPPKSPFISSSLKGAHEDRKFKCYRGVRKRSWGKWVSEIRVPKTGRRIWLGSYDAPE

KAARAYDAALFCIRGEKGVYNFPTDKKPQLPEGSVRPLSKLDIQTIATNYASSVVHVPSHA

TTLPATTQVPSEVPASSDVSASTEITEMVDEYYLPTDATAESIFSVEDLQLDSFLMMDIDWI

NNLI*

>AT1G71450.1

MAGLRNSGNSDKAQNDGKGVPSAYRGVRKRKWGKWVSEIREPGTKNRIWLGSFETPEM

AATAYDVAAFHFRGREARLNFPELASSLPRPADSSSDSIRMAVHEATLCRTTEGTESAMQV

DSSSSSNVAPTMVRLSPREIQAINESTLGSPTTMMHSTYDPMEFANDVEMNAWETYQSDF

LWDP*

>AT1G74930.1

MVKQAMKEEEKKRNTAMQSKYKGVRKRKWGKWVSEIRLPHSRERIWLGSYDTPEKAA

RAFDAAQFCLRGGDANFNFPNNPPSISVEKSLTPPEIQEAAARFANTFQDIVKGEEESGLVP

GSEIRPESPSTSASVATSTVDYDFSFLDLLPMNFGFDSFSDDFSGFSGGDRFTEILPIEDYGG
ESLLDESLILWDF*
>AT1G22810.1
MDYRESTGESQSKYKGIRRRKWGKWVSEIRVPGTRDRLWLGSFSTAEGAAVAHDVAFFCL
HQPDSLESLNFPHLLNPSLVSRTSPRSIQQAASNAGMAIDAGIVHSTSVNSGCGDTTTYYE
NGADQVEPLNISVYDYLGGHDHV*
>AT5G25810.1
MIASESTKSWEASAVRQENEEEKKKPVKDSGKHPVYRGVRKRNWGKWVSEIREPRKKSR
IWLGTFPSPEMAARAHDVAALSIKGASAILNFPDLAGSFPRPSSLSPRDIQVAALKAAHME
TSQSFSSSSSLTFSSSQSSSSLESLVSSSATGSEELGEIVELPSLGSSYDGLTQLGNEFIFSDSA
DLWPYPPQWSEGDYQMIPASLSQDWDLQGLYNY*
>AT1G44830.1
MVKTLQKTPKRMSSPSSSSSSSSSTSSSSIRMKKYKGVRMRSWGSWVSEIRAPNQKTRIW
LGSYSTAEAAARAYDAALLCLKGSSANNLNFPEISTSLYHIINNGDNNNDMSPKSIQRVAA
AAAAANTDPSSSSVSTSSPLLSSPSEDLYDVVSMSQYDQQVSLSESSSWYNCFDGDDQFM
FINGVSAPYLTTSLSDDFFEEGDIRLWNFC*
>AT2G36450.1
MQGTSKDNGGRHPLYRGVRQRKNSNKWVSEIREPRKPNRIWLGTFSTPEMAAIAYDVAA
LALKGSQAELNFPNSVSSLPAPTSMSPADIQAAAASAAAAFGAARDAIVMANNNSQTSGV
ACMNSSYDNTNMNGFMDEDLVFDMPNVLMNMAEGMLLSPPRPTVFDAAYDADGFPGG
DDYLWNFP*
>AT2G40220.1
MDPLASQHQHNHLEDNNQTLTHNNPQSDSTTDSSTSSAQRKRKGKGGPDNSKFRYRGVR
QRSWGKWVAEIREPRKRTRKWLGTFATAEDAARAYDRAAVYLYGSRAQLNLTPSSPSSVS
SSSSSVSAASSPSTSSSSTQTLRPLLPRPAAATVGGGANFGPYGIPFNNNIFLNGGTSMLCPS
YGFFPQQQQQQNQMVQMGQFQHQQYQNLHSNTNNNKISDIELTDVPVTNSTSFHHEVAL
GQEQGGSGCNNNSSMEDLNSLAGSVGSSLSITHPPPLVDPVCSMGLDPGYMVGDGSSTIW
PFGGEEEYSHNWGSIWDFIDPILGEFY*
>AT1G63030.1
MENDDITVAEMKPKKRAGRRIFKETRHPIYRGVRRRDGDKWVCEVREPIHQRRVWLGTY
PTADMAARAHDVAVLALRGRSACLNFSDSAWRLPVPASTDPDTIRRTAAEAAEMFRPPEF
STGITVLPSASEFDTSDEGVAGMMMRLAEEPLMSPPRSYIDMNTSVYVDEEMCYEDLSLW
SY*
>AT1G36060.1
MADLFGGGHGGELMEALQPFYKSASTSASNPAFASSNDAFASAPNDLFSSSSYYNPHASL

FPSHSTTSYPDIYSGSMTYPSSFGSDLQQPENYQSQFHYQNTITYTHQDNNTCMLNFIEPSQ
PGFMTQPGPSSGSVSKPAKLYRGVRQRHWGKWVAEIRLPRNRTRLWLGTFDTAEEAALA
YDRAAFKLRGDSARLNFPALRYQTGSSPSDTGEYGPIQAAVDAKLEAILAEPKNQPGKTER
TSRKRAKAAASSAEQPSAPQQHSGSGESDGSGSPTSDVMVQEMCQEPEMPWNENFMLG
KCPSYEIDWASILS*

>AT1G12610.1

MNNDDIILAEMRPKKRAGRRVFKETRHPVYRGIRRRNGDKWVCEVREPTHQRRIWLGTY
PTADMAARAHDVAVLALRGRSACLNFADSAWRLPVPESNDPDVIRRVAAEAAEMFRPVD
LESGITVLPCAGDDVDLGFGSGSGSGSGSEERNSSSYGFGDYEEVSTTMMRLAEGPLMSP
PRSYMEDMTPTNVYTEEEMCYEDMSLWSYRY*

>AT1G63040

MADPNNPITEPKAIIQSSTSSSVTIVPVPTCGDSLSDSATCENPCPLDTITTTTTTVCFAAPSS
TASGNDINTLMATDTDISRRKKNPVYRGIRCRSGKWVSEIREPKKTTRVWLGTYPTPEMA
AAAYDVAALALKGGDTLLNFPDSLGSYPIPLSSSAAHIRCAAAAAAATRGAAGAAVKVGQ
KKEDKVYDTAESSTMGFVDEEELLNMPGLLADMAKGMMVAPPWMGSPPSDDSPENSDG
ESLWSY
