# Supplementary material for: Genome-Wide Identification of DREB Gene Family in Kiwifruit and Functional Characterization of Exogenous 5-ALA-Mediated Cold Tolerance via ROS Scavenging and Hormonal Signaling
Source: Plants (Basel). 2025 Aug 17;14(16):2560. doi: 10.3390/plants14162560 (PMC12389587; doi:10.3390/plants14162560)
Supplement: Supplementary file 1 [file plants-14-02560-s001.zip › Annexed table S3 Description of cis-acting elements of DREB gene promoters in kiwifruit.pdf]

Annexed table S3 Description of cis-acting elements of DREB gene promoters in kiwifruit

Categorization	Designation	Description in English	Chinese Description
Plant growth and development	CAT-box	meristem expression	分生组织表达
	CCAAT-box	MYBHv1 binding site	MYBHv1 结合位点
	circadian	circadian control	昼夜节律控制
	GCN4_motif	endosperm expression	胚乳表达
	O2-site	zein metabolism regulation	玉米蛋白代谢调节
Phytohormone responses	ABRE	abscisic acid responsiveness	脱落酸反应性
	CGTCA-motif	MeJA-responsiveness	MeJA 反应性
	ERE		
	GARE-motif	gibberellin-responsive element	赤霉素反应元件
	MBSI	flavonoid biosynthetic genes regulation	黄酮类生物合成基因调控
	P-box	gibberellin-responsive element	赤霉素反应元件
	TATC-box	gibberellin-responsiveness	赤霉素反应性
	TCA-element	salicylic acid responsiveness	水杨酸反应性
	TGACG-motif	MeJA-responsiveness	MeJA 反应性
	TGA-element	auxin-responsive element	辅助因子反应元件
Abiotic stresses and stresses	ARE	anaerobic induction	厌氧诱导
	CCAAT-box	MYBHv1 binding site	MYBHv1 结合位点
	DRE		
	GC-motif	anoxic specific inducibility	缺氧特异诱导性
	LTR	low-temperature responsiveness	低温反应性
	MBS	MYB binding site involved in drought-inducibility	参与干旱诱导的 MYB 结合位点
	TC-rich repeats	defense and stress responsiveness	防御和胁迫响应性
Light responsiveness	GATA-motif	part of a light responsive element	光响应元件的一部分
	GT1-motif	light responsive element	光响应元件
	MRE	MYB binding site involved in light responsiveness	涉及光响应性的 MYB 结合位点
	Sp1	light responsive element	光响应元件
