# Supplementary material for: Genome-Wide Identification of DREB Gene Family in Kiwifruit and Functional Characterization of Exogenous 5-ALA-Mediated Cold Tolerance via ROS Scavenging and Hormonal Signaling
Source: Plants (Basel). 2025 Aug 17;14(16):2560. doi: 10.3390/plants14162560 (PMC12389587; doi:10.3390/plants14162560)
Supplement: Supplementary file 1 [file plants-14-02560-s001.zip › Annexed Table S4 Kiwifruit DREB gene family fragment repeat genes.pdf]

Annexed Table S4 Kiwifruit DREB gene family fragment repeat genes

Chromosome name	GeneID	gene location		Chromosome name	GeneID	gene location	
Lachesis_group10	Actinidia13662.t1	6449442	6451476	Lachesis_group14	Actinidia14886.t1	10580516	10582849
Lachesis_group10		6458780	6463649	Lachesis_group14		10604089	10607352
Lachesis_group10	Actinidia19931.t1	7843251	7844416	Lachesis_group15		1381256	1381788
Lachesis_group23	Actinidia02046.t1	5284783	5287771	Lachesis_group6	Actinidia14518.t1	17709076	17731261
Lachesis_group11	Actinidia12034.t1	18006805	18010188	Lachesis_group25	Actinidia16472.t1	10603501	10606720
Lachesis_group16	Actinidia33507.t1	14154051	14154793	Lachesis_group22	Actinidia25891.t1	2368463	2377890
Lachesis_group16	Actinidia33507.t1	14154051	14154793	Lachesis_group23	Actinidia02179.t1	7014600	7015896
Lachesis_group16	Actinidia28438.t1	7754718	7758067	Lachesis_group27	Actinidia04754.t1	4966121	4968211
Lachesis_group16	Actinidia27955.t1	17965854	17974945	Lachesis_group4		6134365	6135161
Lachesis_group14		5964322	5971711	Lachesis_group6	Actinidia20193.t1	20495570	20510831
Lachesis_group3		15877134	15883381	Lachesis_group6	Actinidia20193.t1	20495570	20510831
Lachesis_group10	Actinidia01350.t1	4245170	4253556	Lachesis_group12	Actinidia15099.t1	18310468	18315049
Lachesis_group10	Actinidia30547.t1	2216334	2220882	Lachesis_group15	Actinidia22077.t1	4439227	4452210
Lachesis_group10	Actinidia01350.t1	4245170	4253556	Lachesis_group15	Actinidia08136.t1	2794804	2801124
Lachesis_group12	Actinidia15099.t1	18310468	18315049	Lachesis_group15	Actinidia08136.t1	2794804	2801124
Lachesis_group12	Actinidia33993.t1	19989889	19990988	Lachesis_group16	Actinidia09595.t1	424103	425097
Lachesis_group13	Actinidia00898.t1	380048	380854	Lachesis_group24	Actinidia16248.t1	381982	385480
Lachesis_group13	Actinidia06886.t1	1172144	1175330	Lachesis_group24		1379450	1382300
Lachesis_group15	Actinidia25820.t1	19486192	19487498	Lachesis_group9	Actinidia21025.t1	21014591	21016021
Lachesis_group15	Actinidia09555.t1	8109080	8113329	Lachesis_group9	**Actinidia25785.t2**	19338653	19348013
Lachesis_group19	Actinidia21434.t1	17259979	17261152	Lachesis_group9	Actinidia21025.t1	21014591	21016021
Lachesis_group18	Actinidia30245.t1	16525712	16535313	Lachesis_group8	Actinidia38486.t1	4348785	4349534
Lachesis_group25	Actinidia24561.t1	5442858	5448316	Lachesis_group8	Actinidia28819.t1	1223186	1236031
Lachesis_group22	Actinidia31667.t1	4158404	4159483	Lachesis_group3	Actinidia13612.t1	13404552	13408050
Lachesis_group13	Actinidia39827.t1	16105915	16108753	Lachesis_group14	Actinidia14886.t1	10580516	10582849
Lachesis_group26	Actinidia17778.t1	7586165	7589817	Lachesis_group27		4966121	4968211
Lachesis_group26	Actinidia10308.t1	6851007	6856238	Lachesis_group28		6302577	6303902
Lachesis_group21	Actinidia31772.t1	16356508	16357569	Lachesis_group3	Actinidia13611.t1	13423411	13424398
Lachesis_group22	Actinidia31668.t1	4148197	4148801	Lachesis_group3	Actinidia13611.t1	13423411	13424398
Lachesis_group3		13423411	13424398	Lachesis_group3	Actinidia13611.t1	22276497	22277335
Lachesis_group14	Actinidia14855.t1	10126168	10127267	Lachesis_group15	Actinidia38388.t1	607021	608077
Lachesis_group14	Actinidia14886.t1	10580516	10582849	Lachesis_group4		11464095	11464692
Lachesis_group26	Actinidia17778.t1	7586165	7589817	Lachesis_group7	Actinidia29544.t1	4895953	4898241
Lachesis_group26	Actinidia02611.t1	10489173	10490630	Lachesis_group7	Actinidia29458.t1	6632176	6632753
Lachesis_group27		4966121	4968211	Lachesis_group7	Actinidia29544.t1	4895953	4898241
Lachesis_group27	Actinidia03523.t1	8174891	8176088	Lachesis_group7	Actinidia29390.t1	8179337	8183604
Lachesis_group13		3660887	3662356	Lachesis_group15	Actinidia00814.t3	13236059	13239109
Lachesis_group15	Actinidia25820.t1	19486192	19487498	Lachesis_group19	Actinidia21434.t1	17259979	17261152
Lachesis_group15	Actinidia00814.t3	13236059	13239109	Lachesis_group24	Actinidia37282.t1	3979664	3983887

Lachesis_group26	Actinidia01091.t1	4015819	4016554	Lachesis_group28	Actinidia22649.t1	3659972	3660755
Lachesis_group26	Actinidia10225.t1	4876400	4881334	Lachesis_group28	Actinidia19292.t1	4537786	4542186
Lachesis_group11	Actinidia06078.t1	13980442	13982216	Lachesis_group12	Actinidia31185.t1	16239077	16242554
Lachesis_group11	Actinidia06078.t1	13980442	13982216	Lachesis_group3		4437479	4439778
Lachesis_group21		16335792	16337441	Lachesis_group22	Actinidia31666.t1	4175448	4188522
Lachesis_group22	Actinidia31666.t1	4175448	4188522	Lachesis_group3		13382306	13383848
Lachesis_group22	Actinidia31666.t1	4175448	4188522	Lachesis_group3		22261531	22262874
Lachesis_group1	Actinidia25486.t1	3712482	3717283	Lachesis_group13	Actinidia18264.t1	10822659	10827309
Lachesis_group1	Actinidia25486.t1	3712482	3717283	Lachesis_group2	Actinidia08932.t1	2216248	2223593
Lachesis_group1	Actinidia25486.t1	3712482	3717283	Lachesis_group9		2040888	2045418
Lachesis_group13	Actinidia18264.t1	10822659	10827309	Lachesis_group2	Actinidia08932.t1	2216248	2223593
Lachesis_group13	Actinidia18234.t1	11143268	11143976	Lachesis_group27	Actinidia23090.t1	10375505	10380757
Lachesis_group13	Actinidia18264.t1	10822659	10827309	Lachesis_group9		2040888	2045418
Lachesis_group2	Actinidia08932.t1	2216248	2223593	Lachesis_group9		2040888	2045418
Lachesis_group1	Actinidia25446.t1	3420006	3420710	Lachesis_group13	Actinidia18234.t1	11143268	11143976
Lachesis_group1	Actinidia25446.t1	3420006	3420710	Lachesis_group14	Actinidia14109.t1	771244	772574
Lachesis_group1	Actinidia25446.t1	3420006	3420710	Lachesis_group23	Actinidia22309.t1	1679293	1679909
Lachesis_group1	Actinidia25446.t1	3420006	3420710	Lachesis_group27	Actinidia23090.t1	10375505	10380757
Lachesis_group14	Actinidia14673.t1	2080636	2081430	Lachesis_group14	Actinidia30276.t1	8413081	8414806
Lachesis_group14	Actinidia14109.t1	771244	772574	Lachesis_group18	Actinidia13205.t1	1225203	1225982
Lachesis_group14	Actinidia14673.t1	2080636	2081430	Lachesis_group18	Actinidia13333.t1	2871811	2872639
Lachesis_group14		6124790	6125689	Lachesis_group23	Actinidia22149.t1	3785646	3788269
Lachesis_group14	Actinidia14109.t1	771244	772574	Lachesis_group23	Actinidia22309.t1	1679293	1679909
Lachesis_group14	Actinidia14109.t1	771244	772574	Lachesis_group27	Actinidia23090.t1	10375505	10380757
Lachesis_group14	Actinidia14673.t1	2080636	2081430	Lachesis_group27	Actinidia15738.t1	12052653	12058737
Lachesis_group0	Actinidia20766.t1	22204020	22204891	Lachesis_group21	Actinidia00657.t2	15670523	15673137
Lachesis_group0	Actinidia20766.t1	22204020	22204891	Lachesis_group3	Actinidia00973.t1	21750343	21752664
Lachesis_group0	Actinidia20766.t1	22204020	22204891	Lachesis_group8	Actinidia32541.t1	19151001	19153501
Lachesis_group21	Actinidia00657.t2	15670523	15673137	Lachesis_group3	Actinidia00973.t1	21750343	21752664
Lachesis_group21	Actinidia31771.t1	16339590	16344002	Lachesis_group3	Actinidia01043.t1	22265307	22266886
Lachesis_group21	Actinidia31772.t1	16356508	16357569	Lachesis_group3	Actinidia01044.t1	22276497	22277335
Lachesis_group22	Actinidia31668.t1	4148197	4148801	Lachesis_group3	Actinidia01044.t1	22276497	22277335
Lachesis_group1	Actinidia25518.t1	4027834	4047664	Lachesis_group13	Actinidia18299.t1	10439692	10441058
Lachesis_group1	Actinidia25518.t1	4027834	4047664	Lachesis_group2	Actinidia08910.t1	2395151	2397507
Lachesis_group1	Actinidia25518.t1	4027834	4047664	Lachesis_group9	Actinidia36843.t1	2225474	2227211
Lachesis_group13	Actinidia18299.t1	10439692	10441058	Lachesis_group2	Actinidia08910.t1	2395151	2397507
Lachesis_group13	Actinidia18299.t1	10439692	10441058	Lachesis_group9	Actinidia36843.t1	2225474	2227211
Lachesis_group2	Actinidia08910.t1	2395151	2397507	Lachesis_group9	Actinidia36843.t1	2225474	2227211
Lachesis_group20	Actinidia31506.t1	12614584	12615692	Lachesis_group23	Actinidia02176.t1	6948609	6957579
Lachesis_group21	**Actinidia31862.t1**	17334658	17335761	Lachesis_group3	Actinidia03155.t1	23225306	23226495
Lachesis_group3	Actinidia03155.t1	23225306	23226495	Lachesis_group6	Actinidia08622.t1	8687752	8691964
Lachesis_group2	Actinidia26001.t1	333382	336515	Lachesis_group27		15205211	15207912
Lachesis_group2	Actinidia26001.t1	333382	336515	Lachesis_group9		344758	361509
Lachesis_group11	Actinidia17188.t1	3226743	3233589	Lachesis_group16	Actinidia06476.t1	11870281	11876943

Lachesis_group11	Actinidia06105.t1	14293407	14296203	Lachesis_group21	Actinidia17567.t1	3235908	3237406
Lachesis_group18	Actinidia13333.t1	2871811	2872639	Lachesis_group18	Actinidia20414.t1	14648088	14650236
Lachesis_group18	Actinidia13205.t1	1225203	1225982	Lachesis_group23	Actinidia22309.t1	1679293	1679909
Lachesis_group18	Actinidia13333.t1	2871811	2872639	Lachesis_group27	Actinidia15738.t1	12052653	12058737
Lachesis_group18	Actinidia13205.t1	1225203	1225982	Lachesis_group27	Actinidia23090.t1	10375505	10380757
Lachesis_group26	Actinidia01091.t1	4015819	4016554	Lachesis_group4	Actinidia36471.t1	5807898	5808936
Lachesis_group26	Actinidia15373.t1	2105069	2106464	Lachesis_group7	Actinidia37989.t1	1819366	1831234
Lachesis_group26	Actinidia01091.t1	4015819	4016554	Lachesis_group7	Actinidia38068.t1	2604072	2606298
Lachesis_group27		1136180	1138736	Lachesis_group7	Actinidia37989.t1	1819366	1831234
Lachesis_group28	Actinidia22649.t1	3659972	3660755	Lachesis_group4	Actinidia36471.t1	5807898	5808936
Lachesis_group28	Actinidia22649.t1	3659972	3660755	Lachesis_group7	Actinidia38068.t1	2604072	2606298
Lachesis_group0	Actinidia13113.t2	23978045	23981051	Lachesis_group20	Actinidia05276.t1	9718396	9722700
Lachesis_group0	Actinidia10399.t1	25210761	25224026	Lachesis_group20	Actinidia31286.t1	8650017	8652010
Lachesis_group0	Actinidia13113.t2	23978045	23981051	Lachesis_group22	Actinidia03019.t1	7207873	7212608
Lachesis_group0	Actinidia13113.t2	23978045	23981051	Lachesis_group24	Actinidia37282.t1	3979664	3983887
Lachesis_group23	Actinidia02153.t1	6703757	6705613	Lachesis_group23	Actinidia12663.t1	13668400	13669374
Lachesis_group0	Actinidia16565.t1	2912322	2913796	Lachesis_group17	Actinidia26137.t1	15595719	15599317
Lachesis_group0	Actinidia16565.t1	2912322	2913796	Lachesis_group20	Actinidia05650.t1	2647691	2648615
Lachesis_group0	Actinidia16565.t1	2912322	2913796	Lachesis_group6	Actinidia07595.t1	3827362	3833687
Lachesis_group17	Actinidia26137.t1	15595719	15599317	Lachesis_group6	Actinidia07595.t1	3827362	3833687
Lachesis_group20	Actinidia05650.t1	2647691	2648615	Lachesis_group6	Actinidia07595.t1	3827362	3833687
Lachesis_group0	Actinidia23663.t1	3185488	3186875	Lachesis_group20	Actinidia05668.t1	2868555	2875730
Lachesis_group0	Actinidia23663.t1	3185488	3186875	Lachesis_group6	Actinidia07617.t1	3567238	3568539
Lachesis_group20	Actinidia05668.t1	2868555	2875730	Lachesis_group6	Actinidia07617.t1	3567238	3568539
Lachesis_group0	Actinidia33182.t1	18816594	18817760	Lachesis_group8	Actinidia35862.t1	17513530	17514681
Lachesis_group17		15872145	15873173	Lachesis_group6	Actinidia07616.t1	3570836	3577350
Lachesis_group17	Actinidia26138.t1	15614581	15617700	Lachesis_group6	Actinidia07597.t1	3813413	3814114
Lachesis_group18	Actinidia08430.t1	5958905	5959914	Lachesis_group5	Actinidia05451.t1	12845631	12846670
Lachesis_group19	Actinidia11051.t1	2876117	2878191	Lachesis_group5	Actinidia09869.t1	2179116	2181229
Lachesis_group20	Actinidia31286.t1	8650017	8652010	Lachesis_group5	Actinidia05451.t1	12845631	12846670
Lachesis_group11	Actinidia12034.t1	18006805	18010188	Lachesis_group4	Actinidia36473.t1	5843031	5845438
Lachesis_group12	Actinidia00441.t1	2630971	2635947	Lachesis_group16	Actinidia27956.t1	17951856	17952677
Lachesis_group12	Actinidia00441.t1	2630971	2635947	Lachesis_group25	Actinidia16494.t1	10292324	10295703
Lachesis_group14	Actinidia14855.t1	10126168	10127267	Lachesis_group4	Actinidia16620.t1	12079475	12081753
Lachesis_group15	Actinidia38388.t1	607021	608077	Lachesis_group4	Actinidia16620.t1	12079475	12081753
Lachesis_group25	Actinidia16472.t1	10603501	10606720	Lachesis_group4	Actinidia36473.t1	5843031	5845438
Lachesis_group13		3660887	3662356	Lachesis_group20	Actinidia05276.t1	9718396	9722700
Lachesis_group20	Actinidia05276.t1	9718396	9722700	Lachesis_group22	Actinidia03019.t1	7207873	7212608
Lachesis_group20	Actinidia05276.t1	9718396	9722700	Lachesis_group24	Actinidia37282.t1	3979664	3983887
Lachesis_group20	Actinidia05276.t1	9718396	9722700	Lachesis_group3		8181976	8185898
Lachesis_group14		6124790	6125689	Lachesis_group20	Actinidia22728.t1	18523750	18524629
Lachesis_group20		18523750	18524629	Lachesis_group23	Actinidia22149.t1	3785646	3788269
Lachesis_group14	Actinidia30276.t1	8413081	8414806	Lachesis_group18	Actinidia13333.t1	2871811	2872639
Lachesis_group14	Actinidia10847.t1	4429121	4430028	Lachesis_group20	Actinidia31506.t1	12614584	12615692

Lachesis_group14	Actinidia10847.t1	4429121	4430028	Lachesis_group23	Actinidia02176.t1	6948609	6957579
Lachesis_group14	Actinidia27342.t1	5980472	5981873	Lachesis_group23		3992732	3993999
Lachesis_group14	Actinidia30276.t1	8413081	8414806	Lachesis_group27	Actinidia15738.t1	12052653	12058737
Lachesis_group17	Actinidia26137.t1	15595719	15599317	Lachesis_group20	Actinidia05650.t1	2647691	2648615
Lachesis_group17	Actinidia39895.t1	12909770	12911052	Lachesis_group21	Actinidia11342.t1	9458022	9458777
Lachesis_group17	Actinidia18633.t1	2497599	2499948	Lachesis_group22	Actinidia40302.t1	16914235	16915599
Lachesis_group17	Actinidia03260.t1	8198517	8204107	Lachesis_group22	Actinidia06788.t1	10554098	10559624
Lachesis_group20	Actinidia05230.t1	11599877	11600698	Lachesis_group23	Actinidia02044.t1	5274911	5276160
Lachesis_group20	Actinidia23158.t1	14843696	14845668	Lachesis_group24	Actinidia11629.t1	8941629	8948373
Lachesis_group23	Actinidia02046.t1	5284783	5287771	Lachesis_group3	Actinidia34076.t1	14726370	14727393
Lachesis_group23	Actinidia02044.t1	5274911	5276160	Lachesis_group3	Actinidia03102.t1	22666301	22667324
Lachesis_group3	Actinidia34076.t1	14726370	14727393	Lachesis_group6	Actinidia14518.t1	17709076	17731261
Lachesis_group23	Actinidia22309.t1	1679293	1679909	Lachesis_group27	Actinidia23090.t1	10375505	10380757
Lachesis_group2	Actinidia35241.t1	6532438	6533209	Lachesis_group6		3813413	3814114
Lachesis_group25	Actinidia16407.t1	11683578	11687114	Lachesis_group26	Actinidia10225.t1	4876400	4881334
Lachesis_group25	Actinidia29985.t1	17351873	17358995	Lachesis_group8		5050364	5052589
Lachesis_group11	Actinidia06105.t1	14293407	14296203	Lachesis_group3	Actinidia26237.t1	4786906	4788222
Lachesis_group21	Actinidia17567.t1	3235908	3237406	Lachesis_group3	Actinidia26237.t1	4786906	4788222
Lachesis_group2	Actinidia08804.t1	1706878	1708488	Lachesis_group23	Actinidia22349.t1	1208756	1209973
Lachesis_group2	Actinidia08804.t1	1706878	1708488	Lachesis_group27	Actinidia15778.t1	12572726	12573703
Lachesis_group2	Actinidia35451.t1	8857688	8861292	Lachesis_group24	Actinidia24184.t1	5567003	5568033
Lachesis_group2	Actinidia35451.t1	8857688	8861292	Lachesis_group9	Actinidia37093.t1	9273392	9274135
Lachesis_group24	Actinidia24184.t1	5567003	5568033	Lachesis_group9	Actinidia37093.t1	9273392	9274135
Lachesis_group22	Actinidia40245.t1	16208703	16209692	Lachesis_group3	Actinidia01945.t1	910965	914439
Lachesis_group22	Actinidia40245.t1	16208703	16209692	Lachesis_group3	Actinidia01945.t1	910965	914439
Lachesis_group2	Actinidia35366.t1	7885135	7887213	Lachesis_group5	Actinidia09751.t1	6315016	6323396
Lachesis_group2	Actinidia35366.t1	7885135	7887213	Lachesis_group9	Actinidia10747.t1	8075840	8078063
Lachesis_group1	Actinidia25441.t1	3375604	3376343	Lachesis_group13	Actinidia18228.t1	11187682	11188869
Lachesis_group0	Actinidia12165.t1	12684651	12685445	Lachesis_group16	Actinidia33496.t1	14283042	14283593
Lachesis_group0	Actinidia12165.t1	12684651	12685445	Lachesis_group23	Actinidia12663.t1	13668400	13669374
Lachesis_group0	Actinidia12165.t1	12684651	12685445	Lachesis_group23	Actinidia02153.t1	6703757	6705613
Lachesis_group13		3660887	3662356	Lachesis_group22	Actinidia03019.t1	7207873	7212608
Lachesis_group16	Actinidia33496.t1	14283042	14283593	Lachesis_group23	Actinidia12663.t1	13668400	13669374
Lachesis_group22	Actinidia03019.t1	7207873	7212608	Lachesis_group3		8181976	8185898
Lachesis_group17		18494213	18494622	Lachesis_group6	Actinidia34742.t1	1214115	1222845
Lachesis_group18	Actinidia30245.t1	16525712	16535313	Lachesis_group5	Actinidia39635.t1	21523925	21524553
Lachesis_group10		4568241	4572772	Lachesis_group15		2562437	2566821
Lachesis_group12		17749545	17762403	Lachesis_group15		2562437	2566821
Lachesis_group14	Actinidia27342.t1	5980472	5981873	Lachesis_group6	Actinidia04217.t1	15732612	15733637
Lachesis_group14	Actinidia27342.t1	5980472	5981873	Lachesis_group6	Actinidia08622.t1	8687752	8691964
Lachesis_group15		2562437	2566821	Lachesis_group16		3503932	3510044
Lachesis_group15		2562437	2566821	Lachesis_group21		1767552	1772059
Lachesis_group21	Actinidia31862.t1	17334658	17335761	Lachesis_group6	Actinidia08622.t1	8687752	8691964
Lachesis_group23		3992732	3993999	Lachesis_group6	Actinidia04217.t1	15732612	15733637

Lachesis_group23	Actinidia02153.t1	6703757	6705613	Lachesis_group6	Actinidia14407.t1	19273114	19277199
Lachesis_group23		3992732	3993999	Lachesis_group6	Actinidia08622.t1	8687752	8691964
Lachesis_group6	Actinidia08622.t1	8687752	8691964	Lachesis_group6	Actinidia04217.t1	15732612	15733637
Lachesis_group0	Actinidia07971.t1	26548386	26549003	Lachesis_group6	Actinidia20249.t1	21198987	21199763
Lachesis_group21	Actinidia31772.t1	16356508	16357569	Lachesis_group22	Actinidia31668.t1	4148197	4148801
Lachesis_group22	Actinidia25891.t1	2368463	2377890	Lachesis_group23	Actinidia02179.t1	7014600	7015896
Lachesis_group22	Actinidia24986.t1	3949357	3966476	Lachesis_group3		13681794	13695769
Lachesis_group22	Actinidia24986.t1	3949357	3966476	Lachesis_group3		22300737	22308726
Lachesis_group13		3660887	3662356	Lachesis_group24	Actinidia37282.t1	3979664	3983887
Lachesis_group24	Actinidia37282.t1	3979664	3983887	Lachesis_group3		8181976	8185898
Lachesis_group21	Actinidia00670.t1	15524105	15526957	Lachesis_group3	Actinidia00960.t1	21623392	21624269
Lachesis_group22	Actinidia40060.t1	13319531	13322279	Lachesis_group3	Actinidia04453.t1	3586023	3588178
