# Supplementary material for: Genome-Wide Identification of DREB Gene Family in Kiwifruit and Functional Characterization of Exogenous 5-ALA-Mediated Cold Tolerance via ROS Scavenging and Hormonal Signaling
Source: Plants (Basel). 2025 Aug 17;14(16):2560. doi: 10.3390/plants14162560 (PMC12389587; doi:10.3390/plants14162560)
Supplement: Supplementary file 1 [file plants-14-02560-s001.zip › Annexed Table S5 Pathways and gene names enriched for KEGG in.pdf]

5Annexed Table S5 Pathways and gene names enriched for KEGG in the kiwifruit DREB gene family

| KEGG_pathway | KO | gene number | Gene ID | Gene Name |
|---|---|---|---|---|
| Plant hormone signal transduction | ko04075 | 8 | Actinidia04453.t1、Actinidia25785.t2、Actinidia17188.t1、Actinidia09555.t1、Actinidia06476.t1、Actinidia06475.t1、Actinidia38946.t1、Actinidia40060.t1 | AcDREB24，AcDREB69，AcDREB76，AcDREB105，AcDREB111，AcDREB112，AcDREB125，AcDREB156 |
| MAPK signaling pathway-plant | ko04016 | 8 | Actinidia04453.t1、Actinidia25785.t2、Actinidia17188.t1、Actinidia09555.t1、Actinidia06476.t1、Actinidia06475.t1、Actinidia38946.t1、Actinidia40060.t1 | AcDREB24，AcDREB69，AcDREB76，AcDREB105，AcDREB111，AcDREB112，AcDREB125，AcDREB156 |
| Plant-pathogen interaction | ko04626 | 19 | Actinidia23663.t1、Actinidia03155.t1、Actinidia07617.t1、Actinidia07616.t1、Actinidia08622.t1、Actinidia04217.t1、Actinidia13662.t1、Actinidia39827.t1、Actinidia27342.t1、Actinidia14886.t1、Actinidia38372.t1、Actinidia27955.t1、Actinidia39895.t1、Actinidia05668.t1、Actinidia23158.t1、Actinidia11342.t1、Actinidia31862.t1、Actinidia22349.t1、Actinidia11629.t1 | AcDREB2，AcDREB34，AcDREB43，AcDREB44，AcDREB47，AcDREB49，AcDREB73，AcDREB92，AcDREB96，AcDREB100，AcDREB102，AcDREB116，AcDREB121，AcDREB135，AcDREB140，AcDREB143，AcDREB148，AcDREB159，AcDREB174 |
| Spliceosome | ko03040 | 9 | Actinidia25518.t1、Actinidia08910.t1、Actinidia29544.t1、Actinidia36843.t1、Actinidia00441.t1、Actinidia18299.t1、Actinidia28438.t1、Actinidia17778.t1、Actinidia04754.t1 | AcDREB12，AcDREB17，AcDREB56，AcDREB65，AcDREB82，AcDREB88，AcDREB110，AcDREB185，AcDREB187 |
