# Supplementary material for: Genome-Wide Identification of DREB Gene Family in Kiwifruit and Functional Characterization of Exogenous 5-ALA-Mediated Cold Tolerance via ROS Scavenging and Hormonal Signaling
Source: Plants (Basel). 2025 Aug 17;14(16):2560. doi: 10.3390/plants14162560 (PMC12389587; doi:10.3390/plants14162560)
Supplement: Supplementary file 1 [file plants-14-02560-s001.zip › Annexed Table S6 Web sites for bioinformatics databases and analysis.pdf]

Annexed Table S6 Web sites for bioinformatics databases and analysis

**Database and software name in Chinese**	**Name of Database and Software**	**Databases and software Web sites**
Kiwifruit Genome Database	Kiwifruit Genome Database 3.0	http://kiwifruitgenome.org/organism/5
Rice Genome Database	Rice Genome Annotation Project	RGAP , http://rice.plantbiology.msu.edu/index.shtml
Genome Database for Rosaceae	Rosaceae - GDR 1.0	https://www.rosaceae.org/
Ensembal Plants Database	Ensembal plants database	https://plants.ensembl.org/index.html
TAIR Database	TAIR - Arabidopsis	https://www.arabidopsis.org/index.jsp
National Center for Biotechnology Information	National Center for Biotechnology Information	https://www.ncbi.nlm.nih.gov/genbank/
Figshare Data Repository	Figshare data repository	https://figshare.com/projects/whole_genome_sequencing_and_assembly_of_Medicago_sativa/66380
Pfam - Shared Database - National Center for Genomics Data	Pfam-Database Commons-National Genomics Data Center	http://pfam.xfam.org/
Online Simple Modular Architecture Research Tool	Online Simple Modular Architecture Research Tool	http://smart.embl.de/
Hidden Markov Model	HMM model	https://www.ebi.ac.uk/Tools/hmmer/
MEGA11 Software	Molecular Evolutionary Genetics Analysis 11 Software	www.megasoftware.net
Chiplot Online Software	Chiplot Online Software	https://www.chiplot.online/
	Expasy ProtParam	https://web.expasy.org/protparam/
	WoLF PSORT	https://www.genscript.com/wolf-psort.html
MEME Online Analysis Tool	Multiple Em for Motif Elicitation	https://meme-suite.org/meme/index.html
PlantCARE Online Aanalysis Software	PlantCARE online analysis software	https://bioinformatics.psb.ugent.be/webtools/plantcare/html/
EggNOG-MAPPER Database	EggNOG-MAPPER database	http://eggnog-mapper.embl.de/
HISAT2 Software	HISAT2 software	http://ccb.jhu.edu/software/hisat2/index.shtml
STRING Protein Interaction Database	STRING: functional protein association networks	http://string-db.org/
