# Supplementary material for: Genome-Wide Identification of DREB Gene Family in Kiwifruit and Functional Characterization of Exogenous 5-ALA-Mediated Cold Tolerance via ROS Scavenging and Hormonal Signaling
Source: Plants (Basel). 2025 Aug 17;14(16):2560. doi: 10.3390/plants14162560 (PMC12389587; doi:10.3390/plants14162560)
Supplement: Supplementary file 1 [file plants-14-02560-s001.zip › Annexed Table S7 Fluorescence quantitative qRT.pdf]

Annexed table S7 Fluorescence quantitative qRT-PCR reaction procedure

Step	Time	Temperature	Number of cycle
PCR initial heat activation	2 min	95 ℃	1
Denaturation	5 s	95 ℃	40
Combined annealing/extension	30 s	60 ℃	
Melting curve analysis			
