# Supplementary material for: Genome-Wide Identification of DREB Gene Family in Kiwifruit and Functional Characterization of Exogenous 5-ALA-Mediated Cold Tolerance via ROS Scavenging and Hormonal Signaling
Source: Plants (Basel). 2025 Aug 17;14(16):2560. doi: 10.3390/plants14162560 (PMC12389587; doi:10.3390/plants14162560)
Supplement: Supplementary file 1 [file plants-14-02560-s001.zip › Annexed Table S8 The primers information for quantitative real-time PCR.pdf]

Annexed Table S8 The primers information for quantitative real-time PCR

**Gene**		**sequence（5'-3'）**	**product size**
Actinidia10308	F	CTCAACTTCCCCAACCTCCG	145 bp
	R	GCTTCTTTGCGTCAAGGCTC	
Actinidia25785	F	GGAATCAGGGTATGGCTCGG	111 bp
	R	CACCGGAAAATTGAGCACCG	
Actinidia07597	F	TTGCAGCAGTACCCCTTTGC	104 bp
	R	TCCTGATTCATCCTCGCCGAC	
Actinidia31506	F	AGGAGAGGCAGCACTACAGA	194 bp
	R	TTCGAGGGGGAAGTTGAGGA	
Actinidia35241	F	GCGCGTCCGATCTTCTTTG	157 bp
	R	GGATGTTATCGACGACGGAGG	
Actinidia13612	F	ATTTTTGCGGCGGTGTTCCT	113 bp
	R	AATCTCCGCCGCGAATTTCC	
Actinidia31667	F	AATTCGAGACGAAGCCCCAA	100 bp
	R	AAATTCGCCGCCGGAACC	
Actinidia20249	F	CGGCCAGGGATATTCAGACT	126 bp
	R	ACTCCGGCAACTCAATCTCAC	
Actinidia10847	F	GAAGCCTCCGGTGACGAAA	101 bp
	R	TGTAGTGCTGCCTCTCCTCT	
Actinidia14855	F	AAAGACGGTGACGCCGAG	118 bp
	R	GTTAAGGAGCACGCCTTTCG	
Actinidia10399	F	ACGGTTTGGCTCGATATGCTA	167 bp
	R	GAGCAAGACATGGTGGTCGT	
Actinidia31862	F	AAGAAAGTGCGACTCTGGCT	118 bp
	R	GCGGAGTGATGAAGTTGGTC	
Actinidia39635	F	GTCGTGAGAGGATCTGGCTG	151 bp
	R	GGAGTGAGGGACCGACCG	
Actinidia16620	F	ACTCGTACTGGAACAGCAGC	180 bp
	R	GAAACCCGGCAACACTGGAC	
Actinidia04464(Actin)	F	TGGTCCACCAGGAAGGTCTA	197 bp
	R	TGTCTGATGATGCCTCTGGG	
