# Supplementary material for: Genome-Wide Identification of DREB Gene Family in Kiwifruit and Functional Characterization of Exogenous 5-ALA-Mediated Cold Tolerance via ROS Scavenging and Hormonal Signaling
Source: Plants (Basel). 2025 Aug 17;14(16):2560. doi: 10.3390/plants14162560 (PMC12389587; doi:10.3390/plants14162560)
Supplement: Supplementary file 1 [file plants-14-02560-s001.zip › Annexed Table S9 Sequence of cold-resistant DREB gene family proteins.pdf]

Annexed Table S9 Sequence of cold-resistant DREB gene family proteins

>Os04g35030.1
MAAASGEKEEEEKKLQERAPIRRTAWMLANFVVLFLLLALLVRRATAADAEERGVGGAA
WRVAFACEAWFAFVWLLNMNAKWSPARFDTYPENLAGRCGAAHRPRKSSCISGHLDLM
RRQCALMQDRRAAGGRHVRDDGGPGARAAGGDGEQGALAARRRLLPGRRRRRRRRL
ACYVSDDGCSPVTYYALREAAGFARTWVPFCRRHGVAVRAPFRYFASAPEFGPADRKFLD
DWTFMKSEYDKLVRRIEDADETTLLRQGGGEFAEFMDAKRTNHRAIVKVIWDNNSKNRI
GEEGGFPHLIYVSREKSPGHHHHYKAGAMNALTRVSAVMTNAPIMLNVDCDMFANDPQ
VVLHAMCLLLGFDDEISSGFVQVPQSFYGDLKDDPFGNKLEVIYKKLLGGVAGI
>Os05g35010.1
MLSLSSEKIKETSARSARSAATAMEIELSPVLLLLPFLLLGFLYLTGGVLRSGGNARRRLAP
APRGLPVIGNLHQVGALPHRALRALAAATGAPHLLRLRLGHVTALVASSPAAAAAVMREH
DHVFATRPYFRTAEILTYGFKDLVFAPYGEHWRHARRLCSEHVLSAARSHRYGPMREQEV
ALLVNAIRTEAAAAAVDVSKALYAFTNAVICRAVSGRLSREDEGRSELFRELIEENATLLGG
FCVGDYFPALAWADAFLSGFAARACRNLRRWDELLEEVIAEHEARLRGGDDGGGEEHRE
EDFVDVLLALQEESQRHDGSFKLTRDIIKSLLQDMFAAGTDTSFITLEWAMSELVKNPAAM
RKLQDEVRRGGGATTAATPYLKAVVKETLRLHPPVPLLVPRECARDTDDDATVLGYHVA
GGTRVFVNAWAIHRDAGAWSSPEEFRPERFLPGGGEAEAMDLRGGHFQLVPFGAGRRVC
PGMQFALATVELALASLVRLFDWEIPPPGELDMSDDPGFTVRRRIPLRLVAKPVGSEDDK
>Os06g03670.1
MEYYEQEEYATVTSAPPKRPAGRTKFRETRHPVYRGVRRRGPAGRWVCEVREPNKKSRI
WLGTFATAEAAARAHDVAALALRGRGACLNFADSARLLRVDPATLATPDDIRRAAIELAES
CPHDAAAAAASSSAAAVEASAAAAPAMMMQYQDDMAATPSSYDYAYYGNMDFDQPSY
YYDGMGGGGEYQSWQMDGDDDGGAGGYGGGDVTLWSY
>ABB36646.1
MAKPSSEKPEEHSDSKYYKGVRKRKWGKWVSEIRLPNSRQRIWLGSYDTPEKAARAFDA
AMFCLRGRNAKFNFPDNPPDIAGGTSMTPSQIQIAAAQFANAGPHEGHSGRPEHPPMESPS
PSVSEGTIQTDSDVPTLNGSVTDLFTPVGSSGYASDYGIFPGFDDFSGDFYVPEMPNVNYG
EENGEGFIVDESFLWNF
>XP002273838.1
MSSGVIERKRKSRSRRSGPNSVAETLARWKQYNDILDSVRKAPAKGSKKGCMKGKGGPE
NSICGYRGVRQRTWGKWVAEIREPNRGSRLWLGTFPTAIEAALAYDEAARAMYGSSARL
NLPNYTTSLKDSSSAPTTSVSDSTTTTSNYSEVCAYEDSKKPVLPSIKHESGEGESGISGGM
LSAVVKAEPATPVSLVTQGGGNDPVNVGNEPVDAMKLQHEENGHSLDAMYFKNEDGGQ
DFLEGFPMDEMFDVDEFLRAIDSDPLASYGTRQELGHDSGQVGSFETDNMQWEKPTDLS
YQLQNPDAKLLGSLNHMEQVPSDFDYCYGFLQPGKQLDPCIGLNDQGLLDLELSDMGF
>ABQ59086.1
MDTISRQLSDSAEKPESSSESVTTRSQLTSFSDEEVILASSTPKKRAGRRVFNETRHPVYRG
VRRRNNDKWVCEMREPNKKKSRIWLGAYPTAEMAARAHDVAALAFRGRLACLNFADSA
WRLPVPASTDSVDIRRAAAEAAETFRPAEFGGVSESGDDEKESKKMEGEKDCGCAEQSD
CGRAEQSDCGGAEQSGSSFYLDEEEMFAMPRLLDSMAEGLLLSPPRRSAGSNMNWDDM
GSNDDDVNLWSFSK
>ACB20695.1

MDSSKKNVFSHYSDSLPSGSGFSSKEVAECSSASDRGTGAHPAAICSDDELLLASRNPKKR
GGRKKVKETRHPVYRGVRWRNAHTWVCEVREPNKKSRIWLGTFPTAEMAARAHDVAAI
ALRNRMACVNFADSVWRLPVPASLDPKDIQKAAAEAAEMFRPPLLEEKKDVPGDVAQPE
EDATLITTATTTATETATETTMPENLLFMDDEAIFGMPGLLANMAEGLMLPPPPPHSEGGD
WYEGDDAELGDDVSLWSFSI

>MS.gene62760.t1

MINTNNSSYSHSIYSKDFSPFDASSPDSEVRLAASNPKKRAGRKIFKETRHPVYRGVRKRN
LDKWVCEMREPNTKTRIWLGTFPTPEMAARAHDVAAMALRGRYACLNFADSMWRLPIP
ASSSIKDIQKAATKAAEAFRPDNTLMTNDIDTVVATVATKELNMFCVEVEEEQEMLNMPE
LWRNMALMSPTHSFEYHEYEDIHVQDFQDDEDFKNKSVTTIWAVTAIEVHSPHFTIMYRIV
IVAVTANPAFLFSLFSSWQKKVQKKNE

>MS.gene30844.t1

MFREGQLAASCPKKPAGRKKFKETRHPVYRGVRKRNLDKWVCEMREPNKKTRIWLGTF
PTAEMAARAHDVAAMALRGRYACLNFADSVWRLPTPASAETKDIQRAAAEAAEAFRPDK
TLTTTDIDTVVAVVVAVEEEEEVLNMFCVEVEEEEEVLNMPELWRNMALMSPTHSLVHEY
EDFDVQFQDEEVSLWNF

>AT4G25490.1

MNSFSAFSEMFGSDYEPQGGDYCPTLATSCPKKPAGRKKFRETRHPIYRGVRQRNSGKW
VSEVREPNKKTRIWLGTFQTAEMAARAHDVAALALRGRSACLNFADSAWRLRIPESTCAK
DIQKAAAEAALAFQDETCDTTTTNHGLDMEETMVEAIYTPEQSEGAFYMDEETMFGMPT
LLDNMAEGMLLPPPSVQWNHNYDGEGDGDVSLWSY*

>AT4G25470.1

MNSFSAFSEMFGSDYESPVSSGGDYSPKLATSCPKKPAGRKKFRETRHPIYRGVRQRNSG
KWVCELREPNKKTRIWLGTFQTAEMAARAHDVAAIALRGRSACLNFADSAWRLRIPESTC
AKEIQKAAAEAALNFQDEMCHMTTDAHGLDMEETLVEAIYTPEQSQDAFYMDEEAMLG
MSSLLDNMAEGMLLPSPSVQWNYNFDVEGDDDVSLWSY*

>AT4G25480.1

MNSFSAFSEMFGSDYESSVSSGGDYIPTLASSCPKKPAGRKKFRETRHPIYRGVRRRNSGK
WVCEVREPNKKTRIWLGTFQTAEMAARAHDVAALALRGRSACLNFADSAWRLRIPESTC
AKDIQKAAAEAALAFQDEMCDATTDHGFDMEETLVEAIYTAEQSENAFYMHDEAMFEM
PSLLANMAEGMLLPLPSVQWNHNHEVDGDDDDVSLWSY*

>AT5G05410.1

MAVYDQSGDRNRTQIDTSRKRKSRSRGDGTTVAERLKRWKEYNETVEEVSTKKRKVPAK
GSKKGCMKGKGGPENSRCSFRGVRQRIWGKWVAEIREPNRGSRLWLGTFPTAQEAASAY
DEAAKAMYGPLARLNFPRSDASEVTSTSSQSEVCTVETPGCVHVKTEDPDCESKPFSGGV
EPMYCLENGAEEMKRGVKADKHWLSEFEHNYWSDILKEKEKQKEQGIVETCQQQQQDS
LSVADYGWPNDVDQSHLDSSDMFDVDELLRDLNGDDVFAGLNQDRYPGNSVANGSYRP
ESQQSGFDPLQSLNYGIPPFQLEGKDGNGFFDDLSYLDLEN*
